# Systematic analysis of the burden of ischemic stroke attributable to high LDL-C from 1990 to 2021

**DOI:** 10.3389/fneur.2025.1547714

**Published:** 2025-04-04

**Authors:** Jiahao Tang, Guoyang Zhou, Shunan Shi, Yuexin Lu, Lin Cheng, Jianping Xiang, Shu Wan, Ming Wang

**Affiliations:** ^1^The Second School of Clinical Medicine, Zhejiang Chinese Medical University, Hangzhou, China; ^2^Brain Center, Zhejiang Hospital, Hangzhou, Hangzhou, China; ^3^Zhejiang Province Engineering Research Center for Precision Medicine in Cerebrovascular Diseases, Zhejiang Hospital, Hangzhou, China; ^4^ArteryFlow Technology Co., Ltd., Hangzhou, China

**Keywords:** global burden of disease, high LDL-C, ischemic stroke, socio-demographic index, inequality analysis

## Abstract

**Background:**

Low-density lipoprotein cholesterol (LDL-C) is a public health concern linked to ischemic stroke. The study aimed to describe the epidemiological characteristics of ischemic stroke attributable to high LDL-C from 1990 to 2021.

**Methods:**

In this study, we analyzed data from the Global Burden of Disease 2021 to present trends in ischemic stroke related to high LDL-C over the past 30 years. The relationship between disease burden and the Socio-Demographic Index (SDI) was examined. To assess international health disparities, we applied the Slope Index of Inequality (SII) and the Concentration Index (CI). Furthermore, we conducted a frontier analysis to identify areas for improvement and developmental gaps among nations, and employed the Bayesian Age-Period-Cohort (BAPC) model to forecast the disease burden for the next 15 years.

**Results:**

In 2021, the incidence of ischemic stroke attributed to high LDL-C significantly increased compared to 1990, with a more pronounced growth rate in males. The burden mainly affects individuals aged 70 to 84. Analysis using the age-period-cohort model indicates that mortality rates and DALYs rise with age, while period and cohort effects exhibit a gradual decline. Across different SDI regions, trends generally follow a similar downward path, with a narrowing gap in disease burden among regions with varying SDI levels. However, the disease burden in high SDI countries remains significant, indicating potential for reduction. Predictions for the next 15 years suggest that while the global disease burden will decrease, there may be an increase among individuals under 55.

**Conclusion:**

Compared to 1990, the overall age-standardized burden of ischemic stroke related to high LDL-C has been controlled. However, disparities persist across different SDI regions. We have observed an increasing burden among younger populations. Consequently, countries and regions must adopt new measures tailored to their SDI levels, with a specific emphasis on younger individuals. It is essential to develop prevention and treatment strategies aimed at high-risk groups.

## 1 Introduction

Ischemic stroke represents a serious global health challenge, ranking among the top causes of long-term disability and mortality worldwide (1). Historically, ischemic stroke has been a significant contributor to overall disease burden, with notable shifts in its epidemiology over the past three decades. From 1990 to 2021, the disease burden showed a declining trend, with the average annual percent change (AAPC) in the age-standardized incidence rate (ASIR), mortality rate (ASMR), and disability-adjusted life years (ASDR) for ischemic stroke being −0.57 (95% CI: −0.66 to −0.48), −1.60 (95% CI: −1.81 to −1.39), and −1.37 (95% CI: −1.53 to −1.20), respectively (2). Over these 30 years, the burden of ischemic stroke attributable to metabolic risks, environmental and occupational risks, and behavioral risks all showed a declining trend. Among these, metabolic-related factors remained the primary risk factors for ischemic stroke, with a population attributable fraction (PAF) of 76.88% (2). Among these, high low-density lipoprotein (LDL) cholesterol had a PAF of 25.78%, making it a significant cause of ischemic stroke among metabolic factors, second only to high systolic blood pressure (2).

Although the age-standardized disease burden of ischemic stroke has decreased, there were still 7,804,449 cases of ischemic stroke globally in 2021 (95% UI, 6,719,760–8,943,692), imposing a significant economic burden on public health systems (2). Additionally, due to more advanced medical facilities in developed countries and regions compared to the poorer infrastructure and medical conditions in developing and underdeveloped areas, there are significant disparities in disease burden across different regions. Furthermore, there are differences in disease burden across different age groups and genders. Therefore, a more detailed analysis of the disease burden related to ischemic stroke risk factors is still necessary, which may provide new insights for reducing this burden.

Hyperlipidemia, particularly low-density lipoprotein cholesterol (LDL-C), is a well-established risk factor for cardiovascular disease and a key driver of ischemic stroke (3–6). Among ischemic stroke cases, high LDL cholesterol (LDL-C) has a population attributable fraction (PAF) of 25.78%, making it a significant cause of ischemic stroke among metabolic factors, second only to high systolic blood pressure (2). Through lipid-lowering drug interventions, dyslipidemia is a modifiable risk factor for ischemic stroke, and its control can prevent the occurrence and recurrence of stroke. Studies have shown that actively lowering cholesterol levels with high doses of atorvastatin can reduce the incidence of stroke by 33% and recurrent stroke by 16% in patients with carotid artery stenosis (7).

Despite ongoing interventions to manage cholesterol levels, the efficacy of these efforts in reducing ischemic stroke burden due to high LDL-C remains uncertain (8). In a study involving 213,380 individuals, only 51.8% of very high-risk (VHR) patients with atherosclerotic cardiovascular disease (ASCVD) and type 2 diabetes (DM2) achieved the lipid-lowering target (LDL-C < 70 mg/dl) (9). As economic development progresses, high-energy, high-cholesterol, and high-fat diets are becoming more common among people, coupled with a lack of exercise and increased life stress, which may further increase the burden of ischemic stroke due to dyslipidemia.

To further assess the disease burden of ischemic stroke caused by dyslipidemia, this study utilizes GBD 2021 data to explore the long-term trends, regional disparities, and future projections of ischemic stroke burden related to high LDL-C from 1990 to 2021. By providing these insights, we aim to assist healthcare professionals and policymakers in developing effective strategies to mitigate the health risks and burden associated with ischemic stroke.

## 2 Method

### 2.1 Data source

GBD utilizes standardized methodologies to estimate health impacts associated with 371 diseases, injuries, and 88 risk factors across 204 countries and territories, 21 regions, and 7 super-regions (10–12). In our study, we focus on death counts and disability-adjusted life years (DALYs) as primary indicators of disease burden. DALYs combine years lived with disability (YLDs) and years of life lost (YLLs) (10). YLDs were calculated based on the prevalence of disease-specific sequelae, adjusted by disability weights, while YLLs consider cause-specific mortality and standard life expectancy. Our analysis covers both crude and age-standardized rates (ASR), stratified by demographic and socio-economic variables including age, sex, region, and the Socio-demographic Index (SDI).

### 2.2 Statistics analysis

#### 2.2.1 Joinpoint regression analysis

We used Joinpoint software (version 5.1.0.0; National Cancer Institute, Rockville, Maryland, USA) to create an analytical model for assessing disease burden trends. This model applies segmented regression to analyze the temporal patterns of disease distribution, fitting and optimizing trends for each segment. This approach allows for a thorough examination of disease variation across different intervals globally. The annual percentage change (AAPC) and its 95% confidence interval (CI) were calculated for each region, with statistical significance assessed at a *p*-value threshold of 0.05.

#### 2.2.2 Age-period-cohort model

The age-period-cohort (APC) model aims to reveal the effects of age-related biological, technological, and social factors on disease trends. Compared to traditional epidemiological analyses, the APC model can independently analyze the effects of age, period, and cohort, and is therefore widely used in descriptive epidemiological studies (13). Our research focuses on indicators such as longitudinal age curves, period relative risks (RR), and cohort relative risks (RR). The longitudinal age curves are based on the fitted longitudinal age-specific rates from the reference cohort, adjusted for period deviations. Relative risk measures the age-specific rates for each period (or cohort) relative to the reference period (or cohort). When the RR value is greater than 1, it indicates a higher disease risk in that period (or cohort) compared to the reference period (or cohort). Through these analyses, we can gain a more comprehensive understanding of the dynamic changes in disease burden and provide scientific evidence for formulating effective public health policies. To achieve this, we incorporated data from the 2021 GBD database, which includes epidemiological information from the past 30 years. We categorized age and year into 5-year intervals, resulting in 15 age subgroups and 6 time periods. Model development and analysis were conducted in R (version 4.4.1) using the Epi package.

#### 2.2.3 Socio-demographic index

The Socio-demographic Index (SDI) is a comprehensive measure of development based on fertility rates, educational attainment, and income levels. It represents the geometric mean of the per capita lagged distributed income, average years of education, and fertility rate of women under 25 years old in a region, with values ranging from 0 to 1 (10). Current research has revealed an association between disease burden and SDI (14, 15). In this study, we explore the relationship between ischemic stroke burden from high LDL-C and socio-economic development, dividing SDI into five levels: low, low-middle, middle, middle-high, and high.

#### 2.2.4 Cross-country inequality analysis

The Slope Index of Inequality (SII) is a measure that quantifies absolute inequality in health indicators between the most advantaged and the most disadvantaged subgroups within a population. It takes into account the entire distribution of socioeconomic factors such as education or wealth through a weighted regression model. The Concentration Index (CI), on the other hand, quantifies relative inequality by indicating the degree to which health indicators are concentrated among disadvantaged or advantaged groups. Both SII and CI are standard indicators recommended by the World Health Organization (WHO) for quantifying distributional inequality of disease burden (16).

The SII is calculated using a national rate regression model based on a relative position scale related to the SDI (defined as the midpoint of the population range ranked by cumulative SDI). The CI is calculated through numerical integration under the Lorenz curve, which plots the cumulative proportion of stroke disease burden against the cumulative population distribution, sorted by socioeconomic development level (17, 18). This provides a measure of relative inequality. Smaller absolute values of both the SII and CI indicate a more equitable distribution of disease burden.

#### 2.2.5 Method for forecasting stroke burden beyond 2021

Our projections for future disease burden employed the Bayesian Age-Period-Cohort (BAPC) model, which utilized integrated nested Laplace approximations for predictive analysis (15). The BAPC model is recognized for its accuracy in epidemiological forecasting, outperforming models like Auto-Regressive Integrated Moving Average (ARIMA) (19–21). In our study, we used this model to forecast the ischemic stroke burden due to high LDL-C from 2022 to 2036. Through forecasting, we can analyze the feasibility of current health policies, thereby providing important guidance for policy adjustments.

The data in our study include 95% uncertainty intervals (UI) and confidence intervals (CI). Death counts and DALYs are presented in thousands, while ASMR and ASDR are per 100,000 population. All statistical analyses and result visualizations were conducted in R (version 4.4.1), with statistical significance defined as *p* < 0.05.

## 3 Results

### 3.1 Global trends

In 2021, there were approximately 936.19 × 10^3^ (95% UI: 299.54 × 10^3^ to 1,614.29 × 10^3^) mortality cases of ischemic stroke caused by high LDL-C, representing 43.04% increase compared to 1990. In 2021, the number of mortality cases for females reached 474.81 × 10^3^ (95% UI: 148.89 × 10^3^ to 836.96 × 10^3^), slightly exceeding the male cases of 461.38 × 10^3^ (95% UI: 150.64 × 10^3^ to 786.28 × 10^3^). Notably, the number of deaths among males increased by 61.15% compared to 1990, a growth rate nearly double that observed in females (28.96%). Throughout the study period, the ASMR for ischemic stroke exhibited a significant downward trend, with an AAPC of −1.80 (95% CI: −2.00 to −1.60). Gender analysis revealed that the ASMR for females (10.12 per 100,000; 95% UI: 3.18–17.81) was lower than that for males (12.81 per 100,000; 95% UI: 4.09–22.14), with a more pronounced decline observed in females (AAPC: -2.10; 95% CI: −2.30 to −1.80) compared to males (AAPC: -1.50; 95% CI: −1.70 to −1.30) (Table 1; Additional file 1: Figure S2A).

**Table 1:** Global deaths and DALYs of Ischemic stroke from 1990 to 2021.

Year	Both	Male	Female
1990			
The number of Deaths/1000 (95% UI)	654.5 (210.16, 1103.89)	286.32 (96.44, 474.75)	368.19 (115.9, 634.21)
The number of DALYs/1000 (95% UI)	14512.49 (5187.37, 23068.20)	6968.16 (2608.75, 10943.78)	7544.33 (2652.24, 12174.29)
ASMR/100,000 persons (95% UI)	20.03 (6.28,34.56)	20.36 (6.41,34.84)	19.4 (6.04,33.80)
ASDR/100,000 persons (95% UI)	390.89 (133.81, 632.33)	412.18 (144.62, 665)	368.47 (126.36, 599.97)
2021			
The number of Deaths/1000 (95% UI)	936.19 (299.54, 1614.29)	461.38 (150.64, 786.28)	474.81 (148.89, 836.96)
The number of DALYs/1000 (95% UI)	20977.42 (7409.04, 34664.41)	11039.93 (3969.6, 18176.49)	9937.49 (3435.54, 16487.97)
ASMR/100,000 persons (95% UI)	11.38 (3.62,19.65)	12.81 (4.09,22.14)	10.12 (3.18,17.81)
ASDR/100,000 persons (95% UI)	246.42 (86.47, 406.28)	280.47 (98.55, 469.59)	215.42 (75.19, 355.77)
1990–2021			
Deaths (%)	43.04	61.15	28.96
DALYs (%)	44.55	58.43	31.72
AAPC of ASMR (95% CI)	−1.80 (−2.00–−1.60)	−1.50 (−1.70–−1.30)	−2.10 (−2.30–−1.80)
AAPC of ASDR (95% CI)	−1.50 (−1.70–−1.30)	−1.20 (−1.40–−1.00)	−1.70 (−1.90–−1.50)

DALYs, Disability-Adjusted Life Years; ASMR, Age-Standardized Mortality Rate; ASDR, age-standardized disability-adjusted life-year rate; AAPC, annual average percent change; UI, uncertainty interval; CI, confidence interval.

Globally, the number of DALYs increased by 44.55% compared to 1990, reaching 20,977.42 × 10^3^ (95% UI: 7,409.04 × 10^3^ to 34,664.41 × 10^3^) in 2021. However, the ASDR fell, with an AAPC of −1.50 (95% CI: −1.70 to −1.30). This decline was more pronounced among females, with an AAPC of −1.70 (95% CI: −1.90 to −1.50), compared to an AAPC of −1.20 (95% CI: −1.40 to −1.00) among males (Table 1; Additional file 1: Figure S2B).

### 3.2 Region trends

**Figure 1:**
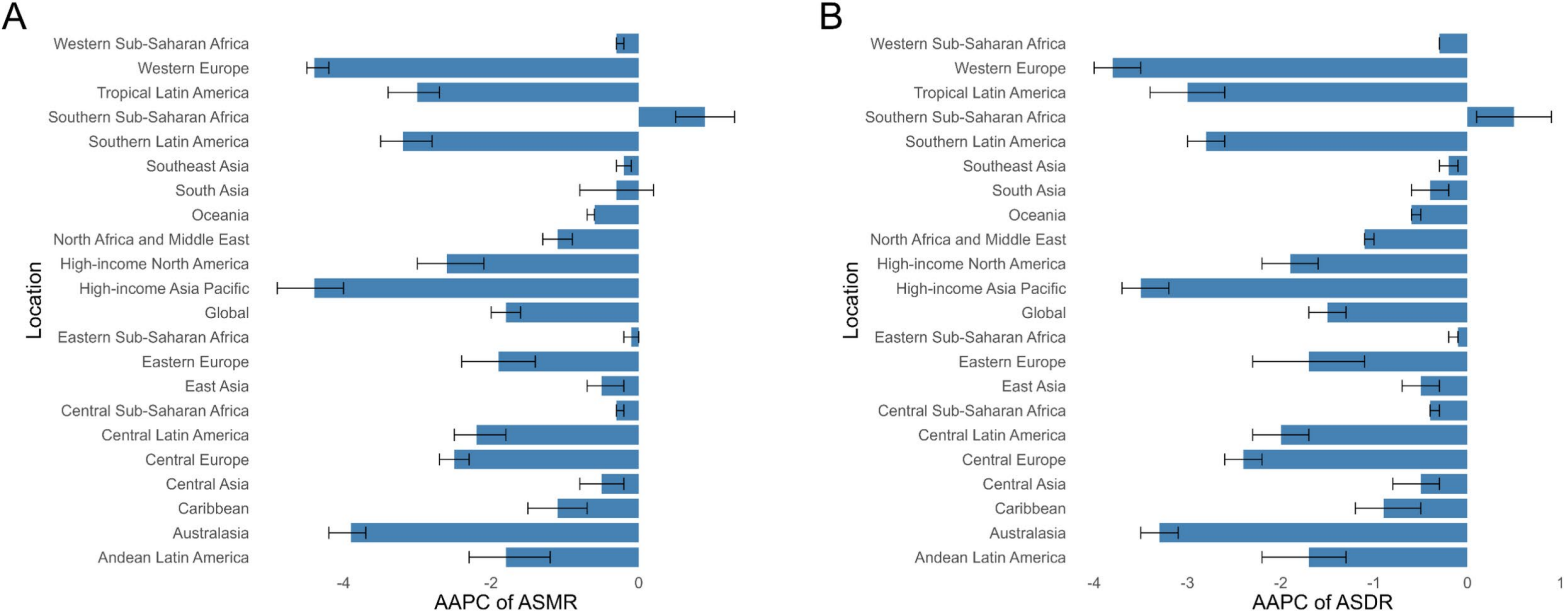
AAPCs of the ASRs for Ischemic Stroke attributable to high LDL-C. **(A)** AAPCs of the ASMR for Ischemic Stroke attributable to high LDL-C in 21 regions and global. **(B)** AAPCs of the ASDR for Ischemic Stroke attributable to high LDL-C in 21 regions and global. ASR age-standardized rate, ASMR Age-Standardized Mortality Rate, ASDR age-standardized disability-adjusted life-year rate, AAPC annual average percent change.

In 2021, the region with the highest number of ischemic stroke deaths attributable to high LDL-C was East Asia, with 307.72 × 10^3^ cases (95% UI: 95.03 × 10^3^ to 538.67 × 10^3^). Eastern Europe exhibited the highest ASMR at 32.34 per 100,000 (95% UI: 11.13–53.31 per 100,000), followed by Central Europe at 21.72 per 100,000 (95% UI: 6.92–36.51 per 100,000) and Central Asia at 21.28 per 100,000 (95% UI: 6.57–37.34 per 100,000) (Additional file 1: Tables S1, S2). All regions have shown a downward trend in ASMR except Southern Sub-Saharan Africa, with the High-Income Asia Pacific and Western Europe experiencing the most significant declines in AAPC, at −4.40 (95% CI: −4.50 to −4.20) and − 4.40 (95% CI: −4.90 to −4.00) (Additional file 1: Tables S3; Figure 1A). Furthermore, it can be observed that a larger ASMR reduction in females than in males in most regions (Additional file 1: Tables S3).

Similar to mortality rates, East Asia also recorded the highest number of DALYs in 2021, at 7,056.8 × 10^3^ cases (95% UI: 2,385.26 × 10^3^ to 11,732.69 × 10^3^). Eastern Europe reported the highest ASDR at 704.08 per 100,000 (95% UI: 266.76 to 1,106.72) (Additional file 1: Tables S1, S2). Consistent with the ASMR trends, the ASDR also showed a downward trend across all regions except for Southern Sub-Saharan Africa, with a more significant decline observed in females (Additional file 1: Tables S3; Figure 1B).

### 3.3 Country trends

**Figure 2:**
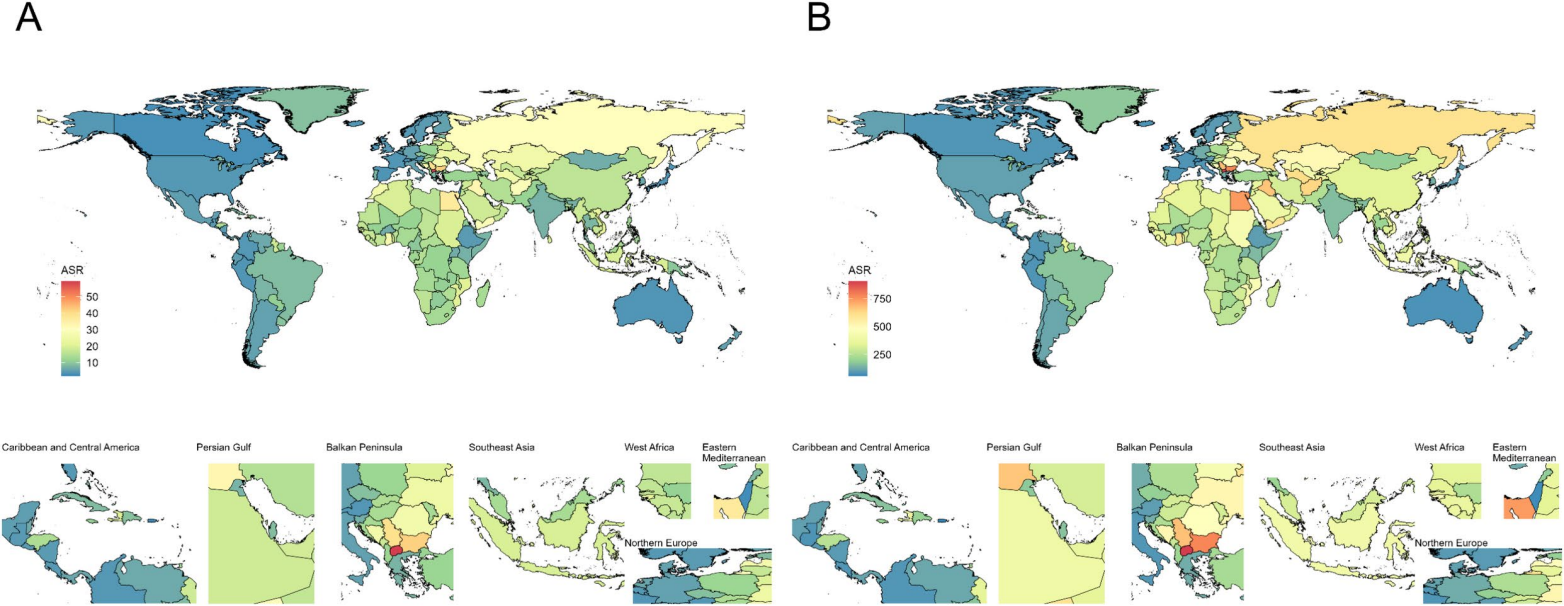
Age-standardized deaths and DALYs rate of ischemic stroke attributable to high LDL-C per 100,000 population in 2021, by country. **(A)** Age-standardized deaths rate; **(B)** Age-standardized DALYs rate. DALYs Disability-Adjusted Life Years.

In 2021, China (300.05 × 10^3^ cases; 95% UI: 92.52 × 10^3^ to 527.46 × 10^3^), Russia (75.13 × 10^3^ cases; 95% UI: 24.89 × 10^3^ to 124.87 × 10^3^), and India (74.25 × 10^3^ cases; 95% UI: 25.18 × 10^3^ to 131.56 × 10^3^) were reported to have the highest mortality numbers (Additional file 1: Tables S4). The highest ASMRs were observed in North Macedonia (59.37 per 100,000; 95% UI: 17.33 to 106.07 per 100,000), the Republic of Bulgaria (41.87 per 100,000; 95% UI: 13.02 to 71.01 per 100,000), and the Republic of Serbia (39.72 per 100,000; 95% UI: 11.66 to 70.16 per 100,000) (Additional file 1: Tables S5; Figure 2A). The Kingdom of Lesotho (AAPC = 1.70; 95% CI: 1.20 to 2.20), the United Republic of Tanzania (AAPC = 1.60; 95% CI: 1.50 to 1.80), and Montenegro (AAPC = 1.50; 95% CI: 0.80 to 2.30) were the countries showed the most significant increases in ASMR, whereas Luxembourg (AAPC = −5.70; 95% CI: −6.40 to −5.00), Portugal (AAPC = −5.70; 95% CI: −6.10 to −5.30), and Singapore (AAPC = −6.90; 95% CI: −7.80 to −5.90) experienced sharp declines (Additional file 1: Tables S6).

For DALYs, China (6850.57 × 10^3^ cases; 95% UI: 2313.2 to 11418.17), India (1818.15 × 10^3^ cases; 95% UI: 669.98 to 3090.36), and Russia (1456.6 × 10^3^ cases; 95% UI: 522.83 to 2317.35) (Additional file 1: Tables S4) ranked highest. In terms of ASDR, North Macedonia (905.74 per 100,000; 95% UI: 284.45 to 1565.26) and Bulgaria (775.22 per 100,000; 95% UI: 272.99 to 1271.21) remained the top two countries (Figure 2B; Additional file 1: Tables S5). Lesotho (AAPC = 1.80; 95% CI: 1.40 to 2.10), Zimbabwe (AAPC = 1.40; 95% CI: 1.00 to 1.80), and Tanzania (AAPC = 1.40; 95% CI: 1.30 to 1.60) demonstrated the fastest ASDR growth, while Luxembourg (AAPC = −5.40; 95% CI: −6.00 to −4.90), Singapore (AAPC = −5.40; 95% CI: −5.90 to −4.80), and Portugal (AAPC = −5.60; 95% CI: −5.90 to −5.20) showed the steepest declines, mirroring mortality trends (Additional file 1: Tables S6).

### 3.4 Global trends by SDI

**Figure 3:**
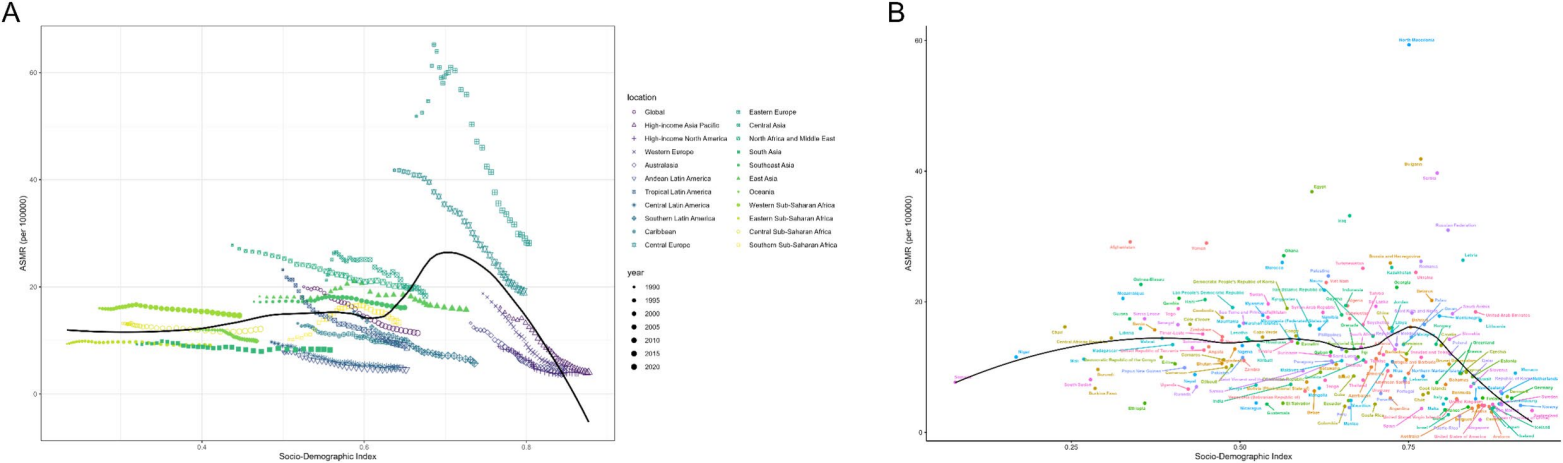
ASMRs for ischemic stroke attributable to high LDL-C of 21 regions and 204 countries and territories by SDI. **(A)** ASMRs for ischemic stroke attributable to high LDL-C of 21 regions from 1990 to 2021 according to the SDI. **(B)** ASMRs for ischemic stroke attributable to high LDL-C of 204 countries and territories in 2021 according to the SDI. ASMR Age-Standardized Mortality Rate, SDI Socio-Demographic Index.

**Figure 4:**
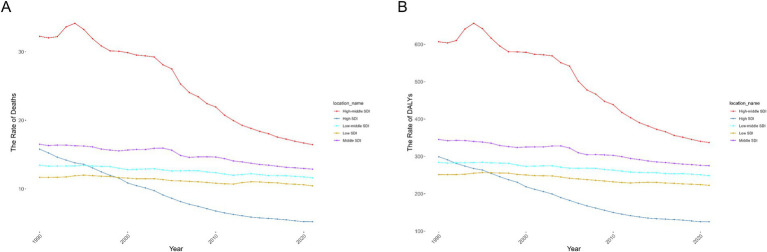
The age-standardized rates due to Ischemic Stroke attributable to high LDL-C during 1990–2021 by SDI. **(A)** The ASMR of Ischemic Stroke attributable to high LDL-C during 1990–2021 by SDI. **(B)** The ASDR of Ischemic Stroke attributable to high LDL-C during 1990–2021 by SDI. ASMR age-standardized deaths rate, ASDR age-standardized disability-adjusted life-year rate, SDI Socio-Demographic Index.

The regions with middle to high SDI levels showed the majority of the burden of deaths and DALYs (Additional file 1: Table S7). At the regional level, there was a negative correlation between disease burden and SDI (Figure 3A; Additional file 1: Figure S3A). Nationally, the burden first rose with increasing SDI levels, peaked, and then declined in high SDI areas (Figure 3B; Additional file 1: Figure S3B). With the changes in ASMR and ASDR from 1990 to 2021, the overall burden of disease showed a declining trend (Figure 4; Table 2). The burden of disease in the middle and middle-high SDI regions have decreased significantly, while the decline in other regions is slight. Furthermore, despite the noticeable decreases in ASMR and ASDR in middle-high SDI regions, the numbers of deaths and DALYs remained at a high level, indicating a substantial burden of disease (Figure 4; Additional file 1: Table S7). These results highlight the need for health intervention strategies to be designed with differentiated approaches based on regional development levels.

**Table 2:** AAPC of ASMR and ASDR in countries with five SDI levels from 1990 to 2021.

Region	ASMR/100,000 persons (95% UI) (1990/2021)	AAPC of ASMR (95% CI)	ASDR/100,000 persons (95% UI) (1990/2021)	AAPC of ASDR (95% CI)
High SDI	15.79 (4.99, 27.01)/5.2 (1.6, 9.04)	−3.50 (−3.70–−3.30)	299.12 (104.22, 483.68)/125.19 (44.83, 199.95)	−2.80 (−2.90–−2.60)
Middle-high SDI	32.25 (10.21, 54.87)/16.45 (5.25, 28.31)	−2.10 (−2.40–−1.80)	606.86 (209.65, 974.04)/337.2 (119.19, 552.44)	−1.90 (−2.20–−1.50)
Middle SDI	16.51 (5.16, 28.69)/12.88 (4.08, 22.66)	−0.80 (−1.10–−0.50)	345.38 (120.84, 573.17)/275.17 (95.54, 458.71)	−0.70 (−0.90–−0.60)
Low-middle SDI	13.45 (4.26, 23.64)/11.61 (3.59, 20.28)	−0.40 (−0.50 - −0.40)	284.26 (97.09, 476.9)/248.6 (83.94, 421.23)	-0.40 (−0.60–−0.20)
Low SDI	11.68 (3.35, 21.05)/10.44 (3.13, 18.93)	−0.30 (−0.50–−0.20)	251.39 (78.86, 432.3)/222.55 (72.33, 383.74)	−0.40 (−0.40–−`0.30)

ASMR, Age-Standardized Mortality Rate; ASDR, age-standardized disability-adjusted life-year rate; AAPC, annual average percent change; SDI, Socio-Demographic Index; UI, uncertainty interval; CI, confidence interval.

### 3.5 Global trends by sex and age groups

In 2021, mortality was mostly concentrated among individuals aged 80–89 (Additional file 1: Figure S4A). Males under 80 showed higher mortality than females, but the trend reversed in those 80 and older (Additional file 1: Figure S5A). The mortality rate among females increased with age compared with the trend increased only up to the 90–94 age group in males (Additional file 1: Figure S6A). The highest DALYs burden can be seen in the 70–74 age group (Additional file 1: Figure S4B). Among patients under 80 years of age, the number of DALYs for males exceeded that of females. Additionally, males in the 60 to 74 age group maintained a relatively high level of DALYs (Additional file 1: Figure S5B). DALYs for females increased with age, while the peak for males occurred in the 90–94 age group (Additional file 1: Figure S6B). Overall, the age groups of 80–84 and 70–74 have the highest burden in the number of deaths and DALYs, respectively. Therefore, we believe that the age group of 70–84 may be the age group with the highest burden of stroke.

### 3.6 The age, period, and birth cohort

**Figure 5:**
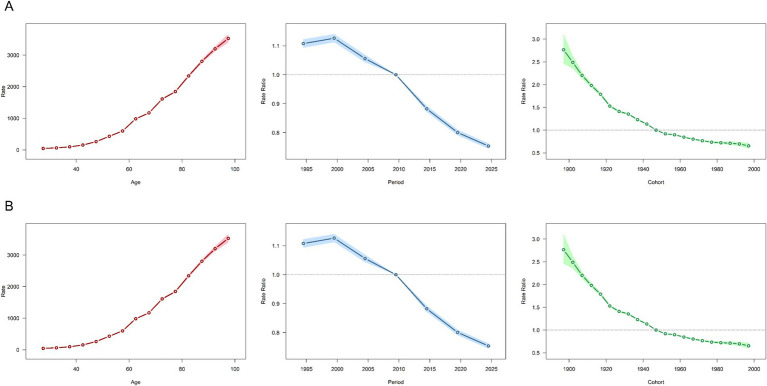
Analysis of Age, Period, and Birth cohort effects on Ischemic Stroke attributable to high LDL cholesterol globally, focusing on the ASMR and ASDR. **(A)** Analysis of the ASMR on a Global scale. **(B)** Analysis of the ASDR on a Global scale. ASMR Age-Standardized Mortality Rate, ASDR Age-Standardized Disability-Adjusted Life Year Rate.

Age-specific trends in stroke burden were consistent globally and across SDI levels (Figures 5A,B; Additional file 1: Figure S7). The risk of disease increases with age, particularly among individuals over 60, indicating a higher risk for the elderly. Middle-high SDI regions have higher overall ASMR and ASDR across all age groups, while high SDI regions show the lowest overall burden.

Period effects revealed a global reduction in mortality and DALYs after 2000, mirrored in middle-high SDI regions (Figures 5A,B). In contrast, other SDI regions have maintained a continuous decline since 1995, with the most significant reductions occurring in high SDI regions (Additional file 1: Figure S7). However, the decline in mid-SDI, lower mid-SDI, and low-SDI regions was minimal, suggesting limited efficacy of lipid-related interventions for ischemic stroke in these settings (Additional file 1: Figure S7).

Cohort effects showed declining risks in successive birth cohorts globally, aligning with SDI-specific trends. However, reductions were marginal in middle-, low-middle-, and low-SDI regions, underscoring persistent gaps in control measures, potentially linked to healthcare resource disparities (Additional file 1: Figure S7).

### 3.7 Cross-country social inequality analysis

**Figure 6:**
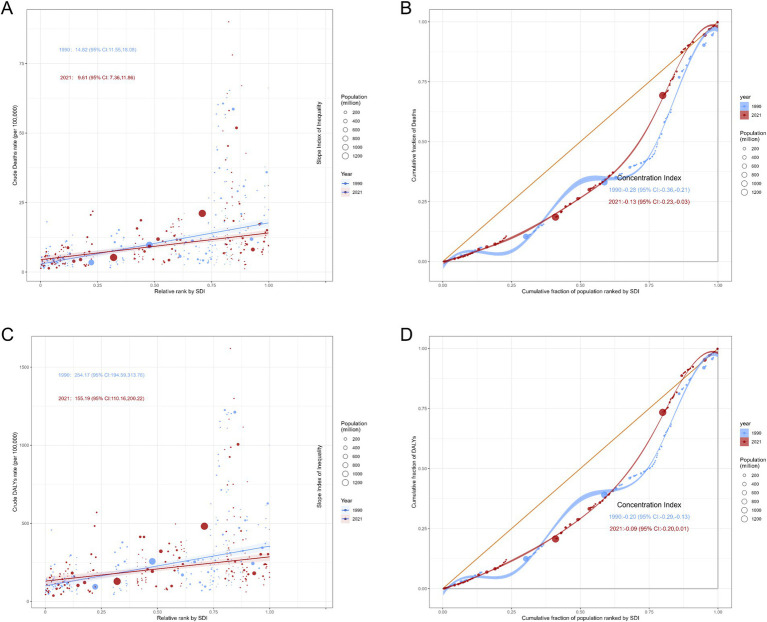
Slope Index of Inequality curves and Concentration Index curves for deaths and DALYs due to ischemic stroke attributable to high LDL cholesterol, 1990 and 2021. **(A)** Slope Index of Inequality curves for crude deaths rate of ischemic stroke. **(B)** Concentration Index curves for Deaths of ischemic stroke. **(C)** Slope Index of Inequality curves for crude DALYs rate of ischemic stroke. **(D)** Concentration Index curves for DALYs of ischemic stroke. DALYs Disability-Adjusted Life Years.

The global burden of ischemic stroke due to high LDL-C shows significant inequalities based on SDI levels. Key findings include: (1) The burden predominantly affects countries and regions with middle to high SDI levels. (2) The gap in burden across different SDI levels was progressively decreasing (Figure 6A,C; Additional file 1: Table S8).

The Slope Index of Inequality for crude death rates per 100,000 decreased from 14.82 (95% CI: 11.55, 18.08) in 1990 to 9.61 (95% CI: 7.36, 11.86) in 2021, while the Concentration Index declined from −0.28 (95% CI: −0.36, −0.21) to −0.13 (95% CI: −0.23, −0.03). DALYs exhibited a similar trend, with both the Slope Index of Inequality [254.17 (95% CI: 194.59, 313.76) vs. 155.19 (95% CI: 110.16, 200.22)] and Concentration Index [−0.20 (95% CI: −0.29, −0.16) vs. −0.09 (95% CI: −0.20, 0.01)] demonstrating varying degrees of decline (Figure 6B,D; Additional file 1: Table S8). The downward trend was more pronounced in females (Additional file 1: Table S8, Figure S8).

### 3.8 Frontier analysis

**Figure 7:**
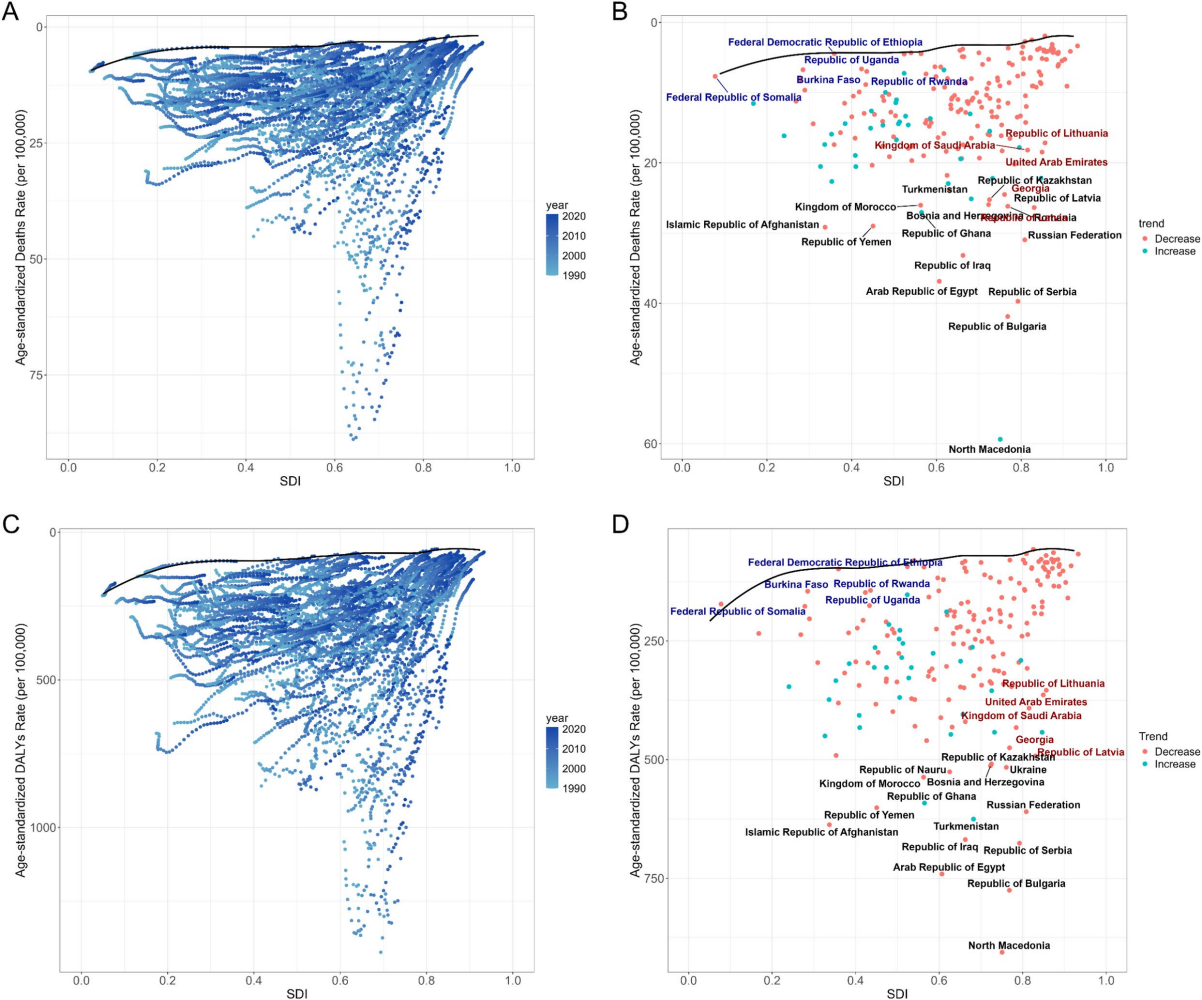
Frontier analysis based on age-standardized rates of deaths and DALYs for ischemic stroke attributable to high LDL cholesterol and the SDI over the decades (1990–2021), with a specific focus on 2021. **(A,C)** Illustrate the frontier analysis based on ASMR, ASDR, and SDI from 1990 to 2021. The color gradient transitions from light blue (1990) to dark blue (2021), with the black solid line delineating the boundary. **(B,D)** Present the frontier analysis for 2021 based on ASMR, ASDR, and SDI. The black solid line marks the boundary, while the dots represent countries and regions. The 15 countries and regions with the largest effective differences are highlighted in black. Countries with low SDI (<0.466) and lower effective differences are marked in blue, whereas those with high SDI (>0.810) and relatively high effective differences are marked in red. The red dots indicate a decrease in ASMR or ASDR, while blue dots signify an increase in ASMR or ASDR from 1990 to 2021. DALYs Disability-Adjusted Life Years, ASMR Age-Standardized Mortality Rate, ASDR Age-Standardized Disability-Adjusted Life Year Rate, SDI Socio-Demographic Index.

This study uses data from 1990 to 2021 to conduct a frontier analysis based on ASMR, ASDR, and SDI to explore the potential for reducing disease burden (Figure 7; Additional file 1: Tables S9 and S10).

From the perspective of ASMR, the top five countries with the greatest potential for improving disease burden are North Macedonia, Bulgaria, Serbia, Egypt, and Iraq, with effective differences ranging from 56.15 to 29.97 (Figure 7; Additional file 1: Table S9). In terms of ASDR, the rankings are similar to those of ASMR, with North Macedonia, Bulgaria, Egypt, Serbia, and Iraq also in the top five, showing effective difference ranges from 835.43 to 598.14 (Figure 7; Additional file 1: Table S10).

And we analyzed health interventions taken in countries with similar SDI levels. In low SDI countries (<0.466), Somalia, Ethiopia, Burkina Faso, Rwanda, and Uganda demonstrated better outcomes in managing the burden of disease compared to other countries (Figures 7B,D; Additional file 1: Tables S9 and S10). In high SDI countries (>0.810), Latvia, Georgia, Saudi Arabia, Lithuania and United Arab Emirates showed larger effective differences in both ASMR and ASDR, highlighting substantial potential for improving disease burden management (Figures 7B,D; Additional file 1: Tables S9 and S10).

### 3.9 Forecast of disease burden for the next 15 years

**Figure 8:**
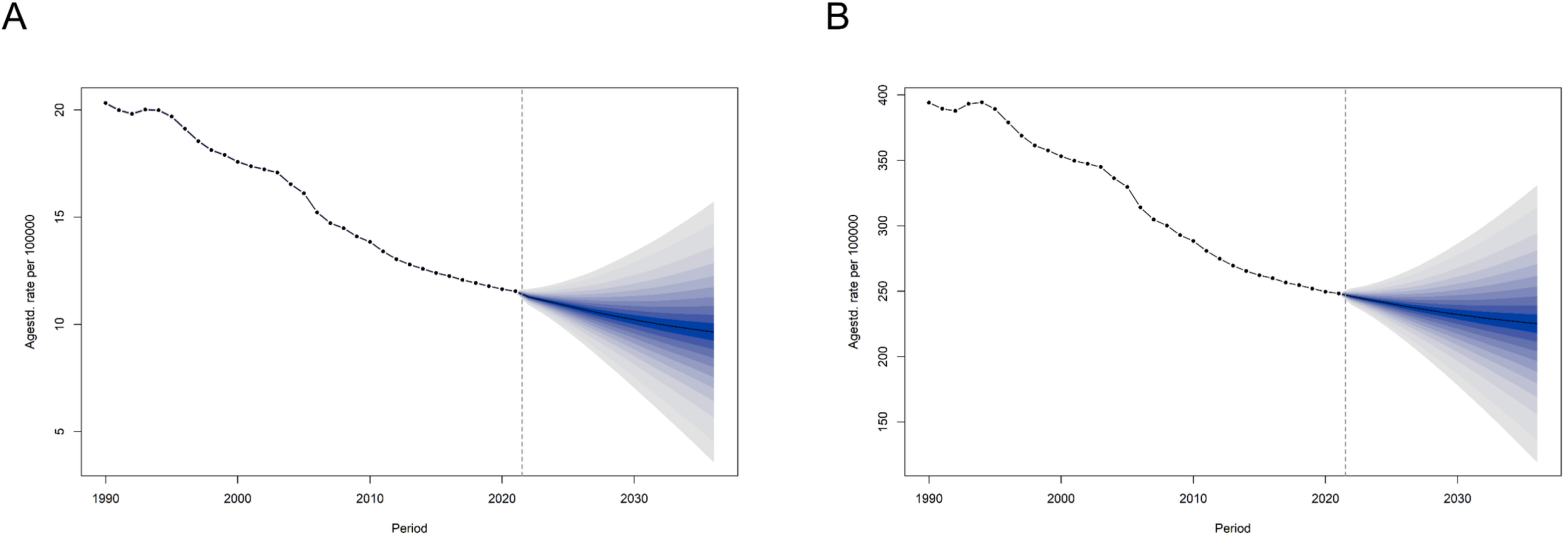
Prediction of the global disease burden for ischemic stroke attributable to high LDL cholesterol until 2036. **(A)** Prediction of the global disease burden for ischemic stroke attributable to high LDL cholesterol until 2036, based on ASMR. **(B)** Prediction of the global disease burden for ischemic stroke attributable to high LDL cholesterol until 2036, based on ASDR.

The Bayesian Age-Period-Cohort (BAPC) model was employed to forecast trends in ASMR and ASDR from 2022 to 2036. The model indicates that global ASMR and ASDR for ischemic stroke will generally decline during this period (Figures 8A,B; Additional file 1: Figures S10A,B).

Particular attention was given to China and India, two countries with large populations. The model predicts that the ASMR and ASDR trends in both countries will follow global patterns. However, China has a heavier overall burden than India (Additional file 1: Figures S9A,B,G,H). Analysis by age groups using our method reveals that the disease burden for individuals over 55 in both countries is gradually declining (Additional file 1: Figures S10I,J). However, in China, individuals over 55 exhibit a slight upward trend in disease burden (Additional file 1: Figures S10G,H). This may suggest that China is likely to face a higher burden of disease over the next 15 years among younger populations.

The model also forecasts the disease burden for the U.S. (a high-SDI region) and Sudan (a low-SDI region). In the U.S., the overall disease burden is on the rise, while in Sudan, both ASMR and ASDR are expected to remain stable or show a slight decline (Additional file 1: Figures S9C,D,E,F, S10C,D, E,F). These projections suggest that regions across different levels will continue to face significant burdens over the next 15 years, regardless of whether they are low or high SDI areas.

In the long term, the burden of ischemic stroke attributable to dyslipidemia in these countries is unlikely to see significant relief, and its severity is expected to persist.

## 4 Discussion

This study presents a thorough analysis of the global burden of ischemic stroke attributable to dyslipidemia, covering data from 1990 to 2021 as obtained from the GBD 2021 database. High LDL-C continues to be an important challenge for health systems globally. From 1990 to 2021, the number of deaths and DALYs due to ischemic stroke attributable to high LDL-C increased globally. However, the age-standardized mortality rate and DALY rate of ischemic stroke attributable to high LDL-C for both males and females showed a declining trend. Previous decomposition analyses have indicated that the increase in the burden of lipid-related ischemic stroke is primarily attributable to population aging and growth (22). The impact of aging on disease burden will be further elaborated in subsequent content. Excluding the effects of these two factors, the burden of disease actually decreased, which explains the inverse change between absolute numbers and age-standardized rates. These findings underscore the persistent role of LDL-C as a modifiable risk factor, and lipid-lowering treatments might be instrumental in reducing this global health burden (23). However, the differences of disease burden between regions with varying levels of development are significant, which highlights the need for region-specific public health strategies. Our study aims to further enhance the understanding of the stroke burden associated with high LDL-C and provide a basis for guiding public health policy.

From a regional perspective, middle and middle-high SDI regions experience a higher burden, both in absolute terms and in age-standardized measures. Conversely, high SDI areas tend to show the lowest ASMR and ASDR for ischemic stroke, aligning with previous studies (15). The reasons for the phenomenon of middle to high SDI regions suffering a higher burden are various; the insufficiencies in healthcare, stroke risk management, and secondary prevention measures may be part of the causes (24–26). In contrast, in high-SDI regions, we observed that the disease burden was the lowest in 2021 and has remained relatively stable in recent years. Upon investigating the reasons, we believe this is closely related to their well-developed healthcare infrastructure and advanced disease prevention concepts. For example, healthy diets and exercise (such as the Mediterranean diet) are based on a comprehensive understanding of stroke risk factors. This diet not only reduces low-density lipoprotein and cholesterol synthesis in the blood but also inhibits oxidative stress and inflammatory responses, playing an important role in the primary and secondary cardiovascular diseases prevention (27–29). The latest stroke primary prevention guidelines also highlight the Mediterranean diet and lipid management as important prevention strategies (30). Furthermore, previous studies have also confirmed that the preventive use of statins and the implementation of lifestyle changes can lead to a reduction in plasma cholesterol levels in the population, thereby lowering the risk of lipid-related diseases (31). In addition, the establishment of well-developed primary healthcare infrastructure, scientific stroke treatment methods, and effective stroke rehabilitation systems has created a favorable environment for reducing the stroke disease burden in these regions. We also observed that apart from the significant downward trends in ASMR and ASDR in middle-high and high SDI regions, other regions did not experience a noticeable decline in ASMR and ASDR. The reasons for this may include deficiencies in primary healthcare infrastructure, health insurance coverage, medical services, screening for high-risk patients, and health education in these areas. In low-SDI regions, limited access to statins and a lack of disease prevention awareness mean that only a small portion of the population can receive early pharmacological intervention (31, 32). This may partially explain why the disease burden in these areas has not significantly improved. Therefore, implementing more effective disease intervention measures in these low-development regions could be a crucial direction for reducing the global disease burden. Countries and regions with lower development levels can learn from the experiences of high SDI areas and their practical actions in controlling blood lipids. By increasing the coverage of primary healthcare services and enhancing awareness of the risk of stroke related to blood lipids, it may be possible to significantly reduce the burden of ischemic stroke caused by dyslipidemia. Medical assistance from high-development regions can provide strong support for improving the disease burden in these areas, including funding, personnel support, and assistance in formulating healthcare policies.

We further explored the potential for improvement in disease burden across nations by analyzing the disease burden of different countries. China and India are the two countries with the highest absolute burden which have large populations. Through further analysis, we identified that North Macedonia, Bulgaria, Egypt, Serbia, and Iraq are the five countries with the greatest potential for improvement. The burden of disease and the potential for reducing it vary considerably between countries, even among nations with similar SDI levels. This study also quantified disparities across countries by employing the Socioeconomic Inequality Index (SII) and Concentration Index (CI) for ischemic stroke burden from 1990 to 2021. The data reveal that the global ASMR and ASDR of ischemic stroke have shown a downward trend, with middle-high SDI regions suffering the largest burden. The differences in disease burden between countries have reduced, and the reduction in transnational inequality is greater among females. Considering the changes in disease burden across different SDI regions, we believe that improvements in healthcare access and management of blood lipid levels in middle-high SDI regions have played a critical role in reducing disease burden disparities. Meanwhile, the disease burden in other regions has stabilized. These findings highlight the imbalance in blood lipid management and stroke prevention progress across different regions. Health disparities are not only a public health issue but also an important ethical concern. Every individual, regardless of their economic status, geographic location, or social background, should have equal rights to health. The existing health disparities reflect inequalities in resource allocation and access to health services, which not only exacerbate the disease burden but also pose challenges to social justice. To reduce global inequalities in stroke burden, the international community has a responsibility to promote equitable resource distribution. In resource-limited developing countries and regions, implementing targeted interventions such as improving basic healthcare infrastructure, providing essential medications, and offering health education can effectively reduce the risk of stroke caused by hyperlipidemia (33). Additionally, developed countries play a crucial role in sharing advanced medical knowledge and technology. Through international cooperation and technology transfer, developed countries can help developing nations enhance their healthcare capabilities, thereby promoting health equity on a global scale. In conclusion, addressing health disparities requires not only scientific and technological efforts but also ethical and social commitments. Only through global cooperation and resource sharing can we truly achieve health equity and reduce inequalities in disease burden.

Aging is a recognized risk factor for ischemic stroke. In previous decomposition analyses, aging has been identified as a significant factor contributing to the increase in the absolute burden of ischemic stroke (22). The study used an Age-Period-Cohort (APC) model to analyze age effects on the stroke burden due to high LDL-C. Through this model, we gained a clear understanding of the significant impact of age on disease burden. Aging contributes to arterial stiffening and endothelial dysfunction, which are major factors in atherosclerosis—a precursor to ischemic stroke. Previous studies have indicated that aging may accelerate atherosclerosis through the synergistic effects of IL-6 signaling, decreased vascular mitochondrial function, and impaired mitochondrial autophagy (34). Additionally, the prevalence of hyperlipidemia increases with advancing age, which raises the risk of atherosclerosis (35, 36). Research has shown that lipid-rich unstable plaques are more likely to lead to the occurrence of ischemic stroke (37). For elderly patients, we need to pay more attention to their blood lipid levels and take intervention measures timely (38). For example, establishing a community-based dynamic monitoring system for LDL-C in the elderly and conducting semi-annual lipid screenings for individuals over 60 years old would be beneficial. Additionally, considering the characteristics of the elderly population, such as decreased vascular elasticity and comorbidities, employing a multidisciplinary approach to select geriatric-friendly medication regimens or integrating multidisciplinary teams to carry out post-stroke rehabilitation for the elderly are also important measures to reduce the stroke burden in this population. The APC model also highlighted the influence of period and birth cohort factors, with a downward trend in burden suggesting that efforts to manage LDL-C are showing positive effects.

Our study shows that the absolute burden of disease in females is higher than in males. In youth, the absolute burden of ischemic stroke is greater in males, but this pattern reverses in middle and old age. This may be explained by the occurrence of menopause and the decline in estrogen levels in females. Previous studies have confirmed that estrogen has a protective effect against cell death caused by premenopausal stroke (39). Estrogen interacts with specific receptors and metabolic enzymes expressed in vascular smooth muscle and endothelial cells, influencing vascular reactivity through various pathways, including enhancing endothelial nitric oxide synthase function, promoting NO production, and increasing the effectiveness of endothelial-derived hyperpolarizing factor (EDHF) (40). Additionally, estrogen inhibits the formation of atherosclerotic plaques through multiple pathways, including inhibiting the proliferation of vascular smooth muscle cells (SMCs), reducing the accumulation and oxidation of lipoprotein (A), and preventing platelet thrombosis (41). Furthermore, compared to females, males have lower total production and release of NO, which may underlie gender differences in vascular function (42). Higher rates of smoking and alcohol consumption in males are significant factors contributing to dyslipidemia, which may further explain why males experience a heavier burden of lipid-related ischemic stroke at an earlier age (43).

The study also projected future trends in disease burden over the next 15 years. Overall, a decline in the global disease burden is anticipated. China and India have been selected for predictive analysis, two countries with large populations bases. Their overall trends are similar to the global trend. However, further analysis by age groups shows that the disease burden among young people may rise. To explore this further, we selected two countries with different levels of SDI for in-depth analysis. The results indicate that the burden of stroke due to dyslipidemia among individuals under 55 is increasing, particularly in high-SDI regions like the United States. This aligns with the current trend of youth suffering a higher incidence of clinical cases and corroborates previous research findings (44). Previous studies on the stroke burden among young people aged 15–39 from 1990 to 2019 have also indicated an upward trend in the burden of ischemic stroke in this age group (44). The research pointed out that the burden of ischemic stroke among young people is rapidly increasing in high-income regions such as North America, North Africa, the Middle East, and South Asia. The analysis suggests that, in addition to factors such as increased consumption of sugary beverages, high body mass index (BMI), and reduced physical activity, changes in lifestyle and dietary habits due to rapid social transformation, as well as increased air pollution, may be important contributing factors (45, 46). In regions with lower SDI, the lack of primary stroke prevention resources is a significant factor in the rising disease burden (47). Although the global burden of stroke across all age groups is decreasing, we must note the trend of the disease burden becoming more prevalent among young people. It is crucial to monitor and manage lipid levels in youth, raise awareness, and implement early interventions to curb the rising trend of ischemic stroke in younger populations. Governments and health organizations around the world can engage in collaborative research and data sharing to explore the relationship between high LDL-C and ischemic stroke. By working together, they can develop effective prevention and treatment strategies. Such international cooperation can also facilitate the exchange of best practices and successful experiences, thereby promoting the implementation of effective health interventions on a global scale. At the same time, the BAPC model is based on historical data for its projections, but it cannot fully predict the impact of future changes in health policies and unexpected public health events on disease burden. Therefore, we need to remain highly vigilant regarding the disease burden of lipid-related ischemic stroke, ensuring continuous monitoring and timely adjustments to intervention measures.

This study differs from previous research in the following ways: (1) it provides an analysis of disease burden across regions and examines cross-national inequalities; (2) it projects disease burden trends over the next 15 years, with a detailed breakdown by age group. All of this can provide a deeper understanding of the burden of stroke caused by dyslipidemia. This is significant for optimizing global resource allocation and guiding the formulation of public health policies. However, we must also note that the quality of data sources in various regions may affect the accuracy of our data. Particularly in some low-SDI regions, due to inadequate systems and infrastructure, national population-based ischemic stroke registries and mortality information systems are underdeveloped. The quality and availability of data cannot be guaranteed, and the actual disease burden in these regions may be heavier. Furthermore, the SDI indicator encompasses three dimensions: income, education, and fertility rate. It is impossible to conduct a unidimensional analysis, while education level plays an important role in controlling lipid levels. Additionally, some regions lack comprehensive data on the disease burden caused by low-density lipoprotein cholesterol (LDL-C), making it difficult to generate fully representative estimates. In addition, our analysis lacks data on ischemic stroke in individuals under 25 years old, which may lead to certain biases. Additionally, the association between high LDL-C and ischemic stroke may be confounded by other cardiovascular risk factors, particularly hypertension, diabetes, and smoking. These factors have been widely confirmed to be closely related to the pathophysiological mechanisms of atherosclerosis and may interact synergistically with high LDL-C.

In clinical practice, the coexistence of dyslipidemia and hypertension is very common (48). Studies have shown that hypertension and dyslipidemia promote the formation of atherosclerotic plaques through various complex pathophysiological mechanisms, including oxidative stress, pro-inflammatory responses, activation of the renin-angiotensin-aldosterone system (RAAS), and endothelial dysfunction, thereby increasing the risk of stroke (49–51). Diabetic patients often have small, dense LDL particles that are more likely to promote atherosclerosis (52, 53). Smoking not only directly damages endothelial cells but also induces platelet and macrophage adhesion, leading to pro-thrombotic and inflammatory responses, which can accelerate atherosclerosis and further increase the risk of stroke (54, 55). Although the GBD database attempts to exclude or control for these potential confounding factors through precise epidemiological modeling and multivariable analysis methods, some degree of error may still exist.

In conclusion, this study highlights the global burden of ischemic stroke associated with dyslipidemia, utilizing the updated GBD database. We recognize that the global burden and cross-national inequalities have decreased, but the burden on younger populations may increase in the future. With the acceleration of global aging, the burden of stroke attributable to dyslipidemia continues to face significant challenges. The findings emphasize the necessity of developing targeted strategies to meet the needs of different regions and age groups. Improving basic healthcare infrastructure, enhancing awareness of healthy lifestyles, and controlling lipid levels are key steps in alleviating the global burden of ischemic stroke caused by dyslipidemia.

## 5 Conclusion

In the past 30 years, the overall burden of ischemic stroke caused by low-density lipoprotein cholesterol (LDL-C) has been controlled, but the issue remains prominent in regions with medium to high Socio-Demographic Index (SDI). Predictions show that the risk in younger populations may continue to rise over the next 15 years, thus requiring differentiated strategies. In regions with medium to high SDI, regional LDL-C control targets should be set, and carotid ultrasound should be included in annual check-ups for young people. In regions with medium to low SDI, primary healthcare institutions should establish fast lipid testing networks, and drugs such as atorvastatin should be included in the essential medicines list. Furthermore, research on the gender-specific impact of disease burden should be strengthened, and targeted solutions should be developed. For example, healthcare professionals could formulate differentiated management strategies based on gender to improve prevention and treatment outcomes. By implementing precise measures (regional stratification, early screening, drug accessibility, and gender strategies), the disease burden can be reduced, with a focus on adolescents and high-risk populations.

## Data Availability

Data supporting the findings of this study are available from the Global Burden of Disease (GBD) database. The data can be accessed at https://vizhub.healthdata.org/gbd-results.
