# Supplementary material for: Systematic analysis of the burden of ischemic stroke attributable to high LDL-C from 1990 to 2021
Source: Front Neurol. 2025 Apr 4;16:1547714. doi: 10.3389/fneur.2025.1547714 (PMC12005992; doi:10.3389/fneur.2025.1547714)
Supplement: Supplementary file 1 [file Data_Sheet_1.PDF]

Table S1 The Number of Death and DALYs in 21 regions and global in 2021

Location	Death/1000（95%UI）			DALYs/1000（95%UI）		
	**Both**	**Male**	**Female**	**Both**	**Male**	**Female**
Global	936.19 (299.54,1614.29)	461.38 (150.64,786.28)	474.81 (148.89,836.96)	20977.42 (7409.04,34664.41)	11039.93 (3969.6,18176.49)	9937.49 (3435.54,16487.97)
Andean Latin America	2.54 (0.79,4.46)	1.21 (0.37,2.14)	1.32 (0.41,2.31)	55.26 (19.3,91.91)	27.26 (9.66,45.02)	28 (9.81,46.61)
Australasia	2.57 (0.74,4.61)	1.01 (0.29,1.8)	1.56 (0.44,2.8)	45.8 (14.77,77.59)	22.01 (7.31,36.52)	23.78 (7.52,40.1)
Caribbean	5.09 (1.53,8.84)	2.28 (0.71,3.94)	2.81 (0.84,5.05)	103.13 (35.5,173.67)	48.66 (16.92,81)	54.47 (18.41,93.25)
Central Asia	12.44 (3.99,21.25)	5.98 (2,10.03)	6.46 (2.02,11.26)	325.27 (120.21,520.36)	166.21 (62.79,263.15)	159.07 (58.69,257.08)
Central Europe	46.01 (14.12,78.74)	19.62 (6.28,32.9)	26.39 (7.82,46.27)	824.24 (273.81,1359.27)	402.7 (138.83,654.54)	421.55 (134.67,714.88)
Central Latin America	11.59 (3.54,20)	5.5 (1.66,9.32)	6.09 (1.85,10.61)	251.64 (90.33,404.39)	122.92 (43.4,197.7)	128.72 (47.01,206.5)
Central Sub-Saharan Africa	4.28 (1.26,8.12)	1.77 (0.51,3.35)	2.52 (0.76,5.04)	117.78 (39.33,207.19)	50.62 (16.36,91)	67.16 (23.47,124.46)
East Asia	307.72 (95.03,538.67)	174.07 (56.38,304.05)	133.65 (40.62,241.11)	7056.8 (2385.26,11732.69)	4050.43 (1418.65,6790.97)	3006.37 (1028.44,5086.21)
Eastern Europe	101.52 (34.05,169.99)	39.17 (13.66,63.69)	62.35 (19.16,107.34)	1990.87 (712.2,3176.87)	925.4 (359.02,1438.52)	1065.47 (361,1756.99)
Eastern Sub-Saharan Africa	11.06 (3.42,20.05)	5.22 (1.63,9.56)	5.84 (1.77,11.02)	307.16 (104.25,527.61)	146.3 (50.75,254.1)	160.85 (53.77,274.5)
High-income Asia Pacific	27.8 (7.89,50.62)	12.61 (3.57,23.03)	15.19 (4.2,27.99)	515.75 (163.86,881.92)	278.38 (90.52,469.76)	237.37 (73.08,414.59)
High-income North America	29.68 (8.32,53.27)	11.28 (3.49,20.01)	18.39 (5.09,33.52)	660.07 (221.38,1109.95)	295.79 (102.78,488.11)	364.27 (118.59,621.13)
North Africa and Middle East	73.26 (24.36,127.08)	35.01 (11.48,61.03)	38.25 (12.96,67.13)	1831.67 (694.03,2939.67)	907.57 (340.17,1461.63)	924.1 (355.42,1518.18)
Oceania	0.5 (0.16,0.9)	0.21 (0.07,0.4)	0.29 (0.09,0.5)	14.96 (5.64,25.18)	6.58 (2.32,11.58)	8.37 (3.18,13.68)
South Asia	100.98 (33.78,178.19)	50.45 (16.31,92.85)	50.52 (16.07,91.88)	2442.54 (896.34,4115.82)	1252.89 (435.15,2176.66)	1189.65 (423.14,2034.66)
Southeast Asia	85.87 (27.82,147.92)	43.67 (14.6,76.48)	42.2 (13.35,74.69)	2165.98 (777.56,3548.25)	1159.79 (414.38,1923.22)	1006.19 (356.94,1640.26)
Southern Latin America	5.26 (1.62,9.29)	2.4 (0.74,4.16)	2.86 (0.83,5.13)	107.81 (36.72,176.76)	54.7 (18.85,87.99)	53.11 (17.65,88.87)
Southern Sub-Saharan Africa	5.85 (1.78,10.49)	2.18 (0.71,3.81)	3.67 (1.1,6.57)	142.03 (48.86,236.8)	57.75 (20.93,94.95)	84.28 (27.97,141.35)
Tropical Latin America	21.24 (7.04,36.35)	10.88 (3.67,18.13)	10.36 (3.22,17.97)	434.79 (161.22,693.31)	238.17 (89.49,379.18)	196.61 (70.03,322.08)
Western Europe	59.19 (17.11,104.8)	23.75 (7.09,41.84)	35.44 (10.16,63.63)	999.84 (320.74,1695.28)	472.51 (155.1,786.71)	527.33 (164.22,905.65)
Western Sub-Saharan Africa	21.73 (6.88,37.37)	13.1 (4.33,22.26)	8.63 (2.54,15.76)	584.04 (208.33,974.03)	353.28 (127.73,577.85)	230.76 (79.55,378.06)

DALYs Disability-Adjusted Life Years, UI uncertainty interval

Table S2 ASMR, and ASDR in 21 regions and global in 2021

Location	ASMR/100,000 persons（95%UI）			ASDR/100,000 persons（95%UI）		
	Both	Male	Female	Both	Male	Female
Global	11.38 (3.62,19.65)	12.81 (4.09,22.14)	10.12 (3.18,17.81)	246.42 (86.47,406.28)	280.47 (98.55,469.59)	215.42 (75.19,355.77)
Andean Latin America	4.54 (1.41,7.98)	4.7 (1.42,8.35)	4.36 (1.35,7.63)	94.36 (32.48,157.4)	98.47 (33.8,164.94)	90.34 (31.26,151.7)
Australasia	3.9 (1.13,6.95)	3.82 (1.1,6.78)	3.86 (1.11,6.91)	80.37 (27.14,132.37)	88.42 (30.16,144.26)	72.12 (24.2,120.73)
Caribbean	9.31 (2.81,16.18)	9.32 (2.9,16.15)	9.23 (2.77,16.61)	191.26 (66.14,322.08)	194.57 (67.15,324.3)	187.67 (63.93,318.86)
Central Asia	18.33 (5.66,32.17)	21.28 (6.57,37.34)	16.12 (4.94,28.51)	409.88 (144.76,672.31)	475.63 (167.33,783.58)	357.96 (128.78,590.27)
Central Europe	19.09 (5.94,32.5)	21.72 (6.92,36.51)	16.89 (5.11,29.39)	361.96 (124.88,587.3)	433.18 (150.99,701.3)	302.08 (102.44,499.91)
Central Latin America	4.95 (1.5,8.61)	5.26 (1.57,8.99)	4.66 (1.4,8.15)	102.57 (36.07,166.47)	109.72 (37.13,177.46)	96.26 (34.84,155.5)
Central Sub-Saharan Africa	12.13 (3.23,23.58)	11.35 (3.03,22.22)	12.47 (3.34,25.47)	248.41 (76.38,453.71)	232.29 (67,439.84)	257.69 (84.34,503.21)
East Asia	15.69 (4.77,27.55)	20.49 (6.58,36.16)	12.23 (3.7,22.15)	333.23 (112.38,560.93)	411.77 (141.42,695.35)	268 (91.97,452.86)
Eastern Europe	28.15 (9.5,46.96)	32.34 (11.13,53.31)	24.8 (7.87,42.34)	565.86 (206.45,894.67)	704.08 (266.76,1106.72)	463.06 (165.37,741.86)
Eastern Sub-Saharan Africa	9.06 (2.65,16.7)	8.99 (2.58,16.74)	9.03 (2.55,17.27)	196.28 (62.89,346.62)	194.6 (62.78,347.61)	196.37 (62.1,345.05)
High-income Asia Pacific	4.04 (1.19,7.22)	5.23 (1.57,9.44)	3.06 (0.88,5.55)	103.65 (36.02,167.03)	135.85 (46.38,220.91)	75.51 (26.71,124.06)
High-income North America	4 (1.16,7.12)	3.85 (1.19,6.82)	4 (1.13,7.21)	102.78 (35.97,168.01)	104.88 (37.3,171.15)	99.75 (34.55,163.28)
North Africa and Middle East	19.78 (6.25,35.16)	18.47 (5.78,32.91)	21.03 (6.78,37.69)	411.22 (145.05,685.05)	396.69 (137.76,671.25)	425.42 (154.08,720.59)
Oceania	9.86 (2.91,18.33)	8.11 (2.21,15.96)	11.58 (3.59,21.26)	218.38 (72.49,383.36)	185.41 (58.2,336.33)	252.09 (86.52,428.79)
South Asia	8.21 (2.6,14.56)	8.57 (2.67,15.8)	7.88 (2.44,14.36)	172.3 (61.02,293.31)	181.2 (60.5,322.06)	163.87 (55.86,283.62)
Southeast Asia	16.13 (5.01,28.52)	17.9 (5.47,31.7)	14.48 (4.32,25.82)	347.11 (119.88,576.98)	395.26 (133.67,674.33)	303.58 (105.58,502.42)
Southern Latin America	5.7 (1.76,10.03)	6.65 (2.05,11.57)	4.95 (1.49,8.81)	121.56 (42.26,197.7)	145.33 (49.91,234.43)	102.29 (35.4,167.99)
Southern Sub-Saharan Africa	13.31 (3.96,24.37)	12.18 (3.65,21.97)	13.58 (4.02,24.77)	269.14 (85.73,470.81)	255.91 (84.34,441.04)	272.2 (86.53,469.42)

Tropical Latin America	8.67 (2.85,14.92)	10.49 (3.49,17.8)	7.23 (2.26,12.54)	171.85 (62.72,276.09)	212.48 (77.08,342.62)	138.6 (49.49,226.94)
Western Europe	4.71 (1.4,8.23)	5.1 (1.58,8.91)	4.3 (1.27,7.61)	96.36 (33.06,158.94)	110.67 (38.04,179.66)	83.2 (28.2,137.08)
Western Sub-Saharan Africa	14.65 (4.43,25.39)	17.78 (5.56,30.92)	11.88 (3.3,21.74)	310.14 (102.31,526.16)	384.39 (129.71,647.05)	244 (78.7,414.27)

ASMR Age-Standardized Disability-Adjusted Life Year Rate, ASDR Age-Standardized Disability-Adjusted Life Year Rate, UI uncertainty interval

Table S3 AAPC of ASMR and ASDR for in 21 regions and global from 1990 to 2021

Location	AAPC of ASMR (95% CI)			AAPC of ASDR (95% CI)		
	Both	Male	Female	Both	Male	Female
Global	-1.80 (-2.00 - -1.60)	-1.50 (-1.70 - -1.30)	-2.10 (-2.30 - -1.80)	-1.50 (-1.70 - -1.30)	-1.20 (-1.40 - -1.00)	-1.70 (-1.90 - -1.50)
Andean Latin America	-1.80 (-2.30 - -1.20)	-1.60 (-2.10 - -1.10)	-1.90 (-2.50 - -1.30)	-1.70 (-2.20 - -1.30)	-1.60 (-2.00 - -1.20)	-1.80 (-2.30 - -1.40)
Australasia	-3.90 (-4.20 - -3.70)	-4.10 (-4.40 - -3.80)	-3.70 (-4.00 - -3.50)	-3.30 (-3.50 - -3.10)	-3.40 (-3.60 - -3.20)	-3.30 (-3.50 - -3.10)
Caribbean	-1.10 (-1.50 - -0.70)	-0.80 (-0.90 - -0.70)	-1.40 (-1.70 - -1.00)	-0.90 (-1.20 - -0.50)	-0.60 (-0.80 - -0.40)	-1.10 (-1.30 - -0.80)
Central Asia	-0.50 (-0.80 - -0.20)	-0.40 (-0.90 - 0.10)	-0.50 (-1.20 - 0.20)	-0.50 (-0.80 - -0.30)	-0.50 (-1.00 - -0.10)	-0.60 (-0.90 - -0.40)
Central Europe	-2.50 (-2.70 - -2.30)	-2.50 (-2.70 - -2.20)	-2.60 (-2.80 - -2.50)	-2.40 (-2.60 - -2.20)	-2.30 (-2.50 - -2.10)	-2.50 (-2.70 - -2.30)
Central Latin America	-2.20 (-2.50 - -1.80)	-1.80 (-2.00 - -1.60)	-2.50 (-2.90 - -2.10)	-2.00 (-2.30 - -1.70)	-1.70 (-1.90 - -1.40)	-2.30 (-2.60 - -1.90)
Central Sub-Saharan Africa	-0.30 (-0.30 - -0.20)	-0.20 (-0.30 - -0.10)	-0.40 (-0.50 - -0.30)	-0.40 (-0.40 - -0.30)	-0.30 (-0.40 - -0.30)	-0.50 (-0.60 - -0.40)
East Asia	-0.50 (-0.70 - -0.20)	-0.10 (-0.40 - 0.20)	-0.90 (-1.20 - -0.60)	-0.50 (-0.70 - -0.30)	-0.20 (-0.40 - 0.00)	-0.80 (-1.10 - -0.60)
Eastern Europe	-1.90 (-2.40 - -1.40)	-1.80 (-2.30 - -1.20)	-2.10 (-2.80 - -1.50)	-1.70 (-2.30 - -1.10)	-1.50 (-2.10 - -0.90)	-2.00 (-2.60 - -1.30)
Eastern Sub-Saharan Africa	-0.10 (-0.20 - -0.00)	0.10 (-0.00 - 0.20)	-0.30 (-0.40 - -0.20)	-0.10 (-0.20 - -0.10)	0.10 (0.00 - 0.20)	-0.40 (-0.50 - -0.30)
High-income Asia Pacific	-4.40 (-4.90 - -4.00)	-4.10 (-4.50 - -3.70)	-4.90 (-5.40 - -4.40)	-3.50 (-3.70 - -3.20)	-3.20 (-3.40 - -3.00)	-3.90 (-4.20 - -3.60)
High-income North America	-2.60 (-3.00 - -2.10)	-2.90 (-3.40 - -2.40)	-2.30 (-2.70 - -1.80)	-1.90 (-2.20 - -1.60)	-2.20 (-2.60 - -1.90)	-1.70 (-2.00 - -1.50)
North Africa and Middle East	-1.10 (-1.30 - -0.90)	-1.00 (-1.20 - -0.80)	-1.10 (-1.40 - -0.80)	-1.10 (-1.10 - -1.00)	-1.00 (-1.10 - -0.80)	-1.10 (-1.30 - -1.00)
Oceania	-0.60 (-0.70 - -0.60)	-0.60 (-0.70 - -0.50)	-0.60 (-0.60 - -0.50)	-0.60 (-0.60 - -0.50)	-0.60 (-0.70 - -0.50)	-0.60 (-0.70 - -0.50)
South Asia	-0.30 (-0.80 - 0.20)	-0.10 (-0.30 - 0.10)	-0.60 (-1.20 - -0.00)	-0.40 (-0.60 - -0.20)	-0.20 (-0.50 - 0.00)	-0.60 (-1.00 - -0.30)

Southeast Asia	-0.20 (-0.30 - -0.10)	0.00 (-0.10 - 0.10)	-0.40 (-0.50 - -0.30)	-0.20 (-0.30 - -0.10)	0.00 (-0.10 - 0.10)	-0.50 (-0.60 - -0.40)
Southern Latin America	-3.20 (-3.50 - -2.80)	-3.00 (-3.30 - -2.60)	-3.30 (-3.60 - -3.00)	-2.80 (-3.00 - -2.60)	-2.70 (-3.10 - -2.30)	-2.80 (-3.10 - -2.60)
Southern Sub-Saharan Africa	0.90 (0.50 - 1.30)	0.50 (0.20 - 0.90)	1.10 (0.50 - 1.60)	0.50 (0.10 - 0.90)	0.30 (-0.00 - 0.70)	0.50 (0.20 - 0.80)
Tropical Latin America	-3.00 (-3.40 - -2.70)	-2.90 (-3.30 - -2.40)	-3.20 (-3.40 - -3.00)	-3.00 (-3.40 - -2.60)	-2.90 (-3.30 - -2.50)	-3.00 (-3.30 - -2.80)
Western Europe	-4.40 (-4.50 - -4.20)	-4.50 (-4.70 - -4.40)	-4.40 (-4.70 - -4.00)	-3.80 (-4.00 - -3.50)	-3.80 (-4.00 - -3.70)	-3.80 (-4.00 - -3.60)
Western Sub-Saharan Africa	-0.30 (-0.30 - -0.20)	-0.20 (-0.30 - -0.10)	-0.30 (-0.40 - -0.20)	-0.30 (-0.30 - -0.30)	-0.20 (-0.20 - -0.10)	-0.30 (-0.40 - -0.30)

ASMR Age-Standardized Disability-Adjusted Life Year Rate, ASDR Age-Standardized Disability-Adjusted Life Year Rate, AAPC annual average percent change, CI confidence interval.

Table S4 The Number of Deaths and DALYs in 204 countries in 2021

Location	Death/1000（95%UI）			DALYs/1000（95%UI）		
	Both	Male	Female	Both	Male	Female
American Samoa	0 (0,0.01)	0 (0,0)	0 (0,0)	0.09 (0.04,0.15)	0.04 (0.02,0.07)	0.05 (0.02,0.08)
Antigua and Barbuda	0.01 (0,0.01)	0 (0,0.01)	0 (0,0.01)	0.16 (0.05,0.26)	0.07 (0.02,0.12)	0.08 (0.03,0.14)
Arab Republic of Egypt	16.56 (5.66,27.5)	8.11 (2.72,14.57)	8.46 (2.96,14.41)	436.76 (168.08,720.25)	220.77 (83.6,378.13)	215.99 (81.76,350.91)
Argentine Republic	3.09 (0.95,5.45)	1.43 (0.44,2.46)	1.67 (0.5,2.99)	66.34 (22.52,108.37)	33.79 (11.55,54.62)	32.55 (10.81,54.31)
Australia	2.08 (0.59,3.72)	0.82 (0.23,1.47)	1.26 (0.35,2.26)	37.48 (12.05,63.5)	18.3 (6.06,30.39)	19.18 (6.05,32.53)
Barbados	0.06 (0.02,0.1)	0.02 (0.01,0.04)	0.03 (0.01,0.06)	0.99 (0.32,1.69)	0.43 (0.14,0.73)	0.56 (0.18,0.96)
Belize	0.02 (0,0.03)	0.01 (0,0.01)	0.01 (0,0.01)	0.34 (0.12,0.56)	0.17 (0.06,0.28)	0.17 (0.06,0.28)
Bermuda	0.01 (0,0.01)	0 (0,0.01)	0 (0,0.01)	0.14 (0.04,0.23)	0.07 (0.02,0.12)	0.07 (0.02,0.12)
Bolivarian Republic of Venezuela	1.7 (0.5,3.03)	0.75 (0.22,1.35)	0.95 (0.29,1.75)	33.32 (11.09,56.42)	15.46 (5.06,26.9)	17.86 (6.08,30.99)
Bosnia and Herzegovina	1.68 (0.56,2.91)	0.71 (0.24,1.24)	0.97 (0.31,1.72)	32.12 (11.3,53.15)	15.16 (5.49,25.3)	16.96 (5.67,28.84)
Brunei Darussalam	0.02 (0.01,0.04)	0.01 (0,0.02)	0.01 (0,0.02)	0.63 (0.23,1.01)	0.34 (0.13,0.54)	0.29 (0.11,0.47)
Burkina Faso	0.48 (0.14,0.96)	0.31 (0.09,0.62)	0.17 (0.05,0.34)	13 (4.13,24.42)	8.28 (2.5,16.07)	4.72 (1.57,8.54)
Canada	2.71 (0.73,4.83)	1.05 (0.31,1.87)	1.65 (0.44,2.96)	60.56 (19.88,100.91)	28.74 (9.83,47.31)	31.82 (10.05,54.27)
Central African Republic	0.19 (0.05,0.35)	0.08 (0.02,0.16)	0.11 (0.03,0.22)	5.53 (1.79,9.96)	2.51 (0.81,4.67)	3.02 (0.93,5.55)
Commonwealth of Dominica	0.01 (0,0.02)	0 (0,0.01)	0.01 (0,0.01)	0.17 (0.05,0.29)	0.07 (0.03,0.12)	0.09 (0.03,0.17)

Commonwealth of the Bahamas	0.03 (0.01,0.04)	0.01 (0,0.02)	0.01 (0,0.03)	0.55 (0.2,0.94)	0.23 (0.08,0.39)	0.32 (0.11,0.54)
Cook Islands	0 (0,0)	0 (0,0)	0 (0,0)	0.04 (0.01,0.07)	0.02 (0.01,0.03)	0.02 (0.01,0.03)
Czech Republic	2.14 (0.66,3.72)	0.92 (0.3,1.56)	1.22 (0.37,2.16)	41.94 (14.02,68.57)	20.91 (7.23,33.95)	21.03 (6.77,35.48)
Democratic People's Republic of Korea	5.77 (1.94,10.12)	2.55 (0.86,4.42)	3.22 (1,6.08)	150.41 (54.7,252.34)	73.82 (26.35,123.55)	76.59 (27.34,132.21)
Democratic Republic of Sao Tome and Principe	0.01 (0,0.03)	0.01 (0,0.01)	0.01 (0,0.01)	0.41 (0.14,0.66)	0.21 (0.07,0.36)	0.19 (0.07,0.32)
Democratic Republic of the Congo	2.71 (0.79,5.43)	1.09 (0.27,2.27)	1.63 (0.45,3.48)	73.16 (23.46,135.28)	30.98 (9.45,60.42)	42.17 (13.77,83.56)
Democratic Republic of Timor-Leste	0.1 (0.03,0.19)	0.05 (0.01,0.1)	0.05 (0.02,0.1)	2.46 (0.84,4.26)	1.25 (0.4,2.33)	1.21 (0.41,2.17)
Democratic Socialist Republic of Sri Lanka	4.26 (1.29,7.48)	1.86 (0.57,3.34)	2.4 (0.71,4.18)	84.68 (29.81,141.4)	39.91 (14.11,68.34)	44.77 (15.78,74.69)
Dominican Republic	0.88 (0.28,1.65)	0.42 (0.14,0.76)	0.46 (0.14,0.82)	19.48 (7.06,33.34)	9.93 (3.43,17.41)	9.55 (3.4,16.12)
Eastern Republic of Uruguay	0.64 (0.19,1.14)	0.25 (0.08,0.44)	0.39 (0.11,0.7)	10.9 (3.59,18.17)	5.01 (1.74,8.13)	5.89 (1.85,10.02)
Federal Democratic Republic of Ethiopia	1.49 (0.43,2.82)	0.64 (0.16,1.35)	0.85 (0.25,1.64)	40.83 (13.41,73.3)	16.84 (5.04,33.08)	23.99 (8.13,43.14)
Federal Democratic Republic of Nepal	1.67 (0.45,3.16)	0.85 (0.25,1.71)	0.82 (0.22,1.54)	38.44 (11.49,71)	19.64 (6.23,37.81)	18.8 (5.97,34.29)
Federal Republic of Germany	12.86 (3.83,22.67)	5.66 (1.8,9.67)	7.2 (2.1,13.1)	258.41 (85.11,427.46)	128.22 (43.39,210.43)	130.19 (41.73,218.96)
Federal Republic of Nigeria	9.03 (2.97,15.26)	5.68 (1.86,10.3)	3.35 (0.92,6.12)	237.56 (85.79,379.02)	146.25 (53.41,248.81)	91.3 (30.58,150.44)
Federal Republic of Somalia	0.29 (0.08,0.56)	0.11 (0.03,0.26)	0.18 (0.05,0.37)	9.63 (3.03,17.1)	3.85 (1.21,8.06)	5.78 (1.86,10.93)
Federated States of Micronesia	0.01 (0,0.01)	0 (0,0.01)	0 (0,0.01)	0.23 (0.09,0.38)	0.12 (0.04,0.2)	0.12 (0.04,0.2)
Federative Republic of Brazil	20.66 (6.85,35.3)	10.59 (3.58,17.55)	10.08 (3.14,17.45)	423.84 (156.92,675.72)	232.12 (87.05,368.8)	191.72 (68.35,314.94)
French Republic	8.08 (2.32,14.23)	3.17 (0.97,5.52)	4.91 (1.39,8.8)	132.79 (42.1,226.82)	63.77 (21.42,104.59)	69.02 (20.7,119.89)
Gabonese Republic	0.09 (0.03,0.16)	0.04 (0.01,0.07)	0.05 (0.01,0.09)	2.38 (0.78,3.96)	1.12 (0.37,1.97)	1.26 (0.43,2.2)
Georgia	1.46 (0.43,2.61)	0.62 (0.19,1.11)	0.84 (0.23,1.5)	26.91 (8.88,45.51)	12.83 (4.4,21.23)	14.08 (4.52,24.28)
Grand Duchy of Luxembourg	0.06 (0.02,0.1)	0.03 (0.01,0.04)	0.03 (0.01,0.06)	0.9 (0.3,1.51)	0.46 (0.16,0.76)	0.44 (0.14,0.76)
Greenland	0 (0,0.01)	0 (0,0)	0 (0,0)	0.1 (0.04,0.17)	0.06 (0.02,0.1)	0.04 (0.01,0.07)
Grenada	0.01 (0,0.02)	0 (0,0.01)	0.01 (0,0.01)	0.24 (0.08,0.4)	0.11 (0.04,0.18)	0.13 (0.04,0.22)
Guam	0.01 (0,0.01)	0 (0,0.01)	0 (0,0.01)	0.29 (0.11,0.46)	0.14 (0.06,0.23)	0.14 (0.06,0.24)
Hashemite Kingdom of Jordan	0.85	0.39	0.46 (0.15,0.8)	24.05	12.12	11.93

	(0.28,1.46)	(0.14,0.66)		(9.17,38.17)	(4.69,19.54)	(4.46,19.16)
Hellenic Republic	2.75 (0.75,5.03)	1.01 (0.28,1.84)	1.74 (0.47,3.16)	38.17 (11.56,66.8)	16.15 (5.11,28.26)	22.02 (6.28,39.27)
Hungary	2.89 (0.95,4.84)	1.27 (0.45,2.09)	1.62 (0.5,2.77)	57.53 (20.53,91.57)	29.03 (10.93,45.7)	28.5 (9.51,46.8)
Independent State of Papua New Guinea	0.32 (0.09,0.59)	0.12 (0.04,0.26)	0.19 (0.06,0.34)	9.27 (3.29,16.31)	3.84 (1.31,7.28)	5.43 (2.01,9.27)
Independent State of Samoa	0.01 (0,0.02)	0 (0,0.01)	0.01 (0,0.01)	0.31 (0.11,0.53)	0.15 (0.06,0.25)	0.16 (0.06,0.29)
Ireland	0.31 (0.09,0.58)	0.14 (0.04,0.25)	0.18 (0.05,0.33)	5.23 (1.71,9.16)	2.55 (0.86,4.47)	2.68 (0.85,4.64)
Islamic Republic of Afghanistan	2.28 (0.78,4.03)	1.04 (0.34,1.96)	1.23 (0.42,2.37)	65.26 (23.92,111.61)	29.03 (10.56,52.15)	36.23 (13.43,64.55)
Islamic Republic of Iran	9.94 (3.23,16.71)	4.89 (1.59,8.3)	5.05 (1.63,8.51)	238.27 (89.1,372.99)	122.93 (46.85,192.48)	115.34 (43.03,181.71)
Islamic Republic of Mauritania	0.28 (0.09,0.55)	0.16 (0.05,0.3)	0.13 (0.04,0.27)	6.94 (2.34,12.44)	3.75 (1.29,6.76)	3.2 (1.05,6.03)
Islamic Republic of Pakistan	10.59 (3.68,19.32)	5.17 (1.7,9.79)	5.42 (1.9,10.21)	285.33 (108.97,487.4)	140.42 (51.32,253.26)	144.91 (58.77,256.29)
Jamaica	0.38 (0.11,0.67)	0.15 (0.04,0.27)	0.22 (0.06,0.4)	6.26 (1.99,10.8)	2.74 (0.88,4.77)	3.52 (1.16,5.99)
Japan	21.97 (6.27,39.77)	10 (2.77,18.12)	11.97 (3.21,22.2)	385.65 (119.89,668.86)	208.3 (66.44,353.42)	177.35 (52.85,310.8)
Kingdom of Bahrain	0.07 (0.03,0.12)	0.04 (0.01,0.06)	0.03 (0.01,0.06)	2.06 (0.83,3.16)	1.16 (0.47,1.75)	0.9 (0.35,1.41)
Kingdom of Belgium	1.27 (0.37,2.27)	0.5 (0.15,0.89)	0.77 (0.22,1.38)	21.83 (6.96,37.21)	10.08 (3.25,17.11)	11.76 (3.69,20.15)
Kingdom of Bhutan	0.06 (0.02,0.1)	0.03 (0.01,0.05)	0.03 (0.01,0.05)	1.23 (0.41,2.1)	0.64 (0.21,1.12)	0.59 (0.19,1)
Kingdom of Cambodia	1.51 (0.46,2.73)	0.6 (0.19,1.06)	0.91 (0.26,1.67)	35.6 (11.87,62.52)	14.93 (5.03,26.93)	20.67 (6.88,36.05)
Kingdom of Denmark	0.78 (0.22,1.35)	0.34 (0.1,0.59)	0.44 (0.12,0.77)	13.05 (4.23,21.85)	6.49 (2.13,10.72)	6.57 (2.08,11.23)
Kingdom of Eswatini	0.05 (0.01,0.1)	0.02 (0,0.04)	0.03 (0.01,0.06)	1.34 (0.41,2.46)	0.54 (0.16,1.01)	0.8 (0.24,1.5)
Kingdom of Lesotho	0.11 (0.03,0.21)	0.04 (0.01,0.07)	0.08 (0.02,0.15)	2.73 (0.83,5.02)	1 (0.31,1.92)	1.73 (0.5,3.23)
Kingdom of Morocco	7.64 (2.34,13.59)	3.13 (0.9,5.54)	4.51 (1.45,8.39)	179.56 (61.06,303.4)	72.9 (23.24,123.51)	106.66 (37.47,191.88)
Kingdom of Norway	0.52 (0.15,0.93)	0.21 (0.06,0.36)	0.32 (0.09,0.56)	10.01 (3.16,16.8)	4.92 (1.62,8.1)	5.08 (1.56,8.73)
Kingdom of Saudi Arabia	2.59 (0.88,4.21)	1.53 (0.53,2.52)	1.06 (0.37,1.72)	90.03 (33.56,140.04)	53.66 (20.45,84.23)	36.37 (13.93,56.4)
Kingdom of Spain	5.11 (1.45,9.3)	1.93 (0.59,3.44)	3.18 (0.89,5.85)	87.88 (27.72,149.98)	42.62 (13.88,70.62)	45.26 (13.79,78.99)

Kingdom of Sweden	1.28 (0.36,2.31)	0.54 (0.15,0.95)	0.74 (0.2,1.36)	22.22 (7.12,38.14)	10.96 (3.51,18.64)	11.25 (3.47,19.93)
Kingdom of Thailand	8.69 (2.85,15.38)	4.14 (1.4,7.45)	4.55 (1.44,8.28)	218.62 (83.33,358.18)	111.36 (44.57,184.99)	107.26 (39.53,173.26)
Kingdom of the Netherlands	2.46 (0.74,4.36)	0.97 (0.3,1.68)	1.49 (0.44,2.67)	40.69 (13.29,68.33)	18.69 (6.16,31.06)	22 (7.08,37.69)
Kingdom of Tonga	0.01 (0,0.01)	0 (0,0)	0 (0,0.01)	0.15 (0.05,0.24)	0.07 (0.03,0.11)	0.08 (0.03,0.13)
Kyrgyz Republic	0.71 (0.23,1.17)	0.4 (0.13,0.66)	0.31 (0.1,0.52)	20.7 (7.68,33.06)	12.07 (4.42,19.22)	8.64 (3.21,13.96)
Lao People's Democratic Republic	0.66 (0.21,1.17)	0.35 (0.11,0.62)	0.32 (0.1,0.55)	17.34 (6.28,28.67)	9.37 (3.22,15.92)	7.97 (2.9,12.96)
Lebanese Republic	0.55 (0.17,0.99)	0.26 (0.08,0.47)	0.28 (0.09,0.51)	11.63 (4.17,18.69)	5.92 (2.1,9.46)	5.71 (2.05,9.38)
Malaysia	3.15 (1.08,5.12)	1.52 (0.52,2.46)	1.64 (0.55,2.78)	85.53 (32.69,131.11)	43.8 (17,67.14)	41.73 (15.92,66.29)
Mongolia	0.12 (0.04,0.2)	0.07 (0.03,0.12)	0.05 (0.02,0.09)	4.5 (1.82,6.93)	2.48 (0.97,3.92)	2.02 (0.81,3.22)
Montenegro	0.15 (0.04,0.27)	0.06 (0.02,0.11)	0.08 (0.02,0.16)	2.63 (0.82,4.58)	1.23 (0.39,2.09)	1.41 (0.42,2.48)
New Zealand	0.5 (0.14,0.88)	0.19 (0.05,0.34)	0.31 (0.09,0.55)	8.32 (2.71,13.93)	3.72 (1.24,6.21)	4.6 (1.47,7.77)
North Macedonia	1.4 (0.43,2.44)	0.59 (0.18,1.02)	0.81 (0.24,1.45)	25.4 (8.27,42.8)	11.72 (3.89,19.64)	13.68 (4.39,23.69)
Northern Mariana Islands	0 (0,0)	0 (0,0)	0 (0,0)	0.09 (0.03,0.14)	0.05 (0.02,0.08)	0.04 (0.01,0.06)
Palestine	0.42 (0.14,0.72)	0.19 (0.06,0.33)	0.23 (0.07,0.41)	9.8 (3.65,15.63)	4.8 (1.78,7.67)	4.99 (1.84,8.11)
People's Democratic Republic of Algeria	4.89 (1.51,8.76)	2.13 (0.66,3.86)	2.76 (0.83,5.14)	112.03 (41.47,186.89)	49.43 (17.09,84.93)	62.6 (23.14,111)
People's Republic of Bangladesh	14.41 (3.91,27.59)	7.27 (2.1,14.49)	7.14 (1.91,14.25)	299.39 (92.3,569.96)	157.84 (47.62,309.15)	141.55 (43.22,265.59)
People's Republic of China	300.05 (92.52,527.46)	170.52 (55.1,298.99)	129.53 (39.34,233.38)	6850.57 (2313.2,11418.17)	3947.47 (1379.35,6623.02)	2903.1 (984.54,4925.9)
Plurinational State of Bolivia	0.57 (0.17,1.03)	0.25 (0.07,0.46)	0.32 (0.09,0.58)	12.23 (4.27,20.91)	5.63 (1.89,9.76)	6.6 (2.25,11.73)
Portuguese Republic	2.64 (0.76,4.69)	1.04 (0.3,1.8)	1.59 (0.44,2.84)	36.3 (11.45,62.46)	16.48 (5.33,27.79)	19.82 (5.82,34.44)
Principality of Andorra	0.01 (0,0.01)	0 (0,0)	0 (0,0.01)	0.13 (0.04,0.22)	0.06 (0.02,0.1)	0.07 (0.02,0.13)
Principality of Monaco	0.01 (0,0.02)	0 (0,0.01)	0.01 (0,0.01)	0.17 (0.06,0.3)	0.07 (0.02,0.13)	0.1 (0.03,0.18)
Puerto Rico	0.23 (0.07,0.42)	0.1 (0.03,0.19)	0.13 (0.04,0.23)	4.23 (1.43,7.15)	1.99 (0.68,3.34)	2.24 (0.74,3.77)
Republic of Albania	0.49 (0.15,0.89)	0.23 (0.07,0.42)	0.26 (0.07,0.52)	8.86 (2.91,15.37)	4.36 (1.42,7.73)	4.5 (1.43,7.97)
Republic of Angola	0.98 (0.3,1.77)	0.44	0.55 (0.16,1)	28.14 (9.75,47.8)	12.48	15.65

		(0.14,0.82)			(4.34,22.35)	(5.28,26.62)
Republic of Armenia	0.49 (0.14,0.87)	0.21 (0.06,0.38)	0.28 (0.08,0.5)	10.76 (3.47,18.06)	5.37 (1.77,9.01)	5.39 (1.68,9.26)
Republic of Austria	0.92 (0.27,1.61)	0.38 (0.12,0.65)	0.54 (0.16,0.97)	19.54 (6.26,32.42)	9.34 (3.1,15.27)	10.2 (3.19,17.21)
Republic of Azerbaijan	0.79 (0.24,1.45)	0.36 (0.11,0.69)	0.43 (0.12,0.84)	22.05 (7.85,37.53)	10.3 (3.56,18.35)	11.75 (4.08,20.92)
Republic of Belarus	3.34 (1.02,5.82)	1.33 (0.42,2.25)	2.01 (0.59,3.62)	69.96 (22.88,115.34)	33.63 (11.46,55.44)	36.33 (11.56,61.6)
Republic of Benin	0.62 (0.19,1.11)	0.31 (0.1,0.56)	0.3 (0.09,0.57)	15.32 (5.15,26.57)	8.22 (2.88,14.18)	7.09 (2.37,12.27)
Republic of Botswana	0.11 (0.03,0.19)	0.05 (0.01,0.08)	0.06 (0.02,0.11)	2.89 (0.98,4.91)	1.36 (0.44,2.24)	1.53 (0.52,2.67)
Republic of Bulgaria	6.11 (1.87,10.43)	2.83 (0.9,4.78)	3.28 (0.96,5.72)	109.9 (37.3,184.82)	56.93 (20.1,93.25)	52.97 (16.94,90.03)
Republic of Burundi	0.32 (0.1,0.62)	0.15 (0.04,0.28)	0.18 (0.06,0.36)	9.07 (3.19,16.53)	4.16 (1.37,7.52)	4.91 (1.79,8.87)
Republic of Cabo Verde	0.06 (0.02,0.1)	0.03 (0.01,0.06)	0.03 (0.01,0.05)	1.36 (0.47,2.23)	0.85 (0.3,1.44)	0.52 (0.17,0.85)
Republic of Cameroon	0.94 (0.26,1.81)	0.56 (0.16,1.1)	0.38 (0.1,0.73)	26.46 (8.05,49.34)	16.11 (5.03,30.38)	10.34 (3.06,19.19)
Republic of Chad	0.71 (0.21,1.37)	0.47 (0.14,0.86)	0.25 (0.07,0.51)	19.21 (6.23,34.49)	12.37 (4.02,22.38)	6.84 (2.19,12.7)
Republic of Chile	1.53 (0.46,2.72)	0.72 (0.23,1.25)	0.81 (0.23,1.46)	30.56 (10.57,50.35)	15.9 (5.57,25.56)	14.67 (4.97,24.82)
Republic of Colombia	2.24 (0.69,3.95)	1.08 (0.33,1.88)	1.16 (0.35,2.07)	47.34 (16.77,78.11)	23.12 (8.03,38.45)	24.21 (8.54,39.87)
Republic of Costa Rica	0.24 (0.07,0.43)	0.11 (0.03,0.19)	0.14 (0.04,0.24)	4.68 (1.65,7.64)	2.14 (0.75,3.54)	2.54 (0.9,4.12)
Republic of Croatia	1.36 (0.41,2.44)	0.52 (0.16,0.91)	0.85 (0.24,1.5)	22.93 (7.45,38.88)	10.39 (3.52,17.59)	12.54 (3.91,21.62)
Republic of Cuba	1.82 (0.54,3.2)	0.86 (0.26,1.53)	0.95 (0.27,1.71)	33.4 (11.24,56.51)	17.09 (5.81,29.06)	16.31 (5.31,27.66)
Republic of Cyprus	0.14 (0.04,0.26)	0.06 (0.02,0.11)	0.08 (0.02,0.16)	2.16 (0.7,3.75)	1 (0.33,1.73)	1.17 (0.37,2.06)
Republic of C 么 te d'Ivoire	1.38 (0.44,2.47)	0.92 (0.3,1.67)	0.46 (0.14,0.86)	39.97 (14.8,67.71)	27.28 (10.07,48.45)	12.7 (4.41,21.76)
Republic of Djibouti	0.04 (0.01,0.08)	0.03 (0.01,0.05)	0.02 (0,0.03)	1.31 (0.46,2.37)	0.76 (0.27,1.46)	0.54 (0.18,0.97)
Republic of Ecuador	0.72 (0.22,1.31)	0.36 (0.11,0.65)	0.35 (0.11,0.64)	15.24 (5.39,25.97)	8 (2.78,13.29)	7.24 (2.57,12.33)
Republic of El Salvador	0.3 (0.09,0.53)	0.13 (0.04,0.24)	0.17 (0.05,0.31)	6.02 (2,9.94)	2.73 (0.96,4.64)	3.29 (1.11,5.42)
Republic of Equatorial Guinea	0.04	0.02 (0,0.03)	0.03	1.18 (0.41,2)	0.48 (0.17,0.84)	0.69 (0.22,1.24)

	(0.01,0.08)		(0.01,0.05)			
Republic of Estonia	0.28 (0.09,0.47)	0.12 (0.04,0.2)	0.16 (0.04,0.27)	5.25 (1.8,8.59)	2.68 (0.96,4.3)	2.57 (0.82,4.35)
Republic of Fiji	0.06 (0.02,0.1)	0.03 (0.01,0.05)	0.03 (0.01,0.06)	1.83 (0.72,2.92)	0.89 (0.34,1.42)	0.94 (0.36,1.55)
Republic of Finland	0.88 (0.26,1.56)	0.36 (0.11,0.63)	0.52 (0.15,0.92)	15.75 (5.01,26.84)	7.64 (2.5,12.66)	8.11 (2.5,13.93)
Republic of Ghana	3.44 (1.06,5.9)	2 (0.63,3.36)	1.45 (0.44,2.69)	96.95 (33.7,161.16)	58.48 (20.36,93.27)	38.47 (13.41,66.49)
Republic of Guatemala	0.39 (0.12,0.7)	0.19 (0.06,0.33)	0.21 (0.06,0.37)	8.42 (2.97,14.11)	3.91 (1.33,6.57)	4.51 (1.63,7.57)
Republic of Guinea	0.8 (0.25,1.5)	0.48 (0.15,0.9)	0.32 (0.1,0.62)	20.5 (7.12,35.89)	12.47 (4.23,22.25)	8.03 (2.77,13.91)
Republic of Guinea-Bissau	0.12 (0.04,0.21)	0.07 (0.02,0.12)	0.05 (0.01,0.09)	3.48 (1.25,5.82)	2.13 (0.76,3.48)	1.36 (0.44,2.4)
Republic of Guyana	0.09 (0.03,0.16)	0.04 (0.01,0.08)	0.05 (0.02,0.09)	2.08 (0.74,3.49)	1.05 (0.38,1.76)	1.03 (0.36,1.72)
Republic of Haiti	1.04 (0.32,1.99)	0.4 (0.12,0.76)	0.65 (0.2,1.27)	24.98 (8.37,45.74)	9.44 (3.05,18.23)	15.54 (5.06,28.78)
Republic of Honduras	0.71 (0.22,1.25)	0.34 (0.1,0.58)	0.37 (0.12,0.68)	14.83 (5.12,25)	7.08 (2.26,11.67)	7.74 (2.77,13.4)
Republic of Iceland	0.03 (0.01,0.05)	0.01 (0,0.02)	0.02 (0,0.03)	0.5 (0.16,0.84)	0.23 (0.08,0.39)	0.27 (0.09,0.45)
Republic of India	74.25 (25.18,131.56)	37.14 (12.17,67.78)	37.11 (12.02,66.66)	1818.15 (669.98,3090.36)	934.35 (333.9,1645.76)	883.81 (314.71,1514.73)
Republic of Indonesia	33.02 (10.49,57.68)	16.93 (5.19,30.68)	16.09 (4.77,30.41)	895.59 (317.88,1520.58)	467.96 (158,804.43)	427.64 (147.78,752.07)
Republic of Iraq	6.08 (2.03,10.23)	3.27 (1.09,5.52)	2.8 (0.93,4.73)	152.9 (58.88,243.94)	83.55 (31.65,136.9)	69.35 (26.65,110.71)
Republic of Italy	11 (3.19,19.91)	4.11 (1.18,7.38)	6.89 (1.95,12.5)	151.96 (46.52,268.47)	66.05 (21.19,114.47)	85.91 (25.45,152.43)
Republic of Kazakhstan	3.52 (1.11,6.16)	1.57 (0.51,2.67)	1.95 (0.58,3.56)	84.86 (30.58,138.25)	41.94 (15.44,67.86)	42.92 (14.76,71.89)
Republic of Kenya	1.11 (0.34,2.07)	0.45 (0.14,0.85)	0.65 (0.19,1.29)	31.36 (10.7,53.6)	13.26 (4.36,23.42)	18.1 (6.14,32.68)
Republic of Kiribati	0.01 (0,0.01)	0 (0,0.01)	0 (0,0)	0.23 (0.09,0.37)	0.14 (0.06,0.23)	0.09 (0.04,0.15)
Republic of Korea	5.65 (1.56,10.37)	2.54 (0.76,4.6)	3.11 (0.82,5.8)	123.53 (41.23,208.2)	66.63 (22.72,109.66)	56.89 (18.67,97.58)
Republic of Latvia	1.25 (0.38,2.22)	0.42 (0.14,0.72)	0.83 (0.24,1.46)	20.87 (6.84,35.07)	8.81 (2.98,14.46)	12.06 (3.76,20.86)
Republic of Liberia	0.25 (0.08,0.46)	0.14 (0.05,0.26)	0.11 (0.03,0.21)	6.91 (2.49,12.04)	4.03 (1.41,7.18)	2.88 (0.97,5.32)
Republic of Lithuania	1.17	0.45	0.71 (0.2,1.23)	20.91	9.89 (3.58,15.55)	11.02

	(0.36,1.99)	(0.15,0.76)		(7.13,33.91)		(3.45,18.51)
Republic of Madagascar	0.98 (0.31,1.76)	0.46 (0.15,0.89)	0.52 (0.16,0.97)	30.6 (10.77,52.67)	14.08 (4.86,25.49)	16.52 (5.5,29.03)
Republic of Malawi	0.76 (0.23,1.4)	0.36 (0.12,0.66)	0.41 (0.11,0.79)	20.17 (6.85,34.64)	10.02 (3.48,17.43)	10.15 (3.22,18.46)
Republic of Maldives	0.03 (0.01,0.05)	0.02 (0,0.03)	0.01 (0,0.02)	0.73 (0.28,1.16)	0.44 (0.17,0.71)	0.28 (0.1,0.45)
Republic of Mali	0.73 (0.21,1.42)	0.37 (0.1,0.69)	0.36 (0.1,0.77)	19.77 (6.39,35.95)	9.95 (3.05,17.84)	9.82 (3.28,19.17)
Republic of Malta	0.05 (0.01,0.09)	0.02 (0.01,0.04)	0.03 (0.01,0.06)	0.82 (0.27,1.41)	0.37 (0.12,0.6)	0.46 (0.14,0.79)
Republic of Mauritius	0.17 (0.06,0.29)	0.09 (0.03,0.15)	0.08 (0.03,0.14)	4.32 (1.61,6.75)	2.38 (0.9,3.71)	1.94 (0.72,3.08)
Republic of Moldova	0.82 (0.25,1.43)	0.38 (0.12,0.65)	0.44 (0.13,0.79)	18.75 (6.43,30.96)	9.51 (3.36,15.61)	9.25 (3.06,15.64)
Republic of Mozambique	1.73 (0.55,2.95)	1.01 (0.34,1.82)	0.72 (0.21,1.33)	47.49 (16.85,78.76)	29.05 (10.24,50.38)	18.44 (5.94,32.62)
Republic of Namibia	0.15 (0.05,0.29)	0.06 (0.02,0.12)	0.09 (0.03,0.17)	3.61 (1.17,6.44)	1.64 (0.57,2.91)	1.96 (0.62,3.57)
Republic of Nauru	0 (0,0)	0 (0,0)	0 (0,0)	0.03 (0.01,0.05)	0.01 (0,0.02)	0.02 (0.01,0.03)
Republic of Nicaragua	0.19 (0.06,0.34)	0.09 (0.03,0.16)	0.1 (0.03,0.19)	4.42 (1.66,7.44)	2.05 (0.72,3.56)	2.37 (0.89,3.95)
Republic of Niue	0 (0,0)	0 (0,0)	0 (0,0)	0 (0,0.01)	0 (0,0)	0 (0,0)
Republic of Palau	0 (0,0)	0 (0,0)	0 (0,0)	0.07 (0.03,0.11)	0.04 (0.01,0.07)	0.03 (0.01,0.05)
Republic of Panama	0.28 (0.08,0.49)	0.14 (0.04,0.26)	0.14 (0.04,0.24)	4.98 (1.69,8.52)	2.56 (0.85,4.54)	2.42 (0.84,3.96)
Republic of Paraguay	0.57 (0.18,1.02)	0.29 (0.1,0.53)	0.28 (0.08,0.49)	10.95 (3.95,18.53)	6.06 (2.22,10.44)	4.89 (1.61,8.24)
Republic of Peru	1.26 (0.4,2.32)	0.6 (0.19,1.11)	0.66 (0.2,1.2)	27.79 (10.1,46.77)	13.63 (4.95,23.52)	14.16 (5.05,23.86)
Republic of Poland	9.39 (2.77,16.56)	3.85 (1.23,6.53)	5.54 (1.59,9.96)	172.44 (58.02,288.2)	85.84 (30.84,138.56)	86.6 (27.18,148.16)
Republic of Rwanda	0.3 (0.08,0.6)	0.12 (0.03,0.25)	0.18 (0.05,0.37)	8.06 (2.38,15.11)	3.3 (0.99,6.58)	4.76 (1.44,9.09)
Republic of San Marino	0 (0,0.01)	0 (0,0)	0 (0,0.01)	0.08 (0.02,0.14)	0.04 (0.01,0.07)	0.04 (0.01,0.07)
Republic of Senegal	1.01 (0.3,1.83)	0.54 (0.16,1.01)	0.47 (0.13,0.91)	25.42 (8.3,42.89)	13.86 (4.56,23.98)	11.56 (3.79,20.7)
Republic of Serbia	6.97 (2.04,12.34)	2.89 (0.88,5.06)	4.08 (1.21,7.23)	115.89 (36.01,196.49)	53.29 (17.24,89.66)	62.61 (19.57,107.56)
Republic of Seychelles	0.01 (0,0.02)	0.01 (0,0.01)	0.01 (0,0.01)	0.32 (0.12,0.5)	0.18 (0.07,0.29)	0.14 (0.05,0.22)
Republic of Sierra Leone	0.53 (0.16,0.94)	0.3 (0.09,0.53)	0.23 (0.07,0.45)	14.3 (4.88,23.92)	8.28 (2.77,13.96)	6.03 (1.99,10.62)
Republic of Singapore	0.16 (0.05,0.28)	0.06 (0.02,0.11)	0.1 (0.03,0.17)	5.94 (2.13,9.37)	3.1 (1.19,4.84)	2.84 (1.01,4.55)

Republic of Slovenia	0.39 (0.11,0.7)	0.17 (0.05,0.29)	0.22 (0.06,0.42)	6.4 (2.06,10.88)	3.18 (1.06,5.38)	3.22 (0.99,5.64)
Republic of South Africa	4.72 (1.45,8.46)	1.71 (0.57,2.98)	3.02 (0.91,5.42)	112.46 (39.47,188.26)	45.05 (16.58,74.11)	67.4 (22.75,113.84)
Republic of South Sudan	0.22 (0.06,0.41)	0.11 (0.03,0.22)	0.1 (0.03,0.2)	6.23 (2.06,11.05)	3.12 (0.98,5.8)	3.11 (1.01,5.41)
Republic of Sudan	3.2 (0.98,5.77)	1.57 (0.47,2.86)	1.62 (0.52,3.28)	87.04 (30.57,152.55)	41.77 (14.6,71.02)	45.27 (16.35,86.5)
Republic of Suriname	0.07 (0.02,0.12)	0.03 (0.01,0.06)	0.04 (0.01,0.06)	1.44 (0.49,2.36)	0.74 (0.27,1.21)	0.71 (0.24,1.19)
Republic of Tajikistan	0.75 (0.23,1.28)	0.37 (0.11,0.66)	0.38 (0.11,0.67)	20.32 (6.83,32.91)	9.81 (3.14,16.36)	10.5 (3.68,17.24)
Republic of the Congo	0.26 (0.08,0.47)	0.11 (0.03,0.19)	0.16 (0.05,0.29)	7.4 (2.53,12.62)	3.04 (1.09,5.12)	4.36 (1.48,7.5)
Republic of the Gambia	0.16 (0.05,0.29)	0.09 (0.03,0.15)	0.07 (0.02,0.15)	4.11 (1.4,7.14)	2.37 (0.82,4.09)	1.73 (0.52,3.31)
Republic of the Marshall Islands	0 (0,0)	0 (0,0)	0 (0,0)	0.1 (0.04,0.17)	0.05 (0.02,0.09)	0.06 (0.02,0.09)
Republic of the Niger	0.65 (0.18,1.26)	0.35 (0.1,0.7)	0.29 (0.07,0.62)	17.45 (5.58,32.22)	9.26 (2.78,17.5)	8.19 (2.4,16.11)
Republic of the Philippines	8.39 (2.83,14.13)	3.98 (1.34,6.74)	4.41 (1.53,7.77)	233.29 (88.86,367.55)	117.9 (44.12,188.84)	115.39 (43.66,183.74)
Republic of the Union of Myanmar	7.08 (2.16,12.45)	3.7 (1.17,6.54)	3.37 (0.95,6.14)	170.48 (57.62,289.22)	92.8 (32.34,153.61)	77.68 (24.78,133.62)
Republic of Trinidad and Tobago	0.21 (0.06,0.36)	0.11 (0.04,0.19)	0.1 (0.03,0.17)	4.14 (1.42,6.84)	2.4 (0.84,3.94)	1.75 (0.58,2.92)
Republic of Tunisia	1.93 (0.59,3.5)	0.98 (0.31,1.81)	0.94 (0.25,1.83)	41.33 (14.06,70.92)	21.67 (7.41,38.16)	19.66 (6.69,36.24)
Republic of Turkey	9.72 (2.81,17.81)	4.14 (1.25,7.44)	5.58 (1.58,10.51)	200.16 (66.52,339.31)	93.32 (31.2,157.15)	106.84 (34.59,186.77)
Republic of Uganda	0.69 (0.18,1.35)	0.29 (0.07,0.56)	0.4 (0.1,0.79)	20.06 (6.22,35.61)	8.59 (2.48,16.04)	11.47 (3.52,20.2)
Republic of Uzbekistan	3.72 (1.22,6.15)	1.94 (0.63,3.24)	1.78 (0.59,2.96)	109.59 (40.28,171.08)	58.3 (20.94,91.32)	51.29 (19.33,79.04)
Republic of Vanuatu	0.02 (0.01,0.03)	0.01 (0,0.02)	0.01 (0,0.01)	0.56 (0.22,0.89)	0.3 (0.11,0.48)	0.26 (0.1,0.44)
Republic of Yemen	3.23 (1.06,5.96)	1.59 (0.48,2.91)	1.64 (0.52,3.13)	84.24 (29.49,145.6)	41.25 (13.69,70.78)	42.99 (15.09,77.51)
Republic of Zambia	0.51 (0.14,1.01)	0.22 (0.06,0.45)	0.29 (0.08,0.56)	14.05 (4.54,26.35)	6.16 (2.04,11.66)	7.88 (2.44,14.1)
Republic of Zimbabwe	0.7 (0.22,1.28)	0.3 (0.1,0.53)	0.4 (0.12,0.75)	19 (6.44,33.46)	8.16 (2.84,13.91)	10.85 (3.46,19.28)
Romania	11 (3.3,19.44)	4.69 (1.45,8.16)	6.31 (1.85,11.25)	186.9 (59.62,316.66)	90.05 (29.24,150.36)	96.84 (30.25,168.26)
Russian Federation	75.13	28.54	46.59	1456.6	672.01	784.6

	(24.89,124.87)	(9.89,45.87)	(14,79.36)	(522.83,2317.35)	(254.97,1033.23)	(260.45,1291.01)
Saint Kitts and Nevis	0.01 (0,0.02)	0 (0,0.01)	0 (0,0.01)	0.19 (0.07,0.32)	0.1 (0.04,0.16)	0.1 (0.03,0.16)
Saint Lucia	0.03 (0.01,0.05)	0.01 (0,0.02)	0.02 (0,0.03)	0.44 (0.14,0.75)	0.18 (0.06,0.32)	0.26 (0.08,0.44)
Saint Vincent and the Grenadines	0.01 (0,0.02)	0.01 (0,0.01)	0.01 (0,0.01)	0.25 (0.08,0.43)	0.11 (0.04,0.19)	0.14 (0.05,0.24)
Slovak Republic	1.37 (0.4,2.44)	0.61 (0.19,1.05)	0.76 (0.21,1.37)	29.3 (9.39,49.8)	14.75 (5.01,24.33)	14.55 (4.54,24.73)
Socialist Republic of Viet Nam	18.66 (5.92,33.43)	10.35 (3.46,18.48)	8.31 (2.26,16)	414 (146.1,712.93)	255.88 (91.87,444.15)	158.12 (50.97,279.48)
Solomon Islands	0.03 (0.01,0.06)	0.01 (0,0.03)	0.02 (0,0.03)	0.95 (0.34,1.59)	0.41 (0.13,0.75)	0.54 (0.2,0.9)
State of Eritrea	0.18 (0.05,0.33)	0.07 (0.02,0.14)	0.11 (0.03,0.22)	5.46 (1.84,9.7)	2.24 (0.71,4.15)	3.22 (1.08,5.81)
State of Israel	0.4 (0.11,0.71)	0.16 (0.05,0.28)	0.23 (0.06,0.43)	8.5 (2.82,14.19)	4.22 (1.45,6.96)	4.28 (1.4,7.26)
State of Kuwait	0.18 (0.06,0.29)	0.13 (0.04,0.21)	0.05 (0.02,0.08)	6.09 (2.53,9.15)	4.03 (1.59,6.22)	2.07 (0.89,2.98)
State of Libya	0.7 (0.23,1.27)	0.31 (0.1,0.58)	0.38 (0.13,0.73)	19.78 (7.48,33.33)	8.85 (3.22,15.29)	10.93 (4.12,18.91)
State of Qatar	0.04 (0.01,0.06)	0.02 (0.01,0.04)	0.01 (0,0.02)	1.81 (0.77,2.81)	1.29 (0.55,2.01)	0.52 (0.22,0.81)
Sultanate of Oman	0.23 (0.08,0.39)	0.14 (0.05,0.23)	0.09 (0.03,0.16)	7.44 (3.02,11.46)	4.69 (1.95,7.06)	2.75 (1.09,4.25)
Swiss Confederation	0.84 (0.24,1.5)	0.34 (0.1,0.59)	0.5 (0.14,0.92)	13.84 (4.46,23.11)	6.68 (2.17,11.13)	7.16 (2.19,12.27)
Syrian Arab Republic	1.84 (0.61,3.34)	0.96 (0.31,1.75)	0.88 (0.28,1.61)	46.3 (16.58,80.06)	24.49 (8.66,41.76)	21.81 (7.91,36.74)
Taiwan (Province of China)	1.9 (0.6,3.25)	0.99 (0.31,1.7)	0.91 (0.27,1.6)	55.82 (19.62,88.28)	29.14 (10.49,46.14)	26.68 (9.26,42.99)
Togolese Republic	0.52 (0.16,0.91)	0.31 (0.1,0.52)	0.21 (0.06,0.41)	14.93 (5.2,24.93)	9.14 (3.23,14.84)	5.79 (1.94,10.03)
Tokelau	0 (0,0)	0 (0,0)	0 (0,0)	0 (0,0.01)	0 (0,0)	0 (0,0)
Turkmenistan	0.89 (0.3,1.57)	0.44 (0.15,0.78)	0.45 (0.15,0.8)	25.59 (9.57,42.48)	13.1 (4.87,22.45)	12.48 (4.79,20.61)
Tuvalu	0 (0,0)	0 (0,0)	0 (0,0)	0.03 (0.01,0.05)	0.01 (0,0.03)	0.02 (0,0.03)
Ukraine	19.54 (6.54,33.96)	7.93 (2.94,13.86)	11.61 (3.33,21.37)	398.53 (154.2,659.36)	188.87 (78.36,319.89)	209.66 (70.22,367.23)
Union of the Comoros	0.04 (0.01,0.07)	0.02 (0,0.03)	0.02 (0.01,0.04)	1.08 (0.37,1.78)	0.45 (0.16,0.8)	0.63 (0.22,1.07)
United Arab Emirates	0.26 (0.1,0.41)	0.15 (0.06,0.24)	0.11 (0.04,0.18)	13.43 (5.97,19.82)	9.1 (4.08,13.57)	4.33 (1.83,6.56)
United Kingdom of Great Britain and Northern Ireland	6.75 (1.91,11.97)	2.75 (0.79,4.85)	4 (1.13,7.14)	118.01 (37.04,200.35)	54.99 (17.8,92.02)	63.02 (19.46,107.44)

United Mexican States	5.54 (1.7,9.43)	2.69 (0.88,4.67)	2.86 (0.9,4.89)	127.64 (47.35,201.36)	63.86 (23.65,102.34)	63.78 (23.03,102.24)
United Republic of Tanzania	2.39 (0.71,4.14)	1.18 (0.35,2.18)	1.21 (0.34,2.26)	61.51 (20.82,101.68)	30.31 (10.29,53.04)	31.2 (10.36,53.86)
United States of America	26.96 (7.58,48.52)	10.23 (3.17,18.16)	16.74 (4.65,30.53)	599.4 (201.58,1007.29)	266.98 (92.99,440.99)	332.41 (108.54,565.59)
United States Virgin Islands	0.01 (0,0.01)	0 (0,0.01)	0 (0,0.01)	0.16 (0.05,0.28)	0.09 (0.03,0.14)	0.08 (0.03,0.14)

Table S5 ASMR and ASDR in 204 countries in 2021

Location	ASMR/100,000 persons（95%UI）			ASDR/100,000 persons（95%UI）		
	Both	Male	Female	Both	Male	Female
American Samoa	8.66 (2.7,15.48)	7.77 (2.37,13.68)	9.44 (2.83,17.03)	203.34 (75.57,345.9)	187.83 (67.04,310.95)	217.95 (77.2,373.08)
Antigua and Barbuda	9.03 (2.67,16.02)	8.66 (2.49,15.62)	9.04 (2.71,15.97)	158.51 (50.77,267.33)	156.08 (48.86,265.82)	157.79 (51.95,264.3)
Arab Republic of Egypt	36.87 (11.82,64.42)	29.75 (9.3,52.94)	55.55 (17.6,98.96)	740.77 (254.54,1222.12)	652.56 (223.62,1151.07)	941.94 (328.56,1592.91)
Argentine Republic	5.25 (1.62,9.23)	6.19 (1.89,10.74)	4.5 (1.39,8.04)	116.59 (40.45,188.8)	140.2 (47.67,226.99)	97.59 (33.83,160.31)
Australia	3.69 (1.06,6.57)	3.65 (1.05,6.49)	3.61 (1.03,6.49)	77.86 (26.31,128.01)	87.11 (29.92,142.27)	68.64 (23.01,115.28)
Barbados	11.13 (3.31,19.63)	10.46 (3.14,18.42)	11.53 (3.41,20.38)	192.49 (63.48,326.63)	187.34 (61.3,318.9)	195.38 (65.96,331.66)
Belize	6.34 (1.91,11.09)	5.92 (1.75,10.54)	6.69 (2.02,11.73)	120.81 (41.42,200.75)	118.59 (39.16,201.01)	122.48 (41.45,201.32)
Bermuda	5.13 (1.53,9.04)	5.94 (1.76,10.52)	4.42 (1.29,7.82)	97.51 (32.88,163.75)	113.41 (37.34,192.72)	83.95 (29.54,138.99)
Bolivarian Republic of Venezuela	6.32 (1.84,11.37)	6.56 (1.88,11.83)	6.05 (1.8,11.11)	117.39 (38.72,200.29)	122.23 (38.62,213.76)	112.75 (38.29,196.16)
Bosnia and Herzegovina	25.94 (8.75,44.86)	26.59 (9.14,46.23)	24.98 (8.01,43.99)	512.82 (184.05,842.33)	559.16 (203.95,928.26)	468.2 (162.24,781.2)
Brunei Darussalam	10.44 (3.03,18.38)	11.84 (3.27,21.63)	9.94 (3.01,17.63)	212.58 (71.25,355.14)	242.21 (80.39,420.39)	192.91 (65.34,325.71)
Burkina Faso	6.74 (1.84,13.44)	9.24 (2.5,18.88)	4.63 (1.25,9.47)	145.3 (43.32,277.98)	195.88 (56.17,379.91)	100.6 (32.08,187.5)
Canada	3.12 (0.87,5.51)	3.12 (0.93,5.51)	3.03 (0.82,5.39)	83.84 (29.32,137.45)	90.36 (32.19,146.88)	77.44 (26.6,128.58)
Central African Republic	14.44 (3.77,28.43)	13.15 (3.53,25.5)	14.83 (3.65,29.91)	295.64 (85.39,545.38)	280.47 (79.45,532.29)	299.11 (86.27,574.71)
Commonwealth of Dominica	12.04 (3.48,22.14)	11.05 (3.12,20.14)	12.13 (3.62,23.06)	209.38 (64.85,368.59)	203.2 (64.54,348.84)	205.99 (65.73,369.58)
Commonwealth of the Bahamas	7.33 (2.29,13.29)	6.58 (2.08,12)	7.81 (2.43,14.26)	143.4 (49.43,249.6)	134.54 (46.52,228.26)	149.1 (51.4,259.56)

Cook Islands	6.03	6.34	5.7	160.51	167.65	153.12
	(1.77,10.97)	(1.88,11.71)	(1.66,10.86)	(57.93,264.91)	(59.24,281.44)	(54.07,261.31)
Czech Republic	9.05	10.28	7.94	191.74	227.82	160.35
	(2.86,15.64)	(3.32,17.42)	(2.45,14.04)	(68.44,306.38)	(80.71,367.59)	(55.96,261.54)
Democratic People's Republic of	18.98	22.52	16.66	459.96	540.92	400.47
Korea	(6.17,34)	(6.98,40.58)	(5.25,31.63)	(164.35,779.05)	(188.87,911.73)	(144.19,688.28)
Democratic Republic of Sao Tome and	16.71	17.28	15.9	369.06	389.03	345.81
Principe	(4.81,29.27)	(5.14,31.28)	(4.5,27.91)	(120.72,620.52)	(126.67,668.06)	(112.85,577.53)
Democratic Republic of the Congo	11.51	10.61	11.91	232.91	214.98	243.18
	(3.01,23.7)	(2.68,21.45)	(3.09,26.02)	(71.62,451.27)	(57.82,429.14)	(77.23,500.19)
Democratic Republic of Timor-Leste	15.06	14.89	15.18	305.91	311.06	299.79
	(4.42,28.59)	(4.18,28.64)	(4.3,30.3)	(100.78,537.26)	(96.8,578.43)	(99.5,551.46)
Democratic Socialist Republic of Sri	19.31	20.76	18.19	343.11	374.58	317.34
Lanka	(5.55,34.17)	(6.1,37.88)	(5.36,31.76)	(118.4,577.79)	(129.47,644.35)	(111,528.84)
Dominican Republic	9.22	9.36	8.98	195.62	207.01	183.71
	(2.85,17.31)	(2.98,17.03)	(2.8,16.01)	(69.42,336.68)	(70.74,365.55)	(65.01,311.28)
Eastern Republic of Uruguay	9.42	10.56	8.44	183.72	216.47	156.81
	(2.9,16.45)	(3.33,18.19)	(2.48,14.78)	(63.63,297.86)	(76.37,348.55)	(53.54,256.96)
Federal Democratic Republic of	4.46	3.79	5.16	98.61	81.29	116.7
Ethiopia	(1.18,8.55)	(0.93,7.93)	(1.48,10.09)	(31.02,178.72)	(22.58,164.61)	(37.09,212.43)
Federal Democratic Republic of Nepal	8.85	9.46	8.31	175.56	189.63	162.81
	(2.33,16.93)	(2.63,18.9)	(2.13,15.69)	(50.38,324.67)	(58.14,371.59)	(48.48,299.65)
Federal Republic of Germany	5.24 (1.67,9.1)	6.01	4.55	126.66	147.61	107.74
		(1.96,10.22)	(1.37,8.1)	(44.97,204.64)	(52.02,236.71)	(37.68,173.29)
Federal Republic of Nigeria	13.29	17.02	10.09	276.21	358.64	207.39
	(4.13,22.49)	(5.4,31.2)	(2.7,18.98)	(92.78,452.49)	(120.52,630.99)	(63.84,358.67)
Federal Republic of Somalia	7.68	6.85	8.07 (2,17.06)	172.41	155.49	182.36
	(1.97,15.58)	(1.56,15.81)		(49.51,325.75)	(40.95,337.56)	(53.24,354.71)
Federated States of Micronesia	14.26	14.67	13.73	331.45	341.85	320.06
	(4.34,26.16)	(4.47,29.65)	(4.16,26.09)	(116.24,563.69)	(112.69,621.9)	(111.84,557.08)
Federative Republic of Brazil	8.62	10.44	7.19	171.32	211.97	138.12
	(2.84,14.8)	(3.47,17.62)	(2.25,12.44)	(62.47,275.12)	(76.8,341.71)	(49.36,226.69)
French Republic	3.88	4.5	3.35	82.72	101.89	66.24
	(1.16,6.71)	(1.43,7.78)	(0.97,5.93)	(28.78,135.74)	(36.5,163.19)	(22.25,110.96)
Gabonese Republic	12.29	12.13	12.1	256.22	252.71	253.74
	(3.42,22.53)	(3.5,22.29)	(3.36,22.83)	(76.98,438.32)	(80.06,444.98)	(81,454.52)
Georgia	22.21	27.93	18.75	442.42	554.81	363.29
	(6.78,39.66)	(8.49,50.23)	(5.51,33.7)	(149.96,740.06)	(189.13,920.93)	(122.21,615.44)
Grand Duchy of Luxembourg	4.36	5.35	3.62	78.25	96.26	63.35
	(1.29,7.62)	(1.62,9.26)	(1.03,6.51)	(26.56,129.41)	(32.58,159.3)	(21.27,105.92)
Greenland	9.06	9.97	8.01	177.99	203.2	149.17
	(2.65,16.53)	(2.99,18.06)	(2.28,14.97)	(60.32,297.72)	(68.79,341.05)	(49.1,252.92)
Grenada	12.82	13.25	12.18	229.68	241.66	219.05
	(3.8,22.84)	(3.81,23.97)	(3.62,21.35)	(74.51,394.39)	(76.78,416.5)	(72.6,369.67)

Guam	3.45 (1.21,5.95)	4.17 (1.39,7.21)	2.88 (1.04,5.03)	141.34 (54.37,224.2)	148.64 (57.84,238.88)	136.04 (52.21,218.83)
Hashemite Kingdom of Jordan	16.03 (4.95,28.48)	13 (4.14,23.23)	19.94 (5.95,35.94)	339.33 (120.04,562.77)	306.58 (111.59,511.1)	379.05 (128.55,633.69)
Hellenic Republic	7.64 (2.15,13.8)	6.9 (2.02,12.42)	8.41 (2.34,15.2)	128.36 (42.5,217.16)	132.32 (44.7,222.27)	125.7 (40.17,215.55)
Hungary	13.49 (4.58,22.28)	16.61 (5.87,27.24)	11.13 (3.58,18.77)	295.14 (111.32,459.6)	375.26 (143.96,585.82)	232.74 (84.53,369.12)
Independent State of Papua New Guinea	9.9 (2.74,19.27)	7.18 (1.91,15.4)	12.96 (3.69,24.14)	206.51 (65.15,380.77)	158.7 (49.21,319.78)	260.84 (85.54,458.7)
Independent State of Samoa	9.44 (2.91,17.5)	9.32 (2.96,16.31)	9.45 (2.69,17.57)	224.82 (79.09,394.98)	217.84 (78.35,380.75)	231.31 (79.62,408.95)
Ireland	3.59 (1.02,6.62)	3.77 (1.1,6.99)	3.34 (0.96,6.19)	64 (21.49,110.9)	69.91 (23.86,121.74)	57.89 (19.38,98.68)
Islamic Republic of Afghanistan	29.16 (9.38,53)	29 (8.38,56.07)	29.36 (9.85,57.14)	637.15 (226.4,1127.34)	624.9 (207.26,1147)	650.28 (230.87,1210.37)
Islamic Republic of Iran	14.75 (4.64,25.23)	14.01 (4.4,24.28)	15.68 (4.84,27.07)	309.04 (110.02,495.57)	312.15 (112.75,502.85)	307.44 (108.97,497.67)
Islamic Republic of Mauritania	16.28 (4.79,31.59)	17.15 (5.21,33.23)	15.44 (4.07,32.09)	336.39 (109.43,615.8)	357.81 (118.35,649.42)	315.86 (97.14,610.5)
Islamic Republic of Pakistan	11.45 (3.68,21.18)	10.43 (3.21,19.73)	12.56 (4.29,23.51)	246.03 (87.83,431.18)	229.97 (78.95,421.84)	263.42 (97.82,475.86)
Jamaica	11.07 (3.2,19.78)	10.48 (3.1,18.95)	11.41 (3.35,20.09)	196.1 (62.72,338.57)	189.83 (60.28,331.64)	200.03 (67.18,339.39)
Japan	3.68 (1.09,6.51)	4.92 (1.49,8.72)	2.66 (0.77,4.83)	97 (34.01,155.52)	130.14 (45.05,209.67)	67.99 (24.11,110.67)
Kingdom of Bahrain	16.14 (4.68,28.48)	15.47 (4.6,26.63)	16.83 (4.87,29.58)	277.59 (96.61,463.54)	265.51 (88.83,446.3)	294.29 (104.42,487.86)
Kingdom of Belgium	4.09 (1.21,7.2)	4.37 (1.32,7.69)	3.79 (1.11,6.73)	84.84 (28.96,141.53)	94.43 (31.54,157.52)	76.14 (25.79,125.3)
Kingdom of Bhutan	10.54 (3.02,19.1)	10.97 (3.21,20.35)	10.13 (2.86,18.64)	209.81 (68.43,363.23)	217.53 (70.36,388.17)	202.23 (64.98,349.26)
Kingdom of Cambodia	17.58 (5.26,32.48)	18.01 (5.45,32.99)	17.24 (4.84,32.11)	329.83 (105.52,583.8)	339.71 (110.25,592.37)	323.51 (101.72,574.84)
Kingdom of Denmark	5.47 (1.62,9.45)	6.03 (1.83,10.52)	4.93 (1.43,8.59)	102.6 (34.72,168.75)	117.17 (39.55,191.75)	89.01 (30.03,147.35)
Kingdom of Eswatini	13.68 (3.5,25.53)	11.84 (2.99,22.74)	13.96 (3.58,27.16)	275.97 (79.25,505.41)	262.21 (73.06,497.91)	272.91 (77.64,522.07)
Kingdom of Lesotho	14.44 (3.97,27.12)	11.69 (3.41,23.07)	14.88 (4.02,29)	285.1 (84.3,527.81)	254.89 (76.81,488.74)	288.31 (82.49,543.52)
Kingdom of Morocco	26.03 (7.66,46.54)	22.16 (6.18,39.73)	29.66 (9.25,55.7)	536.94 (176.72,921.81)	448.19 (137.85,772.83)	623.32 (213.48,1130.61)
Kingdom of Norway	4.12 (1.23,7.25)	4.29 (1.28,7.49)	3.88 (1.15,6.86)	91.89 (30.99,150.22)	104.9 (35.72,170.36)	79.18 (26.22,131.12)

Kingdom of Saudi Arabia	18.17 (5.24,31.67)	17.47 (5.14,31.03)	19 (5.62,33.97)	391.59 (126.8,642.88)	384.46 (125.69,651.47)	400.71 (133.61,657.29)
Kingdom of Spain	3.66 (1.07,6.58)	3.94 (1.22,6.97)	3.34 (0.97,6.09)	79.63 (26.99,131.81)	96.69 (32.75,156.44)	64.47 (21.83,107.89)
Kingdom of Sweden	4.45 (1.26,7.93)	4.7 (1.36,8.17)	4.17 (1.15,7.59)	93.27 (31.91,154.15)	105.66 (35.5,176.35)	81.5 (28.83,135.95)
Kingdom of Thailand	8 (2.63,14.15)	8.69 (2.89,15.83)	7.31 (2.34,13.28)	204.96 (79.3,333.99)	228.72 (91.4,379.8)	183 (69.18,291.89)
Kingdom of the Netherlands	5.97 (1.82,10.52)	6.12 (1.86,10.65)	5.72 (1.73,10.13)	109.69 (37.36,180.36)	117.72 (39.78,193.67)	101.14 (34.91,167.7)
Kingdom of Tonga	7.99 (2.39,14.16)	8.36 (2.58,14.62)	7.56 (2.19,13.73)	185.8 (64.52,307.19)	194.62 (70.47,320.96)	177.1 (63.15,294.52)
Kyrgyz Republic	16.4 (5.15,27.87)	22.16 (6.89,37.57)	12.5 (3.89,21.49)	411.58 (144.38,668.51)	542.33 (183.44,887.25)	311.69 (110.72,513.02)
Lao People's Democratic Republic	19.12 (5.47,34.21)	21.17 (5.91,38.36)	17.27 (4.93,31.44)	398.86 (135.84,678.14)	446.19 (141.14,773.98)	354.9 (121.77,599.94)
Lebanese Republic	8.27 (2.51,14.78)	8.82 (2.68,15.45)	7.82 (2.37,13.92)	186.15 (67.55,297.7)	207.84 (74.57,332.41)	168.12 (61.61,273.42)
Malaysia	13.46 (4.41,22.5)	12.27 (4.08,20.36)	14.91 (4.63,25.71)	313.39 (116.45,487.43)	311.65 (115.56,485.01)	317.25 (116.17,516.8)
Mongolia	6.76 (2.22,12.22)	9.12 (3.01,16.84)	5.16 (1.49,9.63)	188.57 (69.89,304.34)	236.85 (85.09,400.39)	153.73 (55.67,251.58)
Montenegro	17.82 (5.05,32.72)	18.86 (5.09,33.48)	17 (4.63,31.85)	290.88 (90.59,506.19)	316.31 (96.81,537.56)	270.15 (82.76,472.29)
New Zealand	5.14 (1.51,9.05)	4.78 (1.35,8.53)	5.29 (1.57,9.42)	94.45 (32.03,155.99)	95.97 (32.58,158.21)	91.5 (31.29,151.83)
North Macedonia	59.37 (17.33,106.07)	52.86 (15.67,93.28)	62.52 (18.12,113.47)	905.74 (284.45,1565.26)	867.54 (276.16,1477.04)	913.04 (284.41,1600.07)
Northern Mariana Islands	8.59 (2.66,15.26)	9.01 (2.81,16.1)	8.06 (2.47,14.3)	196.09 (69.17,331.74)	210.4 (72.38,355.86)	180.63 (63.66,304.04)
Palestine	23.96 (7.21,42.59)	25.57 (7.66,47.06)	23.24 (7.06,42.25)	431.55 (144.94,728.06)	451.64 (147.13,773.56)	418.79 (140.97,718.16)
People's Democratic Republic of Algeria	19.39 (5.54,35)	15.76 (4.6,29.62)	25.45 (6.8,48.06)	348.53 (118.61,603.95)	296.13 (97.34,523.11)	422.79 (142.83,756.39)
People's Republic of Bangladesh	12.78 (3.35,24.84)	12.1 (3.41,23.59)	13.61 (3.4,27.29)	233.03 (68.56,442.68)	234 (69.83,461.6)	233.85 (68.71,447.23)
People's Republic of China	15.93 (4.83,28.08)	20.96 (6.72,37.13)	12.36 (3.73,22.43)	335.59 (112.75,566.25)	416.3 (143.15,703.34)	268.95 (91.45,455.85)
Plurinational State of Bolivia	7.71 (2.16,14.1)	7.22 (2.07,13.46)	8.04 (2.3,15.07)	144.5 (47.2,251.6)	139.79 (45.11,244.77)	147.74 (48.41,264.38)
Portuguese Republic	7.89 (2.32,13.91)	8.61 (2.61,14.78)	7.24 (2.05,12.81)	125.69 (42.36,209.34)	146.93 (49.77,243.57)	107.73 (35.32,180.94)
Principality of Andorra	3.95	3.39	4.31	77.89	74.17	79.79

	(1.01,7.35)	(0.93,6.27)	(1.12,8.35)	(25.95,132.06)	(25.53,126.03)	(26.74,139.71)
Principality of Monaco	9.07	8.78	9.14	159.59	164.67	153.07
	(2.73,15.83)	(2.58,15.8)	(2.8,16.61)	(55.74,261.32)	(54.89,271.89)	(54,264.98)
Puerto Rico	2.5 (0.78,4.42)	2.86	2.2	56.98	63.13	51.86
		(0.89,4.97)	(0.64,3.91)	(20.87,92.14)	(23.09,102.34)	(18.81,83.27)
Republic of Albania	12.09	12.54	11.64	210.02	221.99	198.73
	(3.53,22.05)	(3.67,22.68)	(3.17,22.61)	(70.16,360.96)	(73.14,390.94)	(65.26,350.26)
Republic of Angola	13.1	12.92	13.09	274	267.76	276.65
	(3.51,23.75)	(3.73,24.57)	(3.42,23.94)	(85.14,481.63)	(86.77,499.49)	(83.05,474.12)
Republic of Armenia	11.3	12.18	10.45	251	294.87	214.71
	(3.29,20.09)	(3.68,21.72)	(2.95,18.75)	(82.05,420.92)	(97.16,495.15)	(69.11,362.5)
Republic of Austria	3.9 (1.17,6.75)	4.41	3.47	99.4	114.9	86.04
		(1.38,7.5)	(1.02,6.14)	(34.63,160.13)	(39.86,185.08)	(30,139.46)
Republic of Azerbaijan	10	10.83	9.43	228.71	240.75	220.02
	(2.84,18.56)	(3.11,20.68)	(2.61,18.73)	(75.76,396.72)	(78.19,433.57)	(72.74,399.94)
Republic of Belarus	20.2	24.47	17.14	432.54	555.18	344.96
	(6.19,35.12)	(7.68,42.29)	(5.12,30.74)	(143.65,709.73)	(183.71,926.02)	(114.44,575.79)
Republic of Benin	15.7	17.01	14.32	317.56	356.83	279.22
	(4.56,28.43)	(5.09,31.03)	(4.08,27.42)	(100.79,559.71)	(115.88,629.43)	(87.62,495.9)
Republic of Botswana	10.4	11.2	9.61	222.1	237.6	207.32
	(2.91,18.56)	(3.14,20.24)	(2.65,17.79)	(69.71,382.7)	(71.98,409.65)	(65.35,370.09)
Republic of Bulgaria	41.87	50.34	35.47	775.22	976.81	611.83
	(13.02,71.01)	(16.06,85.13)	(10.58,61.43)	(272.99,1271.21)	(348.59,1580.1)	(209.56,1015.05)
Republic of Burundi	9.64	8.43	10.74	203.34	177.67	228.91
	(2.81,19.08)	(2.29,16.86)	(3.14,22.71)	(66.71,376.97)	(55.08,336.36)	(74.99,436.57)
Republic of Cabo Verde	14.6	21.14	10.13	306.1	443.46	201.11
	(4.28,24.84)	(6.31,37.75)	(2.94,17.94)	(100.9,509.87)	(145.23,758.69)	(67.25,332.93)
Republic of Cameroon	9.95	11.93	8.11	215.42	263.22	169.56
	(2.67,19.47)	(3.22,24.19)	(2.07,15.89)	(60.63,406.22)	(75.24,509.68)	(48.87,317.58)
Republic of Chad	16.15	18.65	13.17	346.34	405.61	275.97
	(4.68,31.11)	(5.28,35.19)	(3.47,27.19)	(104.35,644.22)	(122.89,742.23)	(84.81,533.59)
Republic of Chile	5.77	6.68	5.1	118.48	141.32	100.31
	(1.74,10.23)	(2.06,11.67)	(1.46,9.15)	(41.46,194.01)	(49.18,228.61)	(34.98,168.06)
Republic of Colombia	3.99	4.49	3.58	85.45	94.22	78.36
	(1.23,7.03)	(1.38,7.87)	(1.1,6.37)	(30.29,140.87)	(32.25,158.09)	(27.96,128.3)
Republic of Costa Rica	4.33	4.47	4.18	84.69	86.63	82.89
	(1.34,7.59)	(1.36,7.93)	(1.28,7.36)	(30,138.38)	(30.18,144.34)	(29.93,134.09)
Republic of Croatia	13.32	13.81	12.55	240.04	272.8	209.62
	(4.05,23.63)	(4.31,24.31)	(3.71,22.2)	(81.36,399.1)	(94.3,458.51)	(69.07,348.95)
Republic of Cuba	8.63	9.32	7.96	168.07	187.82	149.79
	(2.61,15.06)	(2.81,16.49)	(2.35,14.12)	(57.5,282.48)	(64.43,318.35)	(50.4,250.96)
Republic of Cyprus	9.22	8.36	9.3	123.04	120.48	119.27
	(2.71,16.72)	(2.45,15.02)	(2.61,17.15)	(39.75,212.94)	(39.06,206.72)	(38.15,208.3)
Republic of C 么 te d'Ivoire	16.55	20.21	12.63	361.07	451.61	259.25

	(5.15,30.41)	(6.52,37.66)	(3.55,23.52)	(120.29,639.37)	(153.1,805.11)	(82.57,459.63)
Republic of Djibouti	10.24	11.41	8.99	225.39	242.4	204.86
	(2.93,20.06)	(3.45,23.79)	(2.47,17.96)	(72.15,419.17)	(78.89,478.46)	(62.75,387.72)
Republic of Ecuador	4.87	5.51	4.3 (1.3,7.81)	96.14	108.27	85.22
	(1.48,8.99)	(1.66,9.93)		(33.36,165.14)	(36.36,181.55)	(29.98,145.82)
Republic of El Salvador	4.46 (1.34,7.9)	4.84	4.19	94.24	103.41	87.57
		(1.52,8.59)	(1.28,7.48)	(31.66,154.76)	(36.23,174.41)	(30.01,144.08)
Republic of Equatorial Guinea	12.4	11.54	12.91	256.12	238.48	267.98
	(3.41,23.46)	(3.04,22.06)	(3.74,25.6)	(78.09,463.36)	(70.75,431.25)	(81.96,490.96)
Republic of Estonia	8.53	12.45	6.29	192.99	273.22	140.73
	(2.77,14.42)	(3.97,21.01)	(1.91,10.8)	(70.29,303.4)	(99.29,436.45)	(51.43,222.57)
Republic of Fiji	11.55	13.83	10.47	264.59	290.78	251.02
	(3.65,20.87)	(4.24,24.63)	(3.09,19.3)	(95.95,441.78)	(99.14,484.68)	(89.97,418.93)
Republic of Finland	5.27	5.84	4.7 (1.37,8.3)	114.17	133.36	96.7
	(1.58,9.27)	(1.83,10.06)		(39.12,186.94)	(45.71,218.15)	(32.9,159.58)
Republic of Ghana	27.06	34.39	21.5	591.44	774.23	445.21
	(7.82,47.8)	(10.3,60.42)	(6.3,39.88)	(189.66,1004.62)	(247.33,1295.65)	(149.63,785.36)
Republic of Guatemala	4.32	4.48	4.17	80.76	81.52	79.99
	(1.28,7.82)	(1.31,8.06)	(1.26,7.52)	(27.19,138.11)	(26.6,139.48)	(27.46,136.18)
Republic of Guinea	17.38	19.9	14.72	373.52	441.52	303.37
	(5.26,32.29)	(6.13,37.45)	(4.27,28.66)	(124.65,666.82)	(146.15,798.79)	(99.65,542.52)
Republic of Guinea-Bissau	22.65	27.58	18.42	491.13	622.39	376.97
	(7.11,40.83)	(8.66,50.3)	(5.23,35.77)	(162.18,853.66)	(210.66,1070.49)	(114.81,702.53)
Republic of Guyana	18	17.6	18.12	346.87	363.49	329.07
	(5.61,31.66)	(5.49,31.5)	(5.54,31.85)	(118.05,586.55)	(126.17,629.87)	(111.27,565.93)
Republic of Haiti	20.34	15.82	24.62	383.06	302.81	457.53
	(5.99,39.86)	(4.26,30.47)	(7.09,49.14)	(122.79,715.5)	(93.45,578.05)	(141.48,886.49)
Republic of Honduras	14.16	14.34	13.98	255.16	262.12	248.79
	(4.28,25.46)	(4.22,24.62)	(4.45,26.35)	(86.06,439.63)	(82.35,439.81)	(85.43,443.66)
Republic of Iceland	3.84	3.59	3.91	79.55	83.56	74.44
	(1.11,6.65)	(1.04,6.21)	(1.12,6.8)	(27.07,130.88)	(28.7,136.46)	(25.42,121.89)
Republic of India	7.34	7.82	6.92	157.55	168.32	147.79
	(2.39,13.13)	(2.47,14.22)	(2.19,12.46)	(56.61,266.86)	(57.98,297.56)	(51.21,254.75)
Republic of Indonesia	19.43	20.26	18.44	404.9	428.68	380.24
	(5.62,35.48)	(6.09,37.24)	(5.03,35.73)	(132.32,697.84)	(140.73,753.25)	(122.52,686.84)
Republic of Iraq	33.18	37.63	29.29	668.41	753.51	591.45
	(10.13,57.16)	(11.18,64.5)	(9.14,51.32)	(233.81,1116.89)	(255.68,1255.14)	(211.4,977.51)
Republic of Italy	5.16	5.5 (1.6,9.81)	4.82	85.99	96.94	76.32
	(1.53,9.24)		(1.4,8.71)	(28.77,146.16)	(32.6,164.87)	(25.18,128.28)
Republic of Kazakhstan	25.25	29.55	22.2	509.11	615.47	433.98
	(7.66,45.76)	(9.07,52.02)	(6.49,40.9)	(170.97,860.23)	(207.51,1039.49)	(144.22,737.68)
Republic of Kenya	7.24	6.75	7.5	152.79	139.99	161.74
	(2.13,13.98)	(1.94,12.87)	(2.12,15.17)	(48.84,274.76)	(43.01,256.51)	(50.22,302.71)
Republic of Kiribati	13.34	19.38	9.61	327.88	450.39	242.05

	(4.3,23.79)	(6.07,35.98)	(2.7,17.18)	(118.87,549.27)	(159.87,780.18)	(83.03,409.51)
Republic of Korea	6.22	7.47	5.26	135.12	168.1	107.42
	(1.72,11.43)	(2.18,13.92)	(1.4,9.78)	(46,227.49)	(56.71,281.48)	(37.25,178.66)
Republic of Latvia	26.36	30.1	23.3	492.37	619.21	400.09
	(8.26,46.22)	(9.61,51.73)	(7.02,40.66)	(169.57,809.11)	(211.42,1012.94)	(133.41,660.23)
Republic of Liberia	15.91	16.82	14.87	333.68	362.69	300.73
	(5.05,29.57)	(4.84,32.06)	(4.25,29.14)	(113.14,595.83)	(115.82,665.66)	(94.53,570.87)
Republic of Lithuania	17.15	21.8	14.04	353.82	475.54	268.7
	(5.37,28.83)	(7.09,36.41)	(4.24,23.91)	(127.1,559.27)	(174.53,744.9)	(93.48,434.92)
Republic of Madagascar	13.44	13.22	13.61	294.06	280.39	305.88
	(3.97,25.44)	(3.66,25.89)	(3.84,25.68)	(96.58,529.63)	(85.66,528.85)	(93.48,555.35)
Republic of Malawi	14.41	15.47	13.4	297.78	327.49	271.23
	(4.14,26.59)	(4.76,29.38)	(3.67,26.26)	(96,530.08)	(108.22,606.23)	(82.96,505.85)
Republic of Maldives	10.97	11.93	9.87	219.87	238.19	198.43
	(3.18,19.52)	(3.46,21.12)	(2.83,17.62)	(76.8,364.73)	(83.74,398)	(67.71,328.45)
Republic of Mali	11.21	10.75	11.71	236.66	229.51	244.04
	(3.04,22.16)	(2.73,20.23)	(3.12,25.01)	(72.93,443.87)	(64.45,411.85)	(81.74,496.93)
Republic of Malta	4.34	4.26	4.31	79.41	81.58	76.69
	(1.31,7.68)	(1.3,7.52)	(1.29,7.62)	(27.43,131.24)	(28.45,132.16)	(26.26,127.17)
Republic of Mauritius	10.35	12.55	8.56	244.77	295.2	201.7
	(3.4,17.63)	(4.11,21.29)	(2.81,14.92)	(91.29,384.07)	(109.05,462.89)	(75.55,320.05)
Republic of Moldova	13.43	16.22	11.42	311.92	389.43	255.49
	(4.16,23.39)	(5.11,28.09)	(3.46,20.42)	(108.74,512.53)	(136.09,642.27)	(87.36,426.54)
Republic of Mozambique	20.52	26.67	15.88	450.2	598.62	329.61
	(6.17,36.08)	(8.56,48.13)	(4.45,30.52)	(148.32,762.8)	(200.42,1056.36)	(102.38,596.26)
Republic of Namibia	15.82	16.51	14.99	303.65	328.34	281.15
	(4.66,30.64)	(5.01,31.06)	(4.06,29.3)	(96.02,554.25)	(106.4,602.77)	(85.41,525.88)
Republic of Nauru	21.78	21.32	22.08	525.74	514.44	536.17
	(7.06,39.48)	(6.96,38.46)	(6.38,41.19)	(192.23,878.98)	(185.67,881.53)	(184.43,908.13)
Republic of Nicaragua	4.5 (1.4,8.28)	4.79	4.24	93.7	98	90.04
		(1.48,8.86)	(1.33,7.93)	(33.33,160.27)	(33.28,172.28)	(32.56,152.32)
Republic of Niue	10.83	11.75	10.07	252.03	266.7	239.62
	(3.52,19.6)	(3.67,21.08)	(3.19,18.22)	(87.34,424.71)	(92.97,459.46)	(84.46,407.6)
Republic of Palau	14.6	13.77	15.83	340.78	355.69	325.44
	(4.39,26.14)	(4.35,24.43)	(4.27,28.93)	(119.67,561.15)	(125.27,585.7)	(109.36,560.84)
Republic of Panama	5.98	6.75	5.28	110.7	121.89	100.62
	(1.75,10.66)	(1.95,12.44)	(1.58,9.13)	(37.68,189.41)	(40.14,216.16)	(35.51,164)
Republic of Paraguay	10.93	12.78	9.37	197.98	236.58	163.49
	(3.36,19.54)	(4.04,23.51)	(2.8,16.58)	(69.48,340.74)	(84.22,413.02)	(52.71,276.31)
Republic of Peru	3.78 (1.2,6.97)	3.89	3.66	82.13	84.85	79.38
		(1.22,7.26)	(1.12,6.7)	(29.5,138.85)	(30.28,147.71)	(28.08,134.1)
Republic of Poland	11.97	13.56	10.36	236.47	290.8	188
	(3.6,20.95)	(4.25,23.05)	(3.04,18.45)	(84,389.21)	(104.89,471.35)	(64.74,310.91)
Republic of Rwanda	6.98	7.09	6.88	143.63	141.61	144.75

	(1.82,14.06)	(1.75,14.34)	(1.78,14.76)	(41.42,279.16)	(39.6,284.27)	(43.05,283.76)
Republic of San Marino	4.25 (1.2,8.37)	4.22	4.23	88.91	95.85 (32,171.3)	82.07
		(1.21,8.29)	(1.13,8.57)	(29.82,156.83)		(27.2,149.38)
Republic of Senegal	16.49	18.05	14.98	343.58	382.99	305.79
	(4.8,30.19)	(5.21,33.74)	(4.02,28.72)	(108.45,598.18)	(119.34,687.58)	(94.4,565.09)
Republic of Serbia	39.72	41.12	38.29	675.98	731.33	624.99
	(11.66,70.16)	(12.34,71.31)	(11.51,67.53)	(214.65,1134.42)	(237.36,1227.06)	(205.21,1058.76)
Republic of Seychelles	12.7	14.42	10.87	283.24	331.98	233.52
	(4.06,21.78)	(4.48,25.02)	(3.49,18.99)	(102.22,455.84)	(118.52,539.66)	(85.46,376.97)
Republic of Sierra Leone	17.4	18.79	15.79	380.73	424.62	331.97
	(4.98,32.35)	(5.5,34.54)	(4.27,31.01)	(120.97,654.51)	(135.05,738.02)	(100.12,603.94)
Republic of Singapore	1.92	1.78	1.98	70.03	75.24	64.02
	(0.56,3.39)	(0.54,3.11)	(0.58,3.51)	(25.02,110.88)	(27.67,119.71)	(23.11,101.45)
Republic of Slovenia	7.03	8.86	5.75	131.59	165.02	105.52
	(2.04,12.36)	(2.7,15.39)	(1.66,10.46)	(45.24,220.71)	(56.04,276.73)	(36.15,178.29)
Republic of South Africa	13.03	11.76	13.35	263.01	247.22	267.54
	(3.94,23.67)	(3.61,21.26)	(4,24.48)	(86.39,448.37)	(84.24,425.36)	(86.51,460.69)
Republic of South Sudan	8.08	7.86	8.32	177.01	169.84	184.29
	(2.16,15.42)	(2.07,15.82)	(2.22,16.28)	(54.57,320.86)	(49.02,323.18)	(54.21,335.14)
Republic of Sudan	19.68	17.97	21.71	429.71	392.81	471.69
	(5.76,36.25)	(5.11,33.78)	(6.44,43.23)	(138.67,752.2)	(125.59,685.38)	(159.2,924.08)
Republic of Suriname	11.73	12.3	11.06	233.94	258.74	210.16
	(3.56,20.23)	(3.87,21.55)	(3.33,19.39)	(78.24,386.97)	(90.32,430.38)	(71.4,352.16)
Republic of Tajikistan	17.61	18.01	17.28	373.46	373.62	373.97
	(4.94,30.96)	(4.69,32.46)	(4.85,31.56)	(116.09,621.91)	(109.92,645.81)	(117.72,639.43)
Republic of the Congo	14.78	12.56	16.5	308.66	260.86	349.26
	(4.35,26.53)	(3.74,23.29)	(4.66,31.05)	(95.75,538.54)	(83.99,453.13)	(107.52,622.07)
Republic of the Gambia	20.54	22.84	18.05	432.6	501.44	361
	(5.88,37.97)	(6.83,41.76)	(4.67,36.45)	(137.77,750.07)	(161.11,862.27)	(103.57,697.6)
Republic of the Marshall Islands	14.33	13.23	15.51	324.27	300.68	349.38
	(4.21,26.43)	(3.85,26.93)	(4.29,28.66)	(108.09,560.67)	(97.56,569.27)	(117.36,624.02)
Republic of the Niger	11.54	12.53	10.6	234.26	252.22	216.95
	(3.05,22.39)	(3.33,24.14)	(2.79,21.76)	(71.14,438.09)	(72.81,483.33)	(61.23,429.54)
Republic of the Philippines	12.72	12.81	12.56	292.57	310.08	275.44
	(4.16,22.24)	(4.02,22.71)	(4.15,22.28)	(106.97,482.68)	(108.22,521.39)	(101.71,448.1)
Republic of the Union of Myanmar	17.94	22.72	14.68	371.56	469.06	299.99
	(5.15,32.36)	(6.61,40.7)	(3.98,27.24)	(121.51,641.36)	(156.09,811.76)	(93.04,524.55)
Republic of Trinidad and Tobago	11.48	13.71	9.5	219.83	270.48	173.12
	(3.48,19.78)	(4.33,23.6)	(2.78,16.53)	(75.31,362.78)	(93.35,443.03)	(58.83,287.82)
Republic of Tunisia	16.49	18.54	14.84	323.22	358.14	292.88
	(4.89,30.3)	(5.62,34.72)	(3.93,29.01)	(108.46,558.05)	(118.32,637.63)	(97.38,543.67)
Republic of Turkey	11.89	11.34	12.14	225.5	226.43	222.23
	(3.41,22.16)	(3.33,21.1)	(3.39,23.03)	(74.08,387.71)	(74.76,387.94)	(71.48,389.38)
Republic of Uganda	6.63	6.65	6.51	147.99	144.87	148.48

	(1.7,13.16)	(1.66,12.81)	(1.54,13.21)	(42.95,270.48)	(38.52,272.96)	(42.44,273.84)
Republic of Uzbekistan	17.43	20.64	15.03	419.99	485.66	366.15
	(5.58,29.6)	(6.43,35.91)	(4.8,25.79)	(145.94,676.87)	(163.23,793.62)	(132.56,583.87)
Republic of Vanuatu	14.09	15.28	12.94	335.7	360.13	311.89
	(4.46,25.92)	(4.56,28.2)	(3.96,25)	(120.38,566.1)	(126.49,615.48)	(111.46,544.08)
Republic of Yemen	29	29.38	28.64	601.3	606.51	596.68
	(9.06,55.63)	(8.48,55.67)	(8.4,55.87)	(204.1,1069.11)	(190.73,1084.54)	(195.36,1116.55)
Republic of Zambia	11.01	10.12	11.6	227.57	208.74	241.97
	(2.94,21.64)	(2.74,20.01)	(3.05,22.7)	(66.56,439.7)	(62.32,407.98)	(70.3,445.46)
Republic of Zimbabwe	14.64	14.32	14.78	305.84	304.8	305.93
	(4.31,27.68)	(4.21,26.64)	(4.29,28.28)	(96.79,555.91)	(97.58,553.64)	(92.28,560.47)
Romania	26.18	29.81	23.3	475.11	570.89	398.26
	(7.96,45.88)	(9.18,51.89)	(6.95,41.17)	(155.76,793.44)	(187.36,949.39)	(129.76,673.03)
Russian Federation	30.96	35.1	27.5	609.7	754	501.81
	(10.34,51.32)	(11.7,57.38)	(8.55,46.59)	(222.89,962.69)	(281.27,1176.45)	(173.99,809.67)
Saint Kitts and Nevis	18.29	17.24	18.13	317.97	327.5	297.19
	(5.49,31.8)	(5.35,29.71)	(5.39,31.92)	(104.12,537.63)	(108.06,542.84)	(95.61,514.39)
Saint Lucia	11.26	10.11	11.87	189.07	174.82	198.32
	(3.24,20.36)	(2.85,18.63)	(3.47,21.18)	(59.27,324.94)	(53.62,305.69)	(63.32,334.18)
Saint Vincent and the Grenadines	10.72	9.34	11.71	188.68	167.45	206.88
	(3.16,19.06)	(2.71,16.79)	(3.46,20.94)	(60.99,320.39)	(53.41,290.43)	(68.32,347.19)
Slovak Republic	14.25	16.66	12.3	307.76	374	253.59
	(4.12,25.22)	(4.99,29.42)	(3.46,22.01)	(100.29,519.83)	(124.87,622.89)	(82.98,422.68)
Socialist Republic of Viet Nam	22.95	32.33	16.8	446.83	643.48	301.71
	(6.89,41.81)	(10.04,58.79)	(4.5,32.51)	(152.03,787.82)	(218.47,1142.95)	(95.67,540.77)
Solomon Islands	12.96	10.41	15.49	296.18	247.86	344.25
	(3.53,25.01)	(2.75,21.98)	(4.41,29.46)	(98.31,521.77)	(75.65,471.41)	(117.08,606.57)
State of Eritrea	10.48	10.09	10.57	222.82	212.39	227.51
	(2.96,19.85)	(2.61,20.29)	(2.92,20.56)	(67.77,401.75)	(60.45,410.87)	(71.46,421.8)
State of Israel	2.72	2.7	2.67	66.27	74.68	58.31
	(0.78,4.84)	(0.82,4.77)	(0.74,4.81)	(22.47,108.49)	(26.04,121.9)	(19.67,97.51)
State of Kuwait	7.49	9.25	5.04	178.54	213.07	130.56
	(2.34,13.06)	(2.86,16.17)	(1.54,8.72)	(65.18,286.83)	(75.05,346.94)	(49.62,205.13)
State of Libya	15.48	13.99	16.94	354.85	315.7	394.68
	(4.92,28.75)	(4.2,26.18)	(5.38,33.39)	(125.67,622.36)	(104.9,564.59)	(139.74,717.61)
State of Qatar	8.71	9.06	8.5	167.61	170.85	165.82
	(2.44,16.26)	(2.59,16.72)	(2.3,16.25)	(54.68,293.11)	(53.9,303.87)	(57.24,291.15)
Sultanate of Oman	16.52	20.89	13.56	346.29	402.4	300.66
	(4.89,29.52)	(6.18,37.21)	(3.98,24.06)	(116.15,575.04)	(128.99,678.9)	(103.75,486.53)
Swiss Confederation	3.34	3.66	3.04	66.99	77.91	57.06
	(0.99,5.89)	(1.08,6.32)	(0.87,5.5)	(23.02,108.9)	(26.45,127.87)	(19.42,93.27)
Syrian Arab Republic	18.37	17.27	21.31	373.78	374.28	389.57
	(5.92,34.19)	(5.6,31.63)	(6.49,39.55)	(131.08,648.39)	(130.59,652.89)	(132.03,686.52)
Taiwan (Province of China)	4.28	5.08	3.58	134.32	151.61	119.55

	(1.36,7.33)	(1.61,8.74)	(1.08,6.25)	(48.66,210.87)	(55.14,240.53)	(43.81,188.75)
Togolese Republic	18.95 (5.56,33.18)	25.38 (7.61,45.88)	14.46 (3.85,27.24)	406.72 (129.23,700.87)	554.28 (178.64,944.55)	294.5 (90.94,531.45)
Tokelau	10.77 (3.36,19.72)	10.02 (3.11,18.1)	11.4 (3.54,21.34)	246.34 (88.93,418.55)	218.51 (78.05,376.44)	272.41 (98.55,461.03)
Turkmenistan	25.13 (8.09,45.02)	29.44 (9.04,53.01)	22.14 (7.32,39.6)	625.27 (222.44,1072.52)	713.56 (253.61,1238.33)	557.57 (208.46,943.87)
Tuvalu	13.94 (4.33,25.66)	13.76 (4.19,25.01)	13.96 (4.15,25.79)	314.1 (107.6,539.59)	310.58 (107.46,546.51)	316.21 (102.6,544.85)
Ukraine	24.5 (8.3,42.43)	29.46 (10.69,52.05)	21.16 (6.21,38.69)	516.24 (204.5,844.35)	653.68 (267.87,1102.48)	417.48 (144.56,716.08)
Union of the Comoros	11.13 (3.4,19.81)	10.64 (3.09,19.81)	11.5 (3.56,21.8)	238.59 (79.02,398.91)	218.95 (70.69,396.1)	255.14 (85.41,445.67)
United Arab Emirates	18.46 (5.13,31.72)	11.25 (3.28,19.81)	53.2 (14.94,94.72)	363.68 (116.93,595.36)	244.06 (82.46,401.31)	876 (257.28,1510.62)
United Kingdom of Great Britain and Northern Ireland	4.16 (1.2,7.32)	4.26 (1.26,7.49)	3.99 (1.14,7.06)	84.5 (28.59,139.57)	90.89 (30.93,149.09)	77.82 (26.11,128.87)
United Mexican States	4.89 (1.46,8.43)	5.08 (1.6,8.9)	4.72 (1.44,8.13)	103.74 (37,166.91)	110.81 (39.91,180.75)	97.51 (34.37,157.73)
United Republic of Tanzania	12.6 (3.55,22.49)	13.5 (3.78,25.16)	11.83 (3.26,22.29)	263.86 (83.94,445.26)	275.4 (87.52,490.63)	253.16 (78.6,445.24)
United States of America	4.11 (1.19,7.34)	3.94 (1.22,6.98)	4.12 (1.17,7.44)	105.08 (36.77,172.19)	106.64 (37.99,174.23)	102.44 (35.5,168.14)
United States Virgin Islands	4.98 (1.51,8.97)	6.28 (1.89,11.2)	4.13 (1.2,7.54)	95.44 (33.02,161.83)	115.92 (40.8,192.62)	79.94 (27.23,138.43)

ASMR Age-Standardized Disability-Adjusted Life Year Rate, *ASDR* Age-Standardized Disability-Adjusted Life Year Rate, *UI* uncertainty interval

Table S6 AAPC of ASMR and ASDR in 204 countries and territories from 1990 to 2021

**Location**	**ASMR/100,000 persons（95%UI）**			**ASDR/100,000 persons（95%UI）**		
	**Both**	**Male**	**Female**	**Both**	**Male**	**Female**
American Samoa	-0.80 (-1.00 - - 0.70)	-1.10 (-1.30 - - 0.90)	-0.50 (-0.70 - - 0.30)	-0.80 (-0.90 - - 0.60)	-1.00 (-1.20 - - 0.80)	-0.40 (-0.60 - - 0.30)
Antigua and Barbuda	-1.30 (-2.00 - - 0.60)	-1.70 (-2.90 - - 0.50)	-1.10 (-1.80 - - 0.50)	-1.50 (-2.10 - - 0.80)	-1.70 (-2.40 - - 1.10)	-1.20 (-1.80 - - 0.70)
Arab Republic of Egypt	-0.70 (-1.30 - - 0.00)	-0.30 (-1.00 - 0.40)	-0.20 (-0.90 - 0.50)	-0.40 (-1.10 - 0.20)	-0.20 (-0.60 - 0.30)	-0.40 (-1.20 - 0.30)
Argentine Republic	-3.30 (-3.90 - - 2.70)	-3.10 (-3.80 - - 2.40)	-3.40 (-3.90 - - 2.80)	-2.80 (-3.20 - - 2.30)	-2.80 (-3.30 - - 2.20)	-2.80 (-3.20 - - 2.40)
Australia	-4.10 (-4.40 - - 3.80)	-4.30 (-4.60 - - 3.90)	-3.90 (-4.50 - - 3.30)	-3.30 (-3.60 - - 3.10)	-3.40 (-3.70 - - 3.20)	-3.40 (-3.60 - - 3.20)
Barbados	-1.60 (-2.50 - - 0.70)	-1.60 (-2.60 - - 0.60)	-1.50 (-2.10 - - 0.80)	-1.50 (-2.00 - - 1.00)	-1.60 (-2.50 - - 0.70)	-1.40 (-1.80 - - 1.00)
Belize	-0.10 (-1.10 -	-0.40 (-1.20 -	-0.10 (-1.30 -	-0.20 (-0.80 -	-0.30 (-1.10 -	-0.30 (-1.50 -

	0.80)	0.50)	1.10)	0.40)	0.50)	0.80)
Bermuda	-3.00 (-3.70 - -2.30)	-2.70 (-3.50 - -2.00)	-3.20 (-4.20 - -2.30)	-2.90 (-3.40 - -2.40)	-2.70 (-3.40 - -2.00)	-3.00 (-3.60 - -2.30)
Bolivarian Republic of Venezuela	-0.60 (-1.00 - -0.30)	-0.40 (-0.80 - -0.10)	-0.90 (-1.20 - -0.50)	-0.90 (-1.20 - -0.60)	-0.80 (-1.10 - -0.50)	-1.00 (-1.30 - -0.70)
Bosnia and Herzegovina	-1.10 (-1.40 - -0.80)	-0.90 (-1.40 - -0.40)	-1.20 (-1.50 - -1.00)	-1.20 (-1.50 - -0.90)	-1.00 (-1.50 - -0.40)	-1.40 (-1.60 - -1.10)
Brunei Darussalam	-2.30 (-2.70 - -1.90)	-2.10 (-2.90 - -1.30)	-2.30 (-2.80 - -1.90)	-2.40 (-2.90 - -1.90)	-2.30 (-2.80 - -1.80)	-2.40 (-2.60 - -2.10)
Burkina Faso	-0.00 (-0.10 - 0.10)	-0.10 (-0.30 - 0.00)	0.30 (0.10 - 0.50)	-0.10 (-0.20 - -0.00)	-0.10 (-0.20 - -0.00)	0.00 (-0.20 - 0.20)
Canada	-3.40 (-3.70 - -3.00)	-3.80 (-4.00 - -3.60)	-3.10 (-3.50 - -2.80)	-2.40 (-2.50 - -2.30)	-2.70 (-2.90 - -2.40)	-2.20 (-2.40 - -2.10)
Central African Republic	-0.30 (-0.40 - -0.20)	-0.30 (-0.40 - -0.20)	-0.40 (-0.50 - -0.30)	-0.40 (-0.50 - -0.40)	-0.30 (-0.40 - -0.20)	-0.50 (-0.60 - -0.40)
Commonwealth of Dominica	-0.50 (-0.60 - -0.40)	-0.80 (-0.90 - -0.70)	-0.50 (-0.60 - -0.30)	-0.50 (-0.60 - -0.40)	-0.70 (-0.80 - -0.50)	-0.50 (-0.70 - -0.40)
Commonwealth of the Bahamas	-1.20 (-2.00 - -0.40)	-1.40 (-2.30 - -0.40)	-1.00 (-2.40 - 0.40)	-1.30 (-2.00 - -0.60)	-1.40 (-1.90 - -0.90)	-1.20 (-2.10 - -0.20)
Cook Islands	-1.90 (-1.90 - -1.80)	-1.70 (-1.90 - -1.60)	-2.00 (-2.00 - -1.90)	-1.30 (-1.40 - -1.30)	-1.20 (-1.30 - -1.10)	-1.50 (-1.50 - -1.40)
Czech Republic	-5.30 (-5.80 - -4.80)	-5.50 (-6.00 - -5.10)	-5.30 (-5.80 - -4.80)	-4.80 (-5.20 - -4.40)	-5.00 (-5.60 - -4.40)	-4.80 (-5.20 - -4.40)
Democratic People's Republic of Korea	-0.20 (-0.20 - -0.10)	0.10 (0.00 - 0.10)	-0.40 (-0.40 - -0.30)	-0.00 (-0.10 - -0.00)	0.10 (0.10 - 0.20)	-0.30 (-0.30 - -0.20)
Democratic Republic of Sao Tome and Principe	0.70 (0.50 - 0.80)	0.40 (0.10 - 0.70)	0.60 (0.50 - 0.70)	0.60 (0.40 - 0.80)	0.70 (0.50 - 0.90)	0.40 (0.30 - 0.50)
Democratic Republic of the Congo	-0.30 (-0.40 - -0.20)	-0.20 (-0.40 - -0.10)	-0.50 (-0.70 - -0.40)	-0.40 (-0.50 - -0.30)	-0.40 (-0.50 - -0.20)	-0.60 (-0.80 - -0.40)
Democratic Republic of Timor-Leste	0.20 (0.00 - 0.40)	0.60 (0.50 - 0.80)	-0.10 (-0.30 - 0.00)	0.20 (0.00 - 0.30)	0.60 (0.50 - 0.80)	-0.30 (-0.40 - -0.10)
Democratic Socialist Republic of Sri Lanka	-1.20 (-1.90 - -0.60)	-1.20 (-2.00 - -0.40)	-1.30 (-1.80 - -0.70)	-1.20 (-2.00 - -0.50)	-1.20 (-2.20 - -0.30)	-1.10 (-1.60 - -0.60)
Dominican Republic	-0.40 (-0.90 - 0.10)	0.10 (-1.00 - 1.10)	-0.80 (-1.30 - -0.20)	-0.10 (-0.60 - 0.40)	0.30 (-0.70 - 1.20)	-0.40 (-0.60 - -0.10)
Eastern Republic of Uruguay	-2.60 (-3.00 - -2.20)	-2.50 (-3.00 - -2.00)	-2.60 (-2.90 - -2.20)	-2.50 (-2.80 - -2.30)	-2.40 (-2.70 - -2.20)	-2.50 (-2.80 - -2.30)
Federal Democratic Republic of Ethiopia	-0.80 (-1.00 - -0.70)	-0.50 (-0.60 - -0.40)	-1.00 (-1.20 - -0.80)	-1.00 (-1.10 - -0.90)	-0.80 (-0.90 - -0.70)	-1.20 (-1.40 - -1.00)
Federal Democratic Republic of Nepal	-0.70 (-0.80 - -0.60)	0.30 (0.10 - 0.40)	-1.50 (-1.60 - -1.40)	-0.90 (-1.00 - -0.80)	0.10 (-0.10 - 0.20)	-1.80 (-1.90 - -1.60)
Federal Republic of Germany	-4.40 (-4.70 - -4.20)	-4.50 (-4.90 - -4.20)	-4.50 (-4.70 - -4.20)	-3.50 (-3.80 - -3.20)	-3.60 (-3.90 - -3.40)	-3.50 (-3.90 - -3.20)
Federal Republic of Nigeria	-0.80 (-0.90 - -	-0.70 (-0.80 - -	-0.80 (-1.00 - -	-0.80 (-0.90 - -	-0.60 (-0.70 - -	-0.80 (-0.90 - -

	0.70)	0.60)	0.70)	0.70)	0.50)	0.70)
Federal Republic of Somalia	-0.70 (-0.80 - -0.60)	-0.60 (-0.60 - -0.50)	-0.90 (-1.10 - -0.70)	-0.70 (-0.80 - -0.60)	-0.50 (-0.60 - -0.50)	-0.90 (-1.00 - -0.80)
Federated States of Micronesia	-0.60 (-0.60 - -0.50)	-0.50 (-0.50 - -0.40)	-0.70 (-0.70 - -0.60)	-0.50 (-0.60 - -0.50)	-0.40 (-0.50 - -0.40)	-0.60 (-0.70 - -0.60)
Federative Republic of Brazil	-3.10 (-3.40 - -2.80)	-2.90 (-3.40 - -2.50)	-3.20 (-3.50 - -3.00)	-3.00 (-3.40 - -2.60)	-2.90 (-3.30 - -2.50)	-3.10 (-3.40 - -2.80)
French Republic	-3.90 (-4.20 - -3.60)	-4.00 (-4.40 - -3.70)	-3.90 (-4.20 - -3.60)	-3.00 (-3.20 - -2.80)	-3.10 (-3.40 - -2.90)	-3.00 (-3.20 - -2.80)
Gabonese Republic	-0.20 (-0.40 - 0.00)	-0.20 (-0.30 - -0.00)	-0.20 (-0.40 - 0.00)	-0.30 (-0.50 - -0.10)	-0.30 (-0.40 - -0.10)	-0.30 (-0.60 - -0.10)
Georgia	0.70 (-0.60 - 1.90)	0.90 (-0.40 - 2.10)	0.40 (-0.90 - 1.80)	0.70 (-0.50 - 1.80)	1.00 (-0.20 - 2.20)	0.40 (-0.30 - 1.00)
Grand Duchy of Luxembourg	-5.70 (-6.40 - -5.00)	-5.50 (-6.30 - -4.80)	-6.00 (-6.30 - -5.70)	-5.40 (-6.00 - -4.90)	-5.30 (-5.90 - -4.60)	-5.60 (-5.90 - -5.20)
Greenland	-3.50 (-3.80 - -3.20)	-3.20 (-3.60 - -2.90)	-3.80 (-4.10 - -3.60)	-3.30 (-3.50 - -3.10)	-3.10 (-3.30 - -2.80)	-3.60 (-3.80 - -3.40)
Grenada	-2.00 (-2.60 - -1.50)	-1.90 (-3.20 - -0.60)	-1.90 (-2.60 - -1.20)	-2.40 (-2.80 - -1.90)	-2.20 (-3.10 - -1.20)	-2.30 (-2.90 - -1.80)
Guam	-3.90 (-4.90 - -2.80)	-2.40 (-3.80 - -0.90)	-5.10 (-6.40 - -3.80)	-1.80 (-2.40 - -1.10)	-1.30 (-1.70 - -0.80)	-2.20 (-2.90 - -1.40)
Hashemite Kingdom of Jordan	-2.40 (-2.90 - -1.80)	-2.00 (-2.20 - -1.80)	-2.20 (-3.40 - -1.10)	-2.20 (-2.70 - -1.60)	-1.80 (-2.10 - -1.60)	-2.50 (-3.30 - -1.70)
Hellenic Republic	-4.20 (-4.50 - -3.90)	-4.20 (-4.60 - -3.90)	-4.00 (-4.40 - -3.60)	-3.70 (-4.00 - -3.40)	-3.50 (-3.80 - -3.30)	-3.80 (-4.10 - -3.60)
Hungary	-3.70 (-4.20 - -3.10)	-3.70 (-4.60 - -2.80)	-3.90 (-4.70 - -3.10)	-3.40 (-3.90 - -3.00)	-3.50 (-4.00 - -2.90)	-3.40 (-3.90 - -3.00)
Independent State of Papua New Guinea	-0.40 (-0.50 - -0.30)	-0.20 (-0.40 - -0.00)	-0.50 (-0.60 - -0.40)	-0.50 (-0.60 - -0.40)	-0.30 (-0.40 - -0.10)	-0.60 (-0.70 - -0.50)
Independent State of Samoa	-0.60 (-0.60 - -0.50)	-0.70 (-0.80 - -0.70)	-0.50 (-0.50 - -0.40)	-0.40 (-0.50 - -0.40)	-0.50 (-0.60 - -0.50)	-0.30 (-0.30 - -0.20)
Ireland	-4.90 (-5.30 - -4.60)	-5.00 (-5.50 - -4.50)	-4.90 (-5.20 - -4.60)	-4.70 (-5.00 - -4.40)	-4.80 (-5.30 - -4.30)	-4.70 (-5.00 - -4.40)
Islamic Republic of Afghanistan	-0.20 (-0.30 - -0.10)	-0.10 (-0.20 - -0.00)	-0.20 (-0.30 - -0.10)	-0.20 (-0.30 - -0.10)	-0.10 (-0.20 - 0.00)	-0.30 (-0.40 - -0.20)
Islamic Republic of Iran	-2.10 (-2.20 - -1.90)	-2.00 (-2.20 - -1.80)	-2.00 (-2.20 - -1.80)	-2.10 (-2.20 - -1.90)	-1.90 (-2.10 - -1.70)	-2.20 (-2.30 - -2.00)
Islamic Republic of Mauritania	-0.60 (-0.80 - -0.40)	-0.80 (-0.90 - -0.60)	-0.40 (-0.50 - -0.30)	-0.80 (-0.90 - -0.70)	-1.00 (-1.20 - -0.90)	-0.60 (-0.70 - -0.50)
Islamic Republic of Pakistan	0.30 (0.20 - 0.30)	0.40 (0.30 - 0.50)	0.00 (-0.00 - 0.10)	0.20 (0.20 - 0.30)	0.40 (0.30 - 0.50)	0.00 (-0.10 - 0.10)
Jamaica	-0.90 (-1.50 - -0.40)	-0.60 (-1.60 - 0.40)	-1.10 (-2.10 - -0.20)	-1.00 (-1.50 - -0.40)	-0.70 (-1.70 - 0.20)	-1.20 (-2.10 - -0.20)
Japan	-4.40 (-4.90 - -	-4.00 (-4.40 - -	-5.00 (-5.70 - -	-3.30 (-3.60 - -	-2.90 (-3.20 - -	-3.80 (-4.10 - -

	4.00)	3.50)	4.40)	2.90)	2.70)	3.40)
Kingdom of Bahrain	-1.90 (-2.60 - -1.30)	-1.90 (-3.30 - -0.60)	-2.00 (-2.70 - -1.20)	-2.10 (-2.50 - -1.60)	-2.10 (-2.70 - -1.40)	-2.10 (-2.60 - -1.50)
Kingdom of Belgium	-4.40 (-4.60 - -4.30)	-4.70 (-5.10 - -4.40)	-4.30 (-4.50 - -4.10)	-3.80 (-3.90 - -3.70)	-4.00 (-4.30 - -3.80)	-3.70 (-3.80 - -3.60)
Kingdom of Bhutan	-0.30 (-0.40 - -0.20)	0.10 (-0.00 - 0.10)	-0.70 (-0.80 - -0.50)	-0.50 (-0.60 - -0.40)	-0.10 (-0.10 - 0.00)	-0.90 (-1.00 - -0.80)
Kingdom of Cambodia	-0.20 (-0.20 - -0.10)	-0.00 (-0.10 - 0.10)	-0.30 (-0.30 - -0.20)	-0.40 (-0.50 - -0.40)	-0.30 (-0.30 - -0.20)	-0.50 (-0.60 - -0.40)
Kingdom of Denmark	-3.40 (-3.80 - -3.00)	-3.50 (-3.90 - -3.00)	-3.30 (-3.70 - -3.00)	-3.30 (-3.60 - -3.00)	-3.40 (-3.90 - -3.00)	-3.20 (-3.50 - -2.90)
Kingdom of Eswatini	0.40 (0.10 - 0.70)	0.00 (-0.10 - 0.20)	0.50 (0.20 - 0.80)	0.50 (0.20 - 0.80)	0.40 (0.20 - 0.50)	0.40 (0.20 - 0.70)
Kingdom of Lesotho	1.70 (1.20 - 2.20)	1.10 (0.80 - 1.40)	1.80 (1.30 - 2.40)	1.80 (1.40 - 2.10)	1.40 (1.10 - 1.80)	1.80 (1.40 - 2.10)
Kingdom of Morocco	-0.00 (-0.20 - 0.10)	0.10 (-0.10 - 0.30)	-0.20 (-0.30 - -0.10)	-0.20 (-0.30 - -0.00)	-0.10 (-0.30 - 0.10)	-0.20 (-0.30 - -0.10)
Kingdom of Norway	-4.50 (-4.90 - -4.10)	-4.70 (-5.10 - -4.40)	-4.30 (-4.80 - -3.90)	-3.80 (-4.20 - -3.50)	-4.00 (-4.30 - -3.70)	-3.80 (-4.10 - -3.40)
Kingdom of Saudi Arabia	-0.80 (-0.90 - -0.60)	-0.80 (-1.00 - -0.70)	-0.70 (-0.90 - -0.40)	-0.60 (-0.70 - -0.50)	-0.60 (-0.70 - -0.50)	-0.70 (-0.90 - -0.40)
Kingdom of Spain	-5.20 (-5.30 - -5.00)	-5.20 (-5.50 - -4.90)	-5.30 (-5.40 - -5.10)	-4.20 (-4.50 - -4.00)	-4.00 (-4.30 - -3.70)	-4.60 (-4.90 - -4.30)
Kingdom of Sweden	-3.80 (-4.20 - -3.30)	-4.00 (-4.60 - -3.30)	-3.70 (-4.10 - -3.20)	-3.30 (-3.70 - -2.90)	-3.50 (-3.90 - -3.00)	-3.10 (-3.50 - -2.80)
Kingdom of Thailand	-1.70 (-2.10 - -1.20)	-1.50 (-2.10 - -1.00)	-1.80 (-2.20 - -1.40)	-1.30 (-1.70 - -1.00)	-1.20 (-1.50 - -0.80)	-1.60 (-1.80 - -1.30)
Kingdom of the Netherlands	-2.90 (-3.30 - -2.50)	-3.20 (-3.70 - -2.60)	-2.80 (-3.20 - -2.40)	-2.90 (-3.20 - -2.60)	-3.20 (-3.60 - -2.80)	-2.70 (-3.00 - -2.40)
Kingdom of Tonga	-0.10 (-0.40 - 0.30)	-0.40 (-0.80 - 0.10)	0.20 (-0.10 - 0.40)	-0.20 (-0.40 - 0.10)	-0.30 (-0.60 - 0.00)	-0.00 (-0.20 - 0.10)
Kyrgyz Republic	-1.90 (-2.30 - -1.50)	-1.40 (-2.20 - -0.70)	-2.50 (-2.80 - -2.10)	-1.50 (-1.90 - -1.10)	-1.00 (-1.70 - -0.30)	-2.10 (-2.40 - -1.70)
Lao People's Democratic Republic	-1.30 (-1.40 - -1.20)	-0.90 (-1.00 - -0.80)	-1.60 (-1.70 - -1.50)	-1.40 (-1.50 - -1.30)	-1.00 (-1.10 - -1.00)	-1.70 (-1.80 - -1.70)
Lebanese Republic	-3.10 (-3.30 - -2.80)	-3.00 (-3.30 - -2.60)	-3.20 (-3.50 - -2.90)	-2.80 (-3.00 - -2.60)	-2.60 (-2.90 - -2.30)	-3.00 (-3.30 - -2.60)
Malaysia	-0.60 (-1.00 - -0.30)	-0.90 (-1.10 - -0.70)	-0.50 (-1.00 - -0.10)	-0.80 (-1.00 - -0.50)	-0.70 (-0.80 - -0.60)	-0.70 (-1.10 - -0.40)
Mongolia	-0.00 (-0.50 - 0.50)	0.40 (-0.20 - 0.90)	-0.60 (-0.80 - -0.30)	0.10 (-0.10 - 0.30)	0.60 (0.20 - 1.00)	-0.40 (-0.70 - -0.20)
Montenegro	1.50 (0.80 - 2.30)	1.30 (1.00 - 1.70)	1.90 (1.00 - 2.80)	1.00 (0.60 - 1.30)	0.60 (0.40 - 0.80)	1.20 (0.60 - 1.70)
New Zealand	-3.10 (-3.40 - -	-3.30 (-3.70 - -	-2.90 (-3.30 - -	-3.00 (-3.20 - -	-3.20 (-3.30 - -	-2.90 (-3.00 - -

	2.90)	2.90)	2.50)	2.80)	3.00)	2.70)
North Macedonia	0.20 (-0.50 - 0.90)	-0.50 (-1.20 - 0.10)	0.40 (0.00 - 0.80)	-0.50 (-1.00 - 0.10)	-0.90 (-1.40 - - 0.40)	-0.30 (-0.60 - - 0.00)
Northern Mariana Islands	-1.40 (-2.10 - - 0.70)	-0.90 (-1.20 - - 0.60)	-2.30 (-3.00 - - 1.60)	-1.20 (-1.40 - - 0.90)	-0.70 (-0.90 - - 0.60)	-1.60 (-2.10 - - 1.20)
Palestine	-1.60 (-1.70 - - 1.40)	-1.60 (-1.90 - - 1.20)	-1.60 (-1.70 - - 1.40)	-1.60 (-1.90 - - 1.30)	-1.60 (-2.10 - - 1.20)	-1.60 (-1.80 - - 1.50)
People's Democratic Republic of Algeria	-0.90 (-1.10 - - 0.80)	-1.00 (-1.10 - - 0.90)	-0.80 (-1.10 - - 0.60)	-1.00 (-1.00 - - 0.90)	-1.10 (-1.10 - - 1.00)	-1.00 (-1.10 - - 0.80)
People's Republic of Bangladesh	-0.30 (-0.90 - 0.40)	0.00 (-0.70 - 0.70)	-0.30 (-1.40 - 0.80)	-0.40 (-0.90 - 0.10)	-0.30 (-0.90 - 0.30)	-0.50 (-1.20 - 0.20)
People's Republic of China	-0.40 (-0.70 - - 0.20)	-0.10 (-0.40 - 0.30)	-0.80 (-1.10 - - 0.50)	-0.50 (-0.60 - - 0.30)	-0.20 (-0.40 - 0.10)	-0.80 (-1.00 - - 0.60)
Plurinational State of Bolivia	-1.50 (-1.60 - - 1.40)	-1.50 (-1.70 - - 1.30)	-1.50 (-1.60 - - 1.30)	-1.70 (-1.80 - - 1.60)	-1.60 (-1.70 - - 1.50)	-1.70 (-1.80 - - 1.60)
Portuguese Republic	-5.70 (-6.10 - - 5.30)	-5.80 (-6.10 - - 5.50)	-5.60 (-6.00 - - 5.20)	-5.60 (-5.90 - - 5.20)	-5.60 (-5.80 - - 5.30)	-5.60 (-5.90 - - 5.20)
Principality of Andorra	-2.70 (-3.10 - - 2.20)	-2.90 (-3.60 - - 2.10)	-2.50 (-2.90 - - 2.20)	-2.40 (-2.70 - - 2.00)	-2.50 (-3.00 - - 2.00)	-2.10 (-2.40 - - 1.90)
Principality of Monaco	-3.10 (-3.20 - - 2.90)	-3.30 (-3.50 - - 3.20)	-2.90 (-3.00 - - 2.80)	-2.90 (-3.00 - - 2.80)	-3.10 (-3.20 - - 3.00)	-2.70 (-2.80 - - 2.60)
Puerto Rico	-3.70 (-4.70 - - 2.60)	-3.60 (-4.00 - - 3.20)	-3.70 (-4.50 - - 3.00)	-2.80 (-3.30 - - 2.30)	-2.80 (-3.30 - - 2.30)	-2.70 (-3.30 - - 2.00)
Republic of Albania	-0.90 (-1.50 - - 0.20)	-1.70 (-2.40 - - 1.00)	-0.40 (-1.10 - 0.40)	-1.10 (-1.70 - - 0.60)	-1.60 (-2.40 - - 0.70)	-0.70 (-1.30 - - 0.20)
Republic of Angola	-0.10 (-0.10 - 0.00)	-0.00 (-0.20 - 0.10)	-0.10 (-0.20 - 0.00)	-0.20 (-0.30 - - 0.10)	-0.20 (-0.40 - 0.10)	-0.30 (-0.40 - - 0.20)
Republic of Armenia	-1.10 (-2.40 - 0.20)	-1.00 (-2.10 - 0.10)	-1.10 (-2.10 - 0.00)	-1.10 (-1.90 - - 0.20)	-0.70 (-1.40 - - 0.00)	-1.30 (-2.00 - - 0.50)
Republic of Austria	-5.10 (-5.50 - - 4.60)	-5.20 (-5.70 - - 4.70)	-5.10 (-5.60 - - 4.70)	-3.80 (-4.20 - - 3.50)	-4.00 (-4.30 - - 3.70)	-3.80 (-4.10 - - 3.50)
Republic of Azerbaijan	-0.60 (-1.10 - - 0.10)	-1.20 (-1.50 - - 0.80)	-0.30 (-1.00 - 0.40)	-0.80 (-1.10 - - 0.50)	-1.20 (-1.40 - - 1.00)	-0.50 (-0.90 - - 0.20)
Republic of Belarus	-1.30 (-1.70 - - 0.80)	-0.80 (-1.30 - - 0.20)	-1.60 (-2.10 - - 1.10)	-1.30 (-1.80 - - 0.80)	-0.90 (-1.50 - - 0.30)	-1.70 (-2.10 - - 1.30)
Republic of Benin	-0.30 (-0.50 - - 0.10)	-0.40 (-0.60 - - 0.20)	-0.20 (-0.40 - - 0.10)	-0.40 (-0.60 - - 0.20)	-0.40 (-0.60 - - 0.20)	-0.30 (-0.50 - - 0.10)
Republic of Botswana	-1.20 (-2.20 - - 0.30)	-1.00 (-1.40 - - 0.50)	-1.30 (-2.70 - 0.10)	-1.10 (-1.90 - - 0.30)	-0.90 (-1.20 - - 0.50)	-1.10 (-2.30 - 0.00)
Republic of Bulgaria	-0.50 (-1.30 - 0.30)	-0.30 (-1.20 - 0.70)	-0.70 (-1.50 - 0.10)	-0.40 (-1.30 - 0.50)	-0.10 (-1.00 - 0.90)	-0.60 (-1.50 - 0.20)
Republic of Burundi	-1.70 (-1.90 - - 1.50)	-1.50 (-1.70 - - 1.30)	-1.70 (-1.90 - - 1.50)	-1.80 (-2.00 - - 1.60)	-1.60 (-1.80 - - 1.50)	-1.80 (-2.00 - - 1.60)
Republic of Cabo Verde	1.00 (0.40 -	1.50 (0.30 -	0.50 (0.30 -	0.70 (0.40 -	1.10 (0.10 -	0.10 (-0.00 -

	1.70)	2.80)	0.70)	1.00)	2.00)	0.30)
Republic of Cameroon	0.60 (0.50 - 0.70)	0.70 (0.50 - 0.80)	0.50 (0.30 - 0.60)	0.60 (0.40 - 0.70)	0.70 (0.50 - 0.90)	0.30 (0.20 - 0.50)
Republic of Chad	0.70 (0.50 - 0.80)	0.70 (0.50 - 0.90)	0.50 (0.40 - 0.60)	0.60 (0.50 - 0.80)	0.60 (0.40 - 0.90)	0.40 (0.30 - 0.50)
Republic of Chile	-3.10 (-3.70 - -2.40)	-2.70 (-3.30 - -2.20)	-3.40 (-4.00 - -2.70)	-2.90 (-3.20 - -2.60)	-2.60 (-3.00 - -2.30)	-3.10 (-3.60 - -2.60)
Republic of Colombia	-3.10 (-3.60 - -2.60)	-2.60 (-3.30 - -1.90)	-3.40 (-4.00 - -2.80)	-2.90 (-3.40 - -2.50)	-2.60 (-3.20 - -2.00)	-3.20 (-3.60 - -2.70)
Republic of Costa Rica	-1.30 (-2.10 - -0.50)	-1.10 (-1.70 - -0.40)	-1.80 (-2.30 - -1.20)	-1.40 (-2.00 - -0.80)	-1.20 (-1.70 - -0.80)	-1.70 (-2.10 - -1.30)
Republic of Croatia	-3.50 (-3.70 - -3.30)	-4.00 (-4.20 - -3.70)	-3.30 (-3.60 - -3.10)	-3.40 (-3.60 - -3.20)	-3.60 (-3.80 - -3.40)	-3.40 (-3.60 - -3.10)
Republic of Cuba	-0.80 (-1.50 - -0.00)	-0.30 (-1.00 - 0.40)	-1.10 (-1.30 - -1.00)	-0.60 (-1.20 - -0.10)	-0.10 (-0.70 - 0.40)	-1.10 (-1.80 - -0.50)
Republic of Cyprus	-4.10 (-5.00 - -3.20)	-4.40 (-5.00 - -3.70)	-4.20 (-4.80 - -3.60)	-4.10 (-4.80 - -3.40)	-4.20 (-4.70 - -3.60)	-4.30 (-4.80 - -3.80)
Republic of C 么 te d'Ivoire	0.00 (-0.20 - 0.30)	-0.00 (-0.20 - 0.10)	0.10 (-0.10 - 0.30)	-0.10 (-0.20 - 0.10)	-0.10 (-0.20 - 0.10)	0.00 (-0.30 - 0.30)
Republic of Djibouti	-0.10 (-0.20 - 0.10)	0.20 (0.10 - 0.30)	-0.40 (-0.50 - -0.30)	-0.10 (-0.20 - -0.00)	0.20 (0.10 - 0.30)	-0.40 (-0.50 - -0.40)
Republic of Ecuador	-2.20 (-3.10 - -1.20)	-1.90 (-2.80 - -0.90)	-2.50 (-3.50 - -1.60)	-2.40 (-3.20 - -1.60)	-2.20 (-3.00 - -1.30)	-2.60 (-3.40 - -1.90)
Republic of El Salvador	-1.40 (-2.10 - -0.60)	-1.30 (-2.30 - -0.30)	-1.20 (-2.20 - -0.20)	-1.40 (-2.10 - -0.80)	-1.30 (-2.50 - -0.20)	-1.40 (-2.10 - -0.80)
Republic of Equatorial Guinea	-0.50 (-0.70 - -0.30)	-0.80 (-1.00 - -0.50)	-0.30 (-0.70 - 0.10)	-0.60 (-0.80 - -0.50)	-0.90 (-1.20 - -0.70)	-0.40 (-0.80 - -0.10)
Republic of Estonia	-5.20 (-5.90 - -4.40)	-4.40 (-5.10 - -3.60)	-5.80 (-6.90 - -4.70)	-4.60 (-5.30 - -4.00)	-4.20 (-4.80 - -3.60)	-5.20 (-6.00 - -4.30)
Republic of Fiji	-0.30 (-0.50 - 0.00)	-0.40 (-0.70 - -0.10)	0.00 (-0.20 - 0.20)	-0.40 (-0.60 - -0.30)	-0.50 (-0.90 - -0.10)	-0.30 (-0.50 - -0.10)
Republic of Finland	-4.00 (-4.30 - -3.60)	-4.20 (-4.30 - -4.10)	-4.10 (-4.60 - -3.50)	-3.70 (-3.90 - -3.60)	-3.90 (-4.00 - -3.80)	-3.60 (-4.00 - -3.10)
Republic of Ghana	0.30 (0.20 - 0.40)	0.60 (0.50 - 0.70)	0.10 (-0.00 - 0.30)	0.20 (0.10 - 0.30)	0.60 (0.40 - 0.70)	-0.10 (-0.20 - 0.00)
Republic of Guatemala	-1.50 (-2.20 - -0.80)	-1.20 (-2.20 - -0.20)	-1.60 (-2.40 - -0.80)	-1.40 (-2.30 - -0.60)	-1.10 (-2.10 - -0.20)	-1.70 (-2.50 - -0.90)
Republic of Guinea	0.60 (0.50 - 0.70)	0.80 (0.70 - 0.90)	0.30 (0.20 - 0.30)	0.50 (0.40 - 0.60)	0.80 (0.70 - 1.00)	0.10 (0.00 - 0.20)
Republic of Guinea-Bissau	0.10 (0.00 - 0.20)	0.10 (-0.00 - 0.10)	0.40 (0.30 - 0.50)	0.00 (-0.10 - 0.10)	-0.00 (-0.10 - 0.00)	0.20 (0.10 - 0.20)
Republic of Guyana	-1.50 (-2.30 - -0.70)	-1.70 (-2.60 - -0.80)	-1.30 (-2.60 - -0.10)	-1.70 (-2.30 - -1.10)	-1.90 (-2.60 - -1.10)	-1.50 (-2.40 - -0.60)
Republic of Haiti	-0.90 (-1.00 - -	-0.80 (-0.80 - -	-0.80 (-0.90 - -	-0.90 (-1.00 - -	-0.80 (-0.90 - -	-0.90 (-1.10 - -

	0.70)	0.70)	0.70)	0.90)	0.70)	0.80)
Republic of Honduras	1.10 (0.70 - 1.40)	0.80 (0.60 - 0.90)	1.20 (0.40 - 2.00)	0.70 (0.30 - 1.00)	0.60 (0.40 - 0.70)	0.70 (0.10 - 1.30)
Republic of Iceland	-3.70 (-4.60 - - 2.70)	-4.20 (-5.30 - - 3.20)	-3.30 (-4.20 - - 2.40)	-3.30 (-3.60 - - 3.00)	-3.60 (-4.40 - - 2.80)	-3.10 (-3.60 - - 2.70)
Republic of India	-0.40 (-1.00 - 0.20)	-0.10 (-0.80 - 0.60)	-0.60 (-1.50 - 0.30)	-0.40 (-0.70 - - 0.20)	-0.20 (-0.50 - 0.10)	-0.70 (-1.20 - - 0.10)
Republic of Indonesia	1.00 (0.90 - 1.20)	1.30 (1.10 - 1.50)	0.80 (0.70 - 0.80)	0.70 (0.60 - 0.80)	1.00 (0.80 - 1.20)	0.40 (0.30 - 0.50)
Republic of Iraq	-0.10 (-0.30 - 0.10)	0.10 (-0.10 - 0.30)	-0.20 (-0.50 - - 0.00)	-0.50 (-0.60 - - 0.30)	-0.30 (-0.50 - - 0.10)	-0.70 (-0.90 - - 0.40)
Republic of Italy	-4.10 (-4.50 - - 3.70)	-4.30 (-4.70 - - 3.90)	-4.00 (-4.40 - - 3.60)	-3.90 (-4.20 - - 3.60)	-4.10 (-4.40 - - 3.80)	-3.90 (-4.20 - - 3.60)
Republic of Kazakhstan	-0.50 (-1.20 - 0.20)	-0.60 (-1.30 - 0.10)	-0.50 (-1.40 - 0.30)	-0.90 (-1.30 - - 0.40)	-0.90 (-1.60 - - 0.20)	-0.90 (-1.40 - - 0.40)
Republic of Kenya	0.50 (0.40 - 0.50)	0.90 (0.70 - 1.00)	0.10 (-0.10 - 0.30)	0.30 (0.20 - 0.40)	0.70 (0.60 - 0.80)	-0.10 (-0.20 - 0.00)
Republic of Kiribati	0.20 (0.20 - 0.30)	0.40 (0.30 - 0.40)	0.20 (0.10 - 0.30)	0.10 (0.00 - 0.10)	0.20 (0.20 - 0.30)	-0.10 (-0.10 - - 0.00)
Republic of Korea	-4.70 (-5.10 - - 4.40)	-4.70 (-5.10 - - 4.40)	-4.90 (-5.20 - - 4.50)	-4.40 (-4.60 - - 4.20)	-4.40 (-4.60 - - 4.20)	-4.50 (-4.80 - - 4.30)
Republic of Latvia	-1.70 (-2.70 - - 0.60)	-1.70 (-3.00 - - 0.50)	-1.80 (-2.60 - - 1.00)	-1.70 (-2.60 - - 0.90)	-1.70 (-3.00 - - 0.30)	-2.00 (-2.80 - - 1.30)
Republic of Liberia	0.10 (-0.20 - 0.40)	0.10 (-0.00 - 0.30)	0.10 (-0.20 - 0.40)	0.10 (-0.10 - 0.20)	0.10 (-0.10 - 0.30)	-0.00 (-0.30 - 0.20)
Republic of Lithuania	-1.40 (-1.90 - - 0.90)	-0.90 (-1.60 - - 0.10)	-1.80 (-2.60 - - 1.00)	-1.50 (-2.30 - - 0.80)	-0.90 (-1.50 - - 0.20)	-2.10 (-2.80 - - 1.40)
Republic of Madagascar	-0.20 (-0.30 - - 0.10)	-0.30 (-0.40 - - 0.20)	-0.10 (-0.30 - 0.00)	-0.30 (-0.40 - - 0.10)	-0.30 (-0.50 - - 0.20)	-0.20 (-0.30 - - 0.10)
Republic of Malawi	0.50 (0.30 - 0.70)	0.90 (0.80 - 1.10)	0.20 (-0.00 - 0.40)	0.40 (0.30 - 0.50)	0.90 (0.80 - 1.00)	-0.00 (-0.20 - 0.10)
Republic of Maldives	-2.50 (-2.60 - - 2.30)	-2.00 (-2.30 - - 1.80)	-3.00 (-3.40 - - 2.70)	-2.80 (-3.00 - - 2.70)	-2.30 (-2.50 - - 2.20)	-3.30 (-3.60 - - 3.10)
Republic of Mali	0.00 (-0.20 - 0.20)	0.40 (0.20 - 0.60)	-0.20 (-0.40 - - 0.10)	-0.00 (-0.20 - 0.10)	0.30 (0.20 - 0.50)	-0.40 (-0.50 - - 0.20)
Republic of Malta	-4.80 (-5.30 - - 4.30)	-5.00 (-5.50 - - 4.50)	-4.60 (-5.20 - - 4.00)	-4.60 (-4.90 - - 4.20)	-4.70 (-5.10 - - 4.20)	-4.50 (-4.90 - - 4.00)
Republic of Mauritius	-3.40 (-4.40 - - 2.40)	-3.50 (-4.80 - - 2.10)	-3.30 (-4.40 - - 2.20)	-3.20 (-4.00 - - 2.50)	-3.40 (-4.50 - - 2.40)	-3.00 (-3.70 - - 2.20)
Republic of Moldova	-1.50 (-2.60 - - 0.50)	-1.00 (-2.00 - - 0.10)	-2.00 (-3.00 - - 1.00)	-1.00 (-2.00 - 0.00)	-0.50 (-1.60 - 0.70)	-1.30 (-2.30 - - 0.30)
Republic of Mozambique	0.80 (0.70 - 0.90)	1.30 (1.20 - 1.50)	0.30 (0.20 - 0.50)	0.90 (0.70 - 1.00)	1.50 (1.30 - 1.60)	0.20 (0.10 - 0.40)
Republic of Namibia	-0.30 (-0.50 - -	-0.20 (-0.30 - -	-0.30 (-0.60 -	-0.30 (-0.60 - -	-0.20 (-0.40 - -	-0.50 (-0.70 - -

	0.10)	0.10)	0.00)	0.10)	0.00)	0.30)
Republic of Nauru	-0.20 (-0.30 - -0.20)	-0.30 (-0.40 - -0.20)	-0.20 (-0.40 - -0.10)	-0.20 (-0.30 - -0.10)	-0.30 (-0.40 - -0.20)	-0.20 (-0.40 - 0.00)
Republic of Nicaragua	-1.70 (-2.10 - -1.20)	-2.00 (-2.30 - -1.80)	-1.50 (-2.00 - -0.90)	-1.60 (-2.10 - -1.20)	-1.70 (-2.00 - -1.50)	-1.60 (-2.00 - -1.20)
Republic of Niue	-0.70 (-0.70 - -0.60)	-0.60 (-0.60 - -0.60)	-0.70 (-0.80 - -0.60)	-0.60 (-0.70 - -0.50)	-0.60 (-0.60 - -0.50)	-0.60 (-0.70 - -0.50)
Republic of Palau	-0.40 (-0.60 - -0.30)	-1.00 (-1.30 - -0.70)	0.20 (0.00 - 0.40)	-0.40 (-0.60 - -0.30)	-0.60 (-0.80 - -0.40)	-0.20 (-0.30 - -0.10)
Republic of Panama	-1.40 (-2.00 - -0.70)	-1.00 (-1.80 - -0.30)	-1.70 (-2.40 - -0.90)	-1.50 (-1.90 - -1.00)	-1.30 (-2.00 - -0.50)	-1.70 (-2.10 - -1.30)
Republic of Paraguay	-1.10 (-1.60 - -0.60)	-0.90 (-1.50 - -0.40)	-1.40 (-1.90 - -0.90)	-1.00 (-1.30 - -0.70)	-0.80 (-1.40 - -0.30)	-1.30 (-1.60 - -0.90)
Republic of Peru	-1.50 (-2.80 - -0.20)	-1.50 (-2.70 - -0.20)	-1.60 (-2.90 - -0.30)	-1.30 (-2.30 - -0.30)	-1.30 (-2.40 - -0.10)	-1.40 (-2.40 - -0.30)
Republic of Poland	-3.60 (-4.00 - -3.20)	-3.40 (-3.70 - -3.00)	-3.80 (-4.20 - -3.40)	-3.30 (-3.70 - -2.90)	-3.00 (-3.40 - -2.70)	-3.60 (-4.00 - -3.20)
Republic of Rwanda	-2.00 (-2.10 - -1.80)	-1.40 (-1.50 - -1.30)	-2.30 (-2.50 - -2.10)	-2.10 (-2.30 - -2.00)	-1.70 (-1.80 - -1.60)	-2.50 (-2.60 - -2.30)
Republic of San Marino	-4.20 (-4.70 - -3.60)	-4.80 (-5.50 - -4.20)	-3.70 (-4.50 - -2.90)	-3.40 (-3.70 - -3.10)	-3.70 (-3.90 - -3.60)	-3.10 (-3.50 - -2.70)
Republic of Senegal	-0.10 (-0.30 - 0.20)	-0.20 (-0.40 - -0.00)	-0.10 (-0.30 - 0.20)	-0.40 (-0.60 - -0.10)	-0.40 (-0.70 - -0.20)	-0.30 (-0.50 - -0.00)
Republic of Serbia	-1.90 (-2.10 - -1.60)	-1.80 (-2.30 - -1.30)	-1.90 (-2.20 - -1.50)	-1.70 (-2.00 - -1.50)	-1.70 (-1.90 - -1.40)	-1.80 (-2.20 - -1.50)
Republic of Seychelles	-1.40 (-1.90 - -0.90)	-1.90 (-3.20 - -0.60)	-0.80 (-1.30 - -0.30)	-1.30 (-1.90 - -0.70)	-1.80 (-2.40 - -1.10)	-0.90 (-1.30 - -0.50)
Republic of Sierra Leone	-0.00 (-0.20 - 0.10)	-0.30 (-0.50 - -0.10)	0.40 (0.20 - 0.60)	-0.10 (-0.20 - 0.10)	-0.30 (-0.50 - -0.10)	0.40 (0.30 - 0.40)
Republic of Singapore	-6.90 (-7.80 - -5.90)	-6.90 (-7.80 - -6.00)	-6.90 (-8.20 - -5.50)	-5.40 (-5.90 - -4.80)	-5.40 (-6.00 - -4.70)	-5.60 (-6.30 - -4.80)
Republic of Slovenia	-4.20 (-5.00 - -3.50)	-3.90 (-5.10 - -2.60)	-4.50 (-5.40 - -3.60)	-4.10 (-4.50 - -3.70)	-4.10 (-4.60 - -3.70)	-4.10 (-4.60 - -3.70)
Republic of South Africa	0.90 (0.40 - 1.50)	0.50 (0.00 - 1.00)	1.10 (0.40 - 1.70)	0.40 (-0.10 - 0.90)	0.20 (-0.30 - 0.60)	0.40 (-0.20 - 0.90)
Republic of South Sudan	-0.50 (-0.70 - -0.40)	-0.50 (-0.70 - -0.40)	-0.50 (-0.70 - -0.40)	-0.50 (-0.60 - -0.40)	-0.50 (-0.60 - -0.40)	-0.50 (-0.60 - -0.40)
Republic of Sudan	-0.60 (-0.70 - -0.50)	-0.50 (-0.50 - -0.40)	-0.70 (-0.70 - -0.70)	-0.60 (-0.70 - -0.50)	-0.40 (-0.50 - -0.30)	-0.70 (-0.80 - -0.70)
Republic of Suriname	-0.70 (-1.30 - -0.20)	-0.30 (-1.40 - 0.80)	-1.00 (-1.50 - -0.60)	-0.70 (-1.20 - -0.30)	-0.40 (-1.10 - 0.40)	-1.10 (-1.50 - -0.70)
Republic of Tajikistan	-0.80 (-1.20 - -0.30)	-1.20 (-1.70 - -0.70)	-0.40 (-0.80 - -0.10)	-1.00 (-1.40 - -0.60)	-1.40 (-1.80 - -1.00)	-0.70 (-0.90 - -0.40)
Republic of the Congo	-0.60 (-0.70 - -	-0.90 (-0.90 - -	-0.30 (-0.50 - -	-0.60 (-0.80 - -	-1.00 (-1.10 - -	-0.40 (-0.60 - -

	0.40)	0.80)	0.10)	0.50)	0.80)	0.10)
Republic of the Gambia	0.60 (0.20 - 1.00)	0.50 (0.20 - 0.70)	0.80 (0.50 - 1.00)	0.40 (0.10 - 0.70)	0.40 (0.00 - 0.70)	0.60 (0.10 - 1.00)
Republic of the Marshall Islands	-0.40 (-0.50 - -0.30)	-0.60 (-0.70 - -0.40)	-0.10 (-0.10 - -0.00)	-0.30 (-0.40 - -0.20)	-0.50 (-0.70 - -0.30)	-0.00 (-0.10 - 0.00)
Republic of the Niger	0.10 (0.00 - 0.20)	0.20 (0.10 - 0.40)	0.10 (-0.10 - 0.20)	-0.00 (-0.10 - 0.00)	0.10 (-0.00 - 0.20)	-0.20 (-0.30 - -0.00)
Republic of the Philippines	-0.40 (-0.80 - -0.00)	-0.00 (-0.50 - 0.40)	-0.90 (-1.50 - -0.40)	0.10 (-0.20 - 0.40)	0.30 (0.00 - 0.60)	-0.20 (-0.60 - 0.10)
Republic of the Union of Myanmar	-1.10 (-1.10 - -1.00)	-0.60 (-0.70 - -0.50)	-1.40 (-1.50 - -1.30)	-1.20 (-1.30 - -1.20)	-0.80 (-0.90 - -0.70)	-1.60 (-1.60 - -1.50)
Republic of Trinidad and Tobago	-2.20 (-2.40 - -2.00)	-2.00 (-2.30 - -1.60)	-2.40 (-2.60 - -2.20)	-2.00 (-2.20 - -1.80)	-1.90 (-2.20 - -1.50)	-2.10 (-2.30 - -1.90)
Republic of Tunisia	-0.90 (-1.10 - -0.60)	-0.70 (-1.00 - -0.30)	-1.10 (-1.20 - -1.00)	-0.80 (-0.90 - -0.70)	-0.50 (-0.60 - -0.30)	-1.00 (-1.10 - -0.90)
Republic of Turkey	-1.90 (-2.20 - -1.50)	-2.30 (-2.60 - -2.10)	-1.50 (-2.80 - -0.20)	-2.20 (-2.60 - -1.80)	-2.50 (-2.80 - -2.30)	-1.80 (-2.30 - -1.30)
Republic of Uganda	-0.70 (-0.90 - -0.60)	-0.60 (-0.80 - -0.50)	-0.90 (-1.00 - -0.70)	-0.70 (-0.80 - -0.60)	-0.60 (-0.80 - -0.50)	-0.80 (-0.90 - -0.60)
Republic of Uzbekistan	-0.10 (-0.90 - 0.80)	-0.00 (-1.00 - 1.00)	-0.20 (-1.20 - 0.80)	-0.40 (-1.20 - 0.50)	-0.30 (-1.20 - 0.50)	-0.50 (-1.40 - 0.30)
Republic of Vanuatu	-0.50 (-0.70 - -0.40)	-0.40 (-0.60 - -0.30)	-0.60 (-0.70 - -0.50)	-0.40 (-0.60 - -0.30)	-0.30 (-0.50 - -0.20)	-0.40 (-0.50 - -0.30)
Republic of Yemen	-0.10 (-0.30 - -0.00)	0.00 (-0.20 - 0.20)	-0.30 (-0.40 - -0.20)	-0.20 (-0.40 - -0.00)	-0.00 (-0.30 - 0.20)	-0.40 (-0.60 - -0.20)
Republic of Zambia	0.50 (0.40 - 0.70)	0.80 (0.70 - 1.00)	0.20 (-0.10 - 0.50)	0.40 (0.30 - 0.60)	0.80 (0.70 - 0.90)	0.00 (-0.20 - 0.20)
Republic of Zimbabwe	1.30 (0.90 - 1.70)	1.00 (0.80 - 1.20)	1.30 (0.40 - 2.30)	1.40 (1.00 - 1.80)	1.20 (1.00 - 1.50)	1.40 (1.00 - 1.80)
Romania	-1.90 (-2.50 - -1.40)	-1.60 (-2.10 - -1.00)	-2.20 (-2.70 - -1.60)	-1.70 (-2.20 - -1.30)	-1.40 (-1.90 - -0.90)	-2.00 (-2.50 - -1.50)
Russian Federation	-1.90 (-2.60 - -1.10)	-1.80 (-2.80 - -0.90)	-2.00 (-2.70 - -1.30)	-1.70 (-2.40 - -0.90)	-1.50 (-2.30 - -0.70)	-2.00 (-2.80 - -1.20)
Saint Kitts and Nevis	-1.90 (-2.70 - -1.10)	-2.20 (-2.90 - -1.40)	-1.90 (-3.00 - -0.80)	-1.90 (-2.40 - -1.50)	-1.90 (-2.40 - -1.30)	-2.30 (-3.20 - -1.40)
Saint Lucia	-2.90 (-3.40 - -2.40)	-2.00 (-2.90 - -1.10)	-2.90 (-4.10 - -1.70)	-2.70 (-3.60 - -1.90)	-2.10 (-2.90 - -1.40)	-2.80 (-3.80 - -1.70)
Saint Vincent and the Grenadines	-1.40 (-1.90 - -0.90)	-1.20 (-1.50 - -0.80)	-1.50 (-2.30 - -0.80)	-1.50 (-1.80 - -1.10)	-1.00 (-1.10 - -0.80)	-1.50 (-2.10 - -0.90)
Slovak Republic	-2.40 (-2.80 - -1.90)	-2.30 (-2.60 - -2.10)	-2.40 (-2.60 - -2.10)	-2.30 (-2.70 - -1.90)	-2.30 (-2.60 - -2.00)	-2.30 (-2.50 - -2.00)
Socialist Republic of Viet Nam	0.30 (0.20 - 0.30)	0.60 (0.50 - 0.70)	-0.20 (-0.20 - -0.10)	0.20 (0.20 - 0.30)	0.60 (0.50 - 0.70)	-0.30 (-0.40 - -0.30)
Solomon Islands	-0.10 (-0.20 -	-0.10 (-0.20 -	-0.30 (-0.40 - -	-0.10 (-0.30 -	-0.10 (-0.30 -	-0.20 (-0.40 - -

	0.00)	0.10)	0.20)	0.10)	0.20)	0.10)
State of Eritrea	-0.40 (-0.50 - -0.30)	-0.00 (-0.20 - 0.10)	-0.60 (-0.60 - -0.50)	-0.60 (-0.70 - -0.50)	-0.30 (-0.40 - -0.20)	-0.70 (-0.80 - -0.60)
State of Israel	-4.30 (-5.40 - -3.20)	-4.30 (-5.50 - -3.20)	-4.40 (-4.80 - -4.10)	-3.40 (-3.90 - -3.00)	-3.30 (-3.80 - -2.80)	-3.60 (-4.00 - -3.10)
State of Kuwait	-1.00 (-3.00 - 1.00)	-0.20 (-3.00 - 2.60)	-2.90 (-4.60 - -1.10)	-1.10 (-2.70 - 0.60)	-0.40 (-2.60 - 1.90)	-2.60 (-3.50 - -1.70)
State of Libya	0.60 (0.40 - 0.90)	0.80 (0.30 - 1.30)	0.40 (0.00 - 0.90)	0.80 (0.60 - 1.00)	0.90 (0.40 - 1.40)	0.50 (0.10 - 0.90)
State of Qatar	-2.90 (-4.30 - -1.50)	-3.00 (-4.80 - -1.10)	-3.00 (-3.80 - -2.10)	-2.80 (-3.80 - -1.80)	-2.90 (-4.20 - -1.70)	-2.60 (-3.20 - -1.90)
Sultanate of Oman	-1.00 (-2.00 - -0.00)	-0.90 (-2.30 - 0.50)	-1.20 (-1.60 - -0.80)	-1.30 (-1.70 - -0.90)	-1.20 (-1.80 - -0.50)	-1.40 (-1.80 - -1.10)
Swiss Confederation	-4.30 (-4.50 - -4.10)	-4.60 (-4.70 - -4.40)	-4.20 (-4.40 - -4.10)	-3.70 (-3.80 - -3.60)	-3.90 (-4.10 - -3.80)	-3.60 (-3.80 - -3.50)
Syrian Arab Republic	-0.60 (-0.80 - -0.40)	-0.50 (-0.90 - -0.10)	-0.40 (-0.90 - 0.20)	-0.80 (-1.10 - -0.60)	-0.80 (-1.10 - -0.50)	-0.80 (-1.10 - -0.60)
Taiwan (Province of China)	-4.40 (-5.10 - -3.60)	-3.70 (-4.50 - -2.90)	-4.90 (-5.70 - -4.10)	-3.20 (-3.70 - -2.70)	-2.90 (-3.40 - -2.40)	-3.50 (-3.90 - -3.10)
Togolese Republic	0.10 (0.00 - 0.20)	0.50 (0.40 - 0.60)	-0.10 (-0.20 - 0.00)	0.10 (-0.00 - 0.10)	0.40 (0.30 - 0.50)	-0.30 (-0.30 - -0.20)
Tokelau	-1.10 (-1.20 - -1.10)	-1.10 (-1.10 - -1.00)	-1.10 (-1.20 - -1.10)	-0.90 (-0.90 - -0.90)	-0.90 (-0.90 - -0.80)	-0.90 (-0.90 - -0.90)
Turkmenistan	0.60 (-0.30 - 1.60)	0.80 (-0.20 - 1.70)	0.50 (-0.50 - 1.50)	0.60 (-0.20 - 1.40)	0.70 (-0.20 - 1.70)	0.50 (-0.30 - 1.20)
Tuvalu	-0.70 (-0.70 - -0.60)	-0.60 (-0.70 - -0.50)	-0.70 (-0.80 - -0.60)	-0.60 (-0.70 - -0.60)	-0.50 (-0.60 - -0.50)	-0.60 (-0.70 - -0.60)
Ukraine	-2.30 (-3.00 - -1.60)	-2.00 (-2.70 - -1.20)	-2.50 (-3.10 - -1.90)	-1.90 (-2.50 - -1.30)	-1.60 (-2.50 - -0.80)	-2.20 (-2.70 - -1.80)
Union of the Comoros	-1.00 (-1.10 - -0.90)	-0.80 (-0.90 - -0.70)	-1.10 (-1.20 - -1.00)	-1.00 (-1.10 - -0.90)	-0.90 (-1.10 - -0.80)	-1.10 (-1.20 - -1.00)
United Arab Emirates	-1.30 (-3.90 - 1.30)	-2.90 (-4.10 - -1.60)	2.10 (-0.10 - 4.30)	-1.70 (-3.40 - 0.10)	-2.90 (-3.70 - -2.10)	1.10 (-0.40 - 2.70)
United Kingdom of Great Britain and Northern Ireland	-4.70 (-5.20 - -4.20)	-4.90 (-5.30 - -4.50)	-4.60 (-5.10 - -4.10)	-4.20 (-4.50 - -3.90)	-4.30 (-4.50 - -4.10)	-4.10 (-4.40 - -3.80)
United Mexican States	-2.60 (-3.20 - -2.00)	-2.10 (-2.60 - -1.60)	-3.20 (-3.90 - -2.40)	-2.10 (-2.50 - -1.70)	-1.70 (-2.10 - -1.30)	-2.70 (-3.20 - -2.20)
United Republic of Tanzania	1.60 (1.50 - 1.80)	1.80 (1.60 - 2.00)	1.50 (1.20 - 1.80)	1.40 (1.30 - 1.60)	1.60 (1.40 - 1.80)	1.30 (1.10 - 1.40)
United States of America	-2.50 (-2.90 - -2.00)	-2.80 (-3.20 - -2.40)	-2.20 (-2.60 - -1.80)	-1.90 (-2.20 - -1.60)	-2.20 (-2.50 - -1.80)	-1.70 (-2.00 - -1.40)
United States Virgin Islands	-2.50 (-2.90 - -2.20)	-1.50 (-1.90 - -1.20)	-3.30 (-3.60 - -2.90)	-2.40 (-2.60 - -2.10)	-1.60 (-1.90 - -1.20)	-2.90 (-3.20 - -2.60)

ASMR Age-Standardized Disability-Adjusted Life Year Rate, ASDR Age-Standardized Disability-Adjusted Life

Year Rate, AAPC annual average percent change, CI confidence interval.

Table S7 The Number of Death and DALYs in five SDI levels in 2021

Location	Death/1000（95%UI）			DALYs/1000（95%UI）		
	**Both**	**Male**	**Female**	**Both**	**Male**	**Female**
High SDI	132.05 (37.82,232.6)	57.35 (17.77,99.72)	74.7 (21.46,133.86)	2663.22 (900.96,4379.68)	1338.65 (469.15,2163.64)	1324.57 (431.59,2219.11)
High-middle SDI	318.95 (101.99,547.55)	150.32 (49.01,252.44)	168.63 (52.6,292.85)	6609.15 (2320.77,10837.82)	3437.26 (1241.03,5581.65)	3171.89 (1068.17,5272.95)
Low SDI	40.24 (12.36,71.05)	20.71 (6.38,36.79)	19.52 (5.75,35.28)	1074.12 (379.64,1799.3)	555.7 (188.98,958.04)	518.41 (178.29,883.87)
Low-middle SDI	140.67 (45.3,242.72)	70.17 (23.12,121.58)	70.5 (21.96,122.71)	3460.22 (1236.73,5729.94)	1773.79 (634.27,2971.92)	1686.43 (612.91,2774.59)
Middle SDI	303.16 (98.31,528.05)	162.34 (53.4,278.32)	140.82 (44.8,249.64)	7148.66 (2541.45,11776.12)	3923.71 (1423.89,6423.24)	3224.95 (1122.35,5359.21)

DALYs Disability-Adjusted Life Years, SDI Socio-Demographic Index.

Table S8 The Slope Index of Inequality and Concentration Index for deaths and DALYs from 1990 to 2021.

		Slope Index of Inequality (95% CI) (1990/2021)	Concentration Index (95% CI) (1990/2021)
Deaths	Both	14.82 (11.55,18.08) / 9.61 (736,11.86)	-0.28 (-0.36, -0.21) / -0.13 (-0.23, -0.03)
	Male	11.63 (8.87,14.40) / 7.67 (5.52,9.82)	-0.21 (-0.29, -0.14) / -0.11 (-0.21, -0.01)
	Female	17.52 (13.70,21.34) / 11.47 (9.03,13.92)	-0.33 (-0.41, -0.26) / -0.16 (-0.26, -0.06)
DALYs	Both	254.17 (194.59,313.76) / 155.19 (110.16,200.22)	-0.20 (-0.29, -0.16) / -0.09 (-0.20, 0.01)
	Male	238.17 (18070,295.65) / 145.85 (96.96,194.73)	-0.17 (-0.25, -0.09) / -0.09 (-0.19, 0.01)
	Female	272.95 (209.98,335.92) / 164.45 (120.16,208.75)	-0.24 (-0.32, -0.16) / -0.10 (-0.20, 0.00)

DALYs Disability-Adjusted Life Years, CI confidence interval.

Table S9 Frontier analysis results of ASMR across countries and regions.

Location	Year	Val	SDI	Frontier	Eff diff	Trend
American Samoa	2021	8.660828479	0.723727533	3.20763642	5.45319206	Decrease
Antigua and Barbuda	2021	9.034980768	0.749886887	3.18390987	5.851070898	Decrease
Arab Republic of Egypt	2021	36.86560328	0.606787094	3.543770482	33.3218328	Decrease
Argentine Republic	2021	5.246085819	0.723122973	3.199740583	2.046345237	Decrease
Australia	2021	3.687222461	0.844252814	2.501097298	1.186125163	Decrease
Barbados	2021	11.12919696	0.746748764	3.205160799	7.924036156	Decrease
Belize	2021	6.336959658	0.610229002	3.577076903	2.759882755	Decrease
Bermuda	2021	5.126819702	0.821365422	2.522966036	2.603853666	Decrease
Bolivarian Republic of Venezuela	2021	6.317747328	0.596513059	3.630521695	2.687225632	Decrease
Bosnia and Herzegovina	2021	25.94245073	0.723077893	3.247257571	22.69519316	Decrease
Brunei Darussalam	2021	10.44373651	0.810234367	2.830267706	7.613468799	Decrease
Burkina Faso	2021	6.737517776	0.285118402	4.485428305	2.252089471	Decrease
Canada	2021	3.11729375	0.87317068	2.036657169	1.080636581	Decrease

Central African Republic	2021	14.43514464	0.30916769	4.355566441	10.0795782	Decrease
Commonwealth of Dominica	2021	12.03521189	0.746967185	3.226878723	8.808333168	Decrease
Commonwealth of the Bahamas	2021	7.332081272	0.805020668	2.91516745	4.416913822	Decrease
Cook Islands	2021	6.027052448	0.779109955	3.20003967	2.827012778	Decrease
Czech Republic	2021	9.053535728	0.828450433	2.4893949	6.564140828	Decrease
Democratic People's Republic of Korea	2021	18.98097672	0.569854634	4.240576664	14.74040005	Decrease
Democratic Republic of Sao Tome and Principe	2021	16.71125187	0.505413747	4.318162752	12.39308912	Increase
Democratic Republic of the Congo	2021	11.51014543	0.383179849	4.319252046	7.190893383	Decrease
Democratic Republic of Timor-Leste	2021	15.05624152	0.444667619	4.319647193	10.73659432	Increase
Democratic Socialist Republic of Sri Lanka	2021	19.31132815	0.701534935	3.184256389	16.12707176	Decrease
Dominican Republic	2021	9.215964433	0.619388201	3.522574328	5.693390106	Decrease
Eastern Republic of Uruguay	2021	9.420114312	0.719283445	3.275328904	6.144785408	Decrease
Federal Democratic Republic of Ethiopia	2021	4.45827753	0.358823295	4.31893234	0.13934519	Decrease
Federal Democratic Republic of Nepal	2021	8.850365639	0.433174635	4.319241779	4.53112386	Decrease
Federal Republic of Germany	2021	5.2412077	0.902957091	1.938504896	3.302702804	Decrease
Federal Republic of Nigeria	2021	13.28777022	0.503390833	4.318929569	8.968840649	Decrease
Federal Republic of Somalia	2021	7.676958681	0.077688109	7.676958681	0	Decrease
Federated States of Micronesia	2021	14.25564598	0.587534967	3.945287896	10.31035809	Decrease
Federative Republic of Brazil	2021	8.621449168	0.653043887	3.191456297	5.429992872	Decrease
French Republic	2021	3.879266089	0.838364875	2.500613702	1.378652387	Decrease
Gabonese Republic	2021	12.29330851	0.634691393	3.278031993	9.015276515	Decrease
Georgia	2021	22.20867299	0.84656427	2.481947548	19.72672544	Increase
Georgia	2021	22.20867299	0.732473604	3.185709719	19.02296327	Increase
Grand Duchy of Luxembourg	2021	4.363165209	0.884428955	1.937323824	2.425841385	Decrease
Greenland	2021	9.058799763	0.826210336	2.483698733	6.57510103	Decrease
Grenada	2021	12.81612892	0.668993028	3.215317423	9.600811495	Decrease
Guam	2021	3.448995489	0.803982203	2.916550751	0.532444738	Decrease
Hashemite Kingdom of Jordan	2021	16.03416507	0.725307227	3.20862562	12.82553945	Decrease
Hellenic Republic	2021	7.636650174	0.791854408	3.05694667	4.579703504	Decrease
Hungary	2021	13.49415733	0.790754768	3.243104708	10.25105263	Decrease
Independent State of Papua New Guinea	2021	9.902913237	0.417797443	4.318860279	5.584052959	Decrease
Independent State of Samoa	2021	9.437408672	0.593392769	3.635389457	5.802019214	Decrease
Ireland	2021	3.589712832	0.87375385	1.935667933	1.654044899	Decrease
Islamic Republic of Afghanistan	2021	29.15934723	0.337199998	4.318917143	24.84043009	Decrease
Islamic Republic of Iran	2021	14.74577651	0.697207398	3.198376892	11.54739962	Decrease
Islamic Republic of Mauritania	2021	16.28123402	0.4989451	4.319750823	11.9614832	Decrease
Islamic Republic of Pakistan	2021	11.44640597	0.504028689	4.320964457	7.125441513	Increase
Jamaica	2021	11.07280282	0.683263064	3.214145349	7.85865747	Decrease
Japan	2021	3.677357095	0.871241813	1.928760811	1.748596284	Decrease
Kingdom of Bahrain	2021	16.13643594	0.753043204	3.206529717	12.92990622	Decrease
Kingdom of Belgium	2021	4.085359381	0.853654016	2.070147873	2.015211508	Decrease
Kingdom of Bhutan	2021	10.54331221	0.473062378	4.318856732	6.224455478	Decrease
Kingdom of Cambodia	2021	17.57956037	0.473621491	4.31885734	13.26070303	Decrease
Kingdom of Denmark	2021	5.469510892	0.896424204	2.031358956	3.438151935	Decrease

Kingdom of Eswatini	2021	13.67934424	0.585459713	4.100490111	9.578854126	Increase
Kingdom of Lesotho	2021	14.43566644	0.510393066	4.29661868	10.13904776	Increase
Kingdom of Morocco	2021	26.02928633	0.562698301	4.229215697	21.80007063	Decrease
Kingdom of Norway	2021	4.122800372	0.91613281	2.006183637	2.116616735	Decrease
Kingdom of Saudi Arabia	2021	18.17426348	0.815143493	2.622037031	15.55222645	Decrease
Kingdom of Spain	2021	3.655253222	0.769283698	3.289815826	0.365437396	Decrease
Kingdom of Sweden	2021	4.445926087	0.886880299	1.978664461	2.467261626	Decrease
Kingdom of Thailand	2021	8.000990778	0.682547933	3.185236045	4.815754732	Decrease
Kingdom of the Netherlands	2021	5.973478863	0.888464256	1.978143578	3.995335285	Decrease
Kingdom of Tonga	2021	7.987649695	0.626349936	3.468500377	4.519149318	Decrease
Kyrgyz Republic	2021	16.40230177	0.603979328	3.634768616	12.76753315	Decrease
Lao People's Democratic Republic	2021	19.11652154	0.489136091	4.321083527	14.79543801	Decrease
Lebanese Republic	2021	8.273382188	0.744746351	3.190860749	5.082521439	Decrease
Malaysia	2021	13.45749921	0.742523828	3.179152017	10.2783472	Decrease
Mongolia	2021	6.759897065	0.617621565	3.553263646	3.206633419	Increase
Montenegro	2021	17.81903774	0.795800584	3.052084248	14.7669535	Increase
New Zealand	2021	5.143983288	0.849442499	2.503507715	2.640475573	Decrease
North Macedonia	2021	59.36603406	0.750629703	3.218884546	56.14714952	Increase
Northern Mariana Islands	2021	8.586938232	0.771535213	3.191822271	5.395115961	Decrease
Palestine	2021	23.95960218	0.631011665	3.424186294	20.53541589	Decrease
People's Democratic Republic of Algeria	2021	19.3882658	0.659500924	3.297879549	16.09038625	Decrease
People's Republic of Bangladesh	2021	12.78037666	0.492420885	4.318937083	8.461439572	Decrease
People's Republic of China	2021	15.9320645	0.72162976	3.260831934	12.67123257	Decrease
Plurinational State of Bolivia	2021	7.711164006	0.599010799	3.642396475	4.068767531	Decrease
Portuguese Republic	2021	7.886879282	0.744151851	3.189319733	4.697559549	Decrease
Principality of Andorra	2021	3.945489029	0.869444113	1.980050764	1.965438265	Decrease
Principality of Monaco	2021	9.069708141	0.908262831	1.926827307	7.142880834	Decrease
Puerto Rico	2021	2.500364688	0.825525847	2.492179458	0.00818523	Decrease
Republic of Albania	2021	12.08827988	0.706849791	3.192882001	8.895397879	Decrease
Republic of Angola	2021	13.10016522	0.453721949	4.320104751	8.780060471	Decrease
Republic of Armenia	2021	11.2958671	0.701833194	3.188816964	8.107050138	Decrease
Republic of Austria	2021	3.903969429	0.853837004	2.289091932	1.614877496	Decrease
Republic of Azerbaijan	2021	10.00279724	0.694851274	3.197065774	6.805731461	Decrease
Republic of Belarus	2021	20.19859633	0.784484711	3.236366491	16.96222984	Decrease
Republic of Benin	2021	15.69592596	0.373486574	4.321229945	11.37469602	Decrease
Republic of Botswana	2021	10.3993376	0.642721629	3.193898492	7.20543911	Decrease
Republic of Bulgaria	2021	41.87465128	0.768150939	3.219305061	38.65534622	Decrease
Republic of Burundi	2021	9.637686791	0.289374365	4.508145908	5.129540883	Decrease
Republic of Cabo Verde	2021	14.59879363	0.533534539	4.183090672	10.41570296	Increase
Republic of Cameroon	2021	9.954577742	0.479691223	4.318917256	5.635660487	Increase
Republic of Chad	2021	16.15347319	0.240436019	4.742730882	11.41074231	Increase
Republic of Chile	2021	5.772092842	0.771514716	3.194236656	2.577856186	Decrease
Republic of Colombia	2021	3.987859129	0.655442913	3.22903612	0.758823008	Decrease
Republic of Costa Rica	2021	4.331233863	0.700340477	3.188552885	1.142680978	Decrease

Republic of Croatia	2021	13.32034965	0.798341027	3.041508376	10.27884127	Decrease
Republic of Cuba	2021	8.626508445	0.668729864	3.184275911	5.442232534	Decrease
Republic of Cyprus	2021	9.216293374	0.835630545	2.462754021	6.753539353	Decrease
Republic of C 么 te d'Ivoire	2021	16.55322319	NA	1.961293497	14.59192969	Increase
Republic of Djibouti	2021	10.24189476	0.487958371	4.318880996	5.923013763	Decrease
Republic of Ecuador	2021	4.868947202	0.661017053	3.20604125	1.662905952	Decrease
Republic of El Salvador	2021	4.463149279	0.563775188	4.253777296	0.209371982	Decrease
Republic of Equatorial Guinea	2021	12.39632849	0.657857456	3.181819515	9.214508978	Decrease
Republic of Estonia	2021	8.533647273	0.844917787	2.467981855	6.065665418	Decrease
Republic of Fiji	2021	11.54649586	0.675051631	3.227213141	8.319282719	Decrease
Republic of Finland	2021	5.271780384	0.859831368	1.925118021	3.346662362	Decrease
Republic of Ghana	2021	27.05661327	0.56493039	4.250809056	22.80580421	Increase
Republic of Guatemala	2021	4.322040009	0.539972424	4.169688143	0.152351866	Decrease
Republic of Guinea	2021	17.38265504	0.336401293	4.322970469	13.05968457	Increase
Republic of Guinea-Bissau	2021	22.65257083	0.353109621	4.343775104	18.30879573	Increase
Republic of Guyana	2021	17.99548938	0.650812335	3.179397511	14.81609187	Decrease
Republic of Haiti	2021	20.34284208	0.448278285	4.318917138	16.02392495	Decrease
Republic of Honduras	2021	14.16201003	0.513037248	4.315682071	9.846327963	Increase
Republic of Iceland	2021	3.837307523	0.87636168	1.996700378	1.840607145	Decrease
Republic of India	2021	7.341897615	0.575401649	4.09583813	3.246059485	Decrease
Republic of Indonesia	2021	19.42515379	0.656868336	3.19184227	16.23331152	Increase
Republic of Iraq	2021	33.17653335	0.662626231	3.203606855	29.9729265	Decrease
Republic of Italy	2021	5.155102466	0.805773534	2.924709633	2.230392833	Decrease
Republic of Kazakhstan	2021	25.2463822	0.725144495	3.212236528	22.03414567	Decrease
Republic of Kenya	2021	7.241495111	0.523768077	4.281365218	2.960129893	Increase
Republic of Kiribati	2021	13.3446334	0.527186583	4.16035992	9.184273483	Increase
Republic of Korea	2021	6.223444144	0.886675267	1.944639012	4.278805133	Decrease
Republic of Latvia	2021	26.36340365	0.830663516	2.493808296	23.86959535	Decrease
Republic of Liberia	2021	15.91057984	0.352442452	4.325975763	11.58460407	Increase
Republic of Lithuania	2021	17.15344962	0.856484049	1.930564609	15.22288502	Decrease
Republic of Madagascar	2021	13.44162221	0.400246943	4.319747783	9.121874424	Decrease
Republic of Malawi	2021	14.40543889	0.384553634	4.319109117	10.08632977	Increase
Republic of Maldives	2021	10.9728311	0.650886627	3.215894127	7.756936972	Decrease
Republic of Mali	2021	11.21094827	0.268579941	4.558745306	6.652202966	Decrease
Republic of Malta	2021	4.336371189	0.801585034	3.002379696	1.333991493	Decrease
Republic of Mauritius	2021	10.35278762	0.718260446	3.200609988	7.152177634	Decrease
Republic of Moldova	2021	13.43165453	0.732214875	3.269666576	10.16198795	Decrease
Republic of Mozambique	2021	20.51668779	0.326462614	4.329528656	16.18715913	Increase
Republic of Namibia	2021	15.82481383	0.617564872	3.532063746	12.29275008	Decrease
Republic of Nauru	2021	21.77712193	0.625177834	3.517511384	18.25961054	Decrease
Republic of Nicaragua	2021	4.503046317	0.523958472	4.148108027	0.35493829	Decrease
Republic of Niue	2021	10.82737385	0.72622205	3.22608095	7.601292899	Decrease
Republic of Palau	2021	14.60006109	0.754046931	3.183163454	11.41689764	Decrease
Republic of Panama	2021	5.976393964	0.708864828	3.179110992	2.797282972	Decrease

Republic of Paraguay	2021	10.93289255	0.635718099	3.260326015	7.672566531	Decrease
Republic of Peru	2021	3.780227711	0.662054037	3.186988468	0.593239242	Decrease
Republic of Poland	2021	11.97270953	0.812042809	2.723270003	9.249439523	Decrease
Republic of Rwanda	2021	6.979824478	0.435588706	4.318945899	2.660878579	Decrease
Republic of San Marino	2021	4.25216771	0.888005474	1.939982452	2.312185258	Decrease
Republic of Senegal	2021	16.49092865	0.408054193	4.32056784	12.17036081	Decrease
Republic of Serbia	2021	39.71672759	0.792416294	3.02178051	36.69494708	Decrease
Republic of Seychelles	2021	12.70058953	0.730150775	3.183162589	9.517426938	Decrease
Republic of Sierra Leone	2021	17.39679433	0.358665881	4.32248619	13.07430814	Decrease
Republic of Singapore	2021	1.924983283	0.856097766	1.924983283	0	Decrease
Republic of Slovenia	2021	7.028769736	0.842430731	2.467366965	4.561402771	Decrease
Republic of South Africa	2021	13.026719	0.679626598	3.200449462	9.826269534	Increase
Republic of South Sudan	2021	8.084124935	0.278371125	4.526725619	3.557399316	Decrease
Republic of Sudan	2021	19.67970725	0.541949735	4.218636778	15.46107047	Decrease
Republic of Suriname	2021	11.73065205	0.633665739	3.365137307	8.365514746	Decrease
Republic of Tajikistan	2021	17.61493748	0.541511187	4.139062323	13.47587515	Decrease
Republic of the Congo	2021	14.78142291	0.583075236	3.841702106	10.93972081	Decrease
Republic of the Gambia	2021	20.53572804	0.40971416	4.319807714	16.21592032	Increase
Republic of the Marshall Islands	2021	14.32512341	0.574091128	4.177174166	10.14794925	Decrease
Republic of the Niger	2021	11.54248442	0.168072774	5.585168468	5.957315956	Increase
Republic of the Philippines	2021	12.7194633	0.651219329	3.179596706	9.539866592	Decrease
Republic of the Union of Myanmar	2021	17.9353164	0.53390084	4.169407068	13.76590933	Decrease
Republic of Trinidad and Tobago	2021	11.48154381	0.768763254	3.179353664	8.302190145	Decrease
Republic of Tunisia	2021	16.48749518	0.682432216	3.185267572	13.30222761	Decrease
Republic of Turkey	2021	11.89097099	NA	1.996908707	9.894062283	Decrease
Republic of Uganda	2021	6.62524163	0.423261181	4.318921836	2.306319794	Decrease
Republic of Uzbekistan	2021	17.43429516	0.662621694	3.188764301	14.24553086	Decrease
Republic of Vanuatu	2021	14.09488617	0.473100706	4.319134538	9.775751629	Decrease
Republic of Yemen	2021	28.9954119	0.450376375	4.318947437	24.67646446	Decrease
Republic of Zambia	2021	11.00845035	0.505948954	4.318696276	6.689754071	Increase
Republic of Zimbabwe	2021	14.64120237	0.473819486	4.319153918	10.32204845	Increase
Romania	2021	26.18407463	0.768453864	3.181020692	23.00305393	Decrease
Russian Federation	2021	30.95981927	0.808536005	2.870841124	28.08897815	Decrease
Saint Kitts and Nevis	2021	18.2918138	0.754987055	3.244878151	15.04693565	Decrease
Saint Lucia	2021	11.26017137	0.672509735	3.235445051	8.024726315	Decrease
Saint Vincent and the Grenadines	2021	10.71645411	0.637195963	3.228323443	7.488130666	Decrease
Slovak Republic	2021	14.24592846	0.81061053	2.838623475	11.40730499	Decrease
Socialist Republic of Viet Nam	2021	22.94912471	0.627933721	3.415258463	19.53386625	Increase
Solomon Islands	2021	12.95732023	0.429360316	4.343761791	8.613558441	Decrease
State of Eritrea	2021	10.4842517	0.403863943	4.320635779	6.163615924	Decrease
State of Israel	2021	2.721799995	0.809011652	2.721799995	0	Decrease
State of Kuwait	2021	7.488587453	0.846651055	2.479378477	5.009208976	Decrease
State of Libya	2021	15.48392522	0.725771399	3.209632546	12.27429267	Increase
State of Qatar	2021	8.713924989	0.846860584	2.478287883	6.235637106	Decrease

Sultanate of Oman	2021	16.51702746	0.773391602	3.216055208	13.30097225	Decrease
Swiss Confederation	2021	3.342385189	0.933059111	1.93650619	1.405878999	Decrease
Syrian Arab Republic	2021	18.36663467	0.623004075	3.52715558	14.83947909	Decrease
Taiwan (Province of China)	2021	4.27646119	0.874747053	2.02054098	2.255920209	Decrease
Togolese Republic	2021	18.95012544	0.408533695	4.318914547	14.63121089	Increase
Tokelau	2021	10.76711937	0.686425621	3.200441986	7.566677384	Decrease
Turkmenistan	2021	25.13125901	0.682160776	3.228543096	21.90271592	Increase
Tuvalu	2021	13.938565	0.576620529	4.112455447	9.826109556	Decrease
Ukraine	2021	24.50227702	0.760773913	3.180865438	21.32141158	Decrease
Union of the Comoros	2021	11.12673757	0.475978688	4.321406317	6.805331249	Decrease
United Arab Emirates	2021	18.4621902	0.849317734	2.493106178	15.96908402	Decrease
United Kingdom of Great Britain and Northern Ireland	2021	4.162505445	0.859000182	1.929997798	2.232507647	Decrease
United Mexican States	2021	4.887092345	0.664575304	3.206697818	1.680394527	Decrease
United Republic of Tanzania	2021	12.60426204	0.446568273	4.319101568	8.285160471	Increase
United States of America	2021	4.109058113	0.862448354	1.927337163	2.18172095	Decrease
United States Virgin Islands	2021	4.97640006	0.821830853	2.531884862	2.444515198	Decrease

ASMR Age-Standardized Disability-Adjusted Life Year Rate, SDI Socio-Demographic Index.

Table S10 Frontier analysis results of ASDR across countries and regions.

Location	Year	Val	SDI	Frontier	Eff diff	Trend
American Samoa	2021	203.3386804	0.723727533	70.82238266	132.5162978	Decrease
Antigua and Barbuda	2021	158.5112114	0.749886887	70.49104145	88.02017	Decrease
Arab Republic of Egypt	2021	740.7654356	0.606787094	76.24168031	664.5237553	Decrease
Argentine Republic	2021	116.5863007	0.723122973	70.48048614	46.10581458	Decrease
Australia	2021	77.86351929	0.844252814	56.72806	21.13545929	Decrease
Barbados	2021	192.4900747	0.746748764	70.30382514	122.1862496	Decrease
Belize	2021	120.8145181	0.610229002	75.98730308	44.82721503	Decrease
Bermuda	2021	97.5098669	0.821365422	57.67555345	39.83431345	Decrease
Bolivarian Republic of Venezuela	2021	117.3883561	0.596513059	75.57520558	41.81315053	Decrease
Bosnia and Herzegovina	2021	512.8183364	0.723077893	70.27040715	442.5479292	Decrease
Brunei Darussalam	2021	212.5832062	0.810234367	63.81299849	148.7702077	Decrease
Burkina Faso	2021	145.2966173	0.285118402	99.89687172	45.39974553	Decrease
Canada	2021	83.84420659	0.87317068	56.81456753	27.02963905	Decrease
Central African Republic	2021	295.639692	0.30916769	97.19689412	198.4427979	Decrease
Commonwealth of Dominica	2021	209.3769144	0.746967185	71.37618467	138.0007297	Decrease
Commonwealth of the Bahamas	2021	143.3985069	0.805020668	63.55600386	79.84250299	Decrease
Cook Islands	2021	160.509236	0.779109955	70.36581624	90.14341979	Decrease
Czech Republic	2021	191.7356787	0.828450433	56.90189684	134.8337818	Decrease
Democratic People's Republic of Korea	2021	459.9570157	0.569854634	80.77882902	379.1781867	Decrease
Democratic Republic of Sao Tome and Principe	2021	369.060706	0.505413747	90.13526525	278.9254408	Increase
Democratic Republic of the Congo	2021	232.9064101	0.383179849	96.3988064	136.5076037	Decrease
Democratic Republic of Timor-Leste	2021	305.9101949	0.444667619	96.46834077	209.4418541	Increase
Democratic Socialist Republic of Sri Lanka	2021	343.1139925	0.701534935	70.30869558	272.8052969	Decrease

Dominican Republic	2021	195.6246427	0.619388201	75.36507912	120.2595636	Decrease
Eastern Republic of Uruguay	2021	183.7157268	0.719283445	70.34761134	113.3681154	Decrease
Federal Democratic Republic of Ethiopia	2021	98.61095815	0.358823295	96.42178582	2.189172323	Decrease
Federal Democratic Republic of Nepal	2021	175.5598556	0.433174635	96.39584828	79.16400733	Decrease
Federal Republic of Germany	2021	126.6639811	0.902957091	56.91819648	69.74578459	Decrease
Federal Republic of Nigeria	2021	276.2124004	0.503390833	89.65768104	186.5547193	Decrease
Federal Republic of Somalia	2021	172.4144745	0.077688109	172.4144745	0	Decrease
Federated States of Micronesia	2021	331.4549016	0.587534967	77.76822361	253.686678	Decrease
Federative Republic of Brazil	2021	171.3178583	0.653043887	70.40720167	100.9106566	Decrease
French Republic	2021	82.72250857	0.838364875	57.3423589	25.38014967	Decrease
Gabonese Republic	2021	256.2164323	0.634691393	71.55363959	184.6627927	Decrease
Georgia	2021	442.4170292	0.84656427	56.82886927	385.58816	Increase
Georgia	2021	442.4170292	0.732473604	70.35224607	372.0647832	Increase
Grand Duchy of Luxembourg	2021	78.25128766	0.884428955	56.93615818	21.31512948	Decrease
Greenland	2021	177.9875572	0.826210336	56.72823861	121.2593186	Decrease
Grenada	2021	229.6834134	0.668993028	70.3294823	159.3539311	Decrease
Guam	2021	141.3366823	0.803982203	64.01118013	77.32550217	Decrease
Hashemite Kingdom of Jordan	2021	339.3309166	0.725307227	70.41898279	268.9119338	Decrease
Hellenic Republic	2021	128.3553164	0.791854408	69.84498447	58.51033194	Decrease
Hungary	2021	295.1402943	0.790754768	70.00911047	225.1311838	Decrease
Independent State of Papua New Guinea	2021	206.505082	0.417797443	96.40973912	110.0953429	Decrease
Independent State of Samoa	2021	224.8246046	0.593392769	76.59599361	148.228611	Decrease
Ireland	2021	64.00110386	0.87375385	56.72604993	7.27505393	Decrease
Islamic Republic of Afghanistan	2021	637.1483716	0.337199998	96.45517358	540.693198	Decrease
Islamic Republic of Iran	2021	309.0449418	0.697207398	70.26898038	238.7759614	Decrease
Islamic Republic of Mauritania	2021	336.3924505	0.4989451	89.43565489	246.9567956	Decrease
Islamic Republic of Pakistan	2021	246.029669	0.504028689	89.84471712	156.1849519	Increase
Jamaica	2021	196.1009472	0.683263064	70.29858748	125.8023597	Decrease
Japan	2021	97.00033152	0.871241813	56.68151264	40.31881888	Decrease
Kingdom of Bahrain	2021	277.5929236	0.753043204	70.61791161	206.975012	Decrease
Kingdom of Belgium	2021	84.83791845	0.853654016	56.96336288	27.87455557	Decrease
Kingdom of Bhutan	2021	209.809394	0.473062378	90.4253889	119.3840051	Decrease
Kingdom of Cambodia	2021	329.8260239	0.473621491	89.68795349	240.1380704	Decrease
Kingdom of Denmark	2021	102.5991194	0.896424204	56.8434314	45.75568798	Decrease
Kingdom of Eswatini	2021	275.9693853	0.585459713	80.7999663	195.169419	Increase
Kingdom of Lesotho	2021	285.1031874	0.510393066	89.944318	195.1588694	Increase
Kingdom of Morocco	2021	536.9432332	0.562698301	80.98105096	455.9621822	Decrease
Kingdom of Norway	2021	91.88918504	0.91613281	56.79647273	35.0927123	Decrease
Kingdom of Saudi Arabia	2021	391.5852378	0.815143493	58.87559557	332.7096423	Decrease
Kingdom of Spain	2021	79.62532142	0.769283698	70.51878281	9.106538608	Decrease
Kingdom of Sweden	2021	93.27256574	0.886880299	57.07648269	36.19608306	Decrease
Kingdom of Thailand	2021	204.9600179	0.682547933	70.30853295	134.6514849	Decrease
Kingdom of the Netherlands	2021	109.692796	0.888464256	56.77787394	52.91492207	Decrease
Kingdom of Tonga	2021	185.7995535	0.626349936	74.188673	111.6108805	Decrease

Kyrgyz Republic	2021	411.5809575	0.603979328	76.03346709	335.5474904	Decrease
Lao People's Democratic Republic	2021	398.8631517	0.489136091	92.05269764	306.8104541	Decrease
Lebanese Republic	2021	186.1519954	0.744746351	70.42626997	115.7257254	Decrease
Malaysia	2021	313.3948551	0.742523828	70.33661454	243.0582406	Decrease
Mongolia	2021	188.5687383	0.617621565	75.90814653	112.6605918	Increase
Montenegro	2021	290.8785509	0.795800584	64.75602278	226.1225281	Increase
New Zealand	2021	94.44934183	0.849442499	57.02518154	37.42416029	Decrease
North Macedonia	2021	905.7424951	0.750629703	70.31124069	835.4312544	Decrease
Northern Mariana Islands	2021	196.087588	0.771535213	70.83009841	125.2574896	Decrease
Palestine	2021	431.5451197	0.631011665	74.6967788	356.8483409	Decrease
People's Democratic Republic of Algeria	2021	348.5270567	0.659500924	70.3249431	278.2021136	Decrease
People's Republic of Bangladesh	2021	233.0252515	0.492420885	89.54807848	143.477173	Decrease
People's Republic of China	2021	335.5864366	0.72162976	71.20548732	264.3809493	Decrease
Plurinational State of Bolivia	2021	144.4988086	0.599010799	75.88251187	68.61629674	Decrease
Portuguese Republic	2021	125.6852954	0.744151851	70.59439451	55.0909009	Decrease
Principality of Andorra	2021	77.89292302	0.869444113	56.98009832	20.9128247	Decrease
Principality of Monaco	2021	159.5885755	0.908262831	56.83533514	102.7532403	Decrease
Puerto Rico	2021	56.98096848	0.825525847	56.97240962	0.008558858	Decrease
Republic of Albania	2021	210.0215519	0.706849791	70.28756286	139.733989	Decrease
Republic of Angola	2021	273.9993968	0.453721949	92.65997163	181.3394252	Decrease
Republic of Armenia	2021	251.0040796	0.701833194	70.28896434	180.7151152	Decrease
Republic of Austria	2021	99.39757421	0.853837004	56.95096856	42.44660565	Decrease
Republic of Azerbaijan	2021	228.7142913	0.694851274	70.2894727	158.4248186	Decrease
Republic of Belarus	2021	432.5430361	0.784484711	70.69797618	361.8450599	Decrease
Republic of Benin	2021	317.5607556	0.373486574	96.41625112	221.1445045	Decrease
Republic of Botswana	2021	222.0963796	0.642721629	70.36267229	151.7337073	Decrease
Republic of Bulgaria	2021	775.2213887	0.768150939	73.5749209	701.6464678	Decrease
Republic of Burundi	2021	203.339274	0.289374365	99.92578711	103.4134869	Decrease
Republic of Cabo Verde	2021	306.0986881	0.533534539	86.83465167	219.2640364	Increase
Republic of Cameroon	2021	215.4183173	0.479691223	89.61700297	125.8013144	Increase
Republic of Chad	2021	346.3360312	0.240436019	105.2519344	241.0840968	Increase
Republic of Chile	2021	118.4806627	0.771514716	70.32567264	48.15499003	Decrease
Republic of Colombia	2021	85.44791732	0.655442913	70.91971243	14.52820489	Decrease
Republic of Costa Rica	2021	84.69441265	0.700340477	71.0769541	13.61745854	Decrease
Republic of Croatia	2021	240.0405297	0.798341027	68.45486268	171.585667	Decrease
Republic of Cuba	2021	168.0652158	0.668729864	70.31175879	97.75345705	Decrease
Republic of Cyprus	2021	123.0399186	0.835630545	57.15794641	65.88197223	Decrease
Republic of C 么 te d'Ivoire	2021	361.0650143	NA	56.91443313	304.1505812	Decrease
Republic of Djibouti	2021	225.3866925	0.487958371	91.74518528	133.6415073	Decrease
Republic of Ecuador	2021	96.14013248	0.661017053	70.37659135	25.76354114	Decrease
Republic of El Salvador	2021	94.24492868	0.563775188	80.87750245	13.36742623	Decrease
Republic of Equatorial Guinea	2021	256.121459	0.657857456	70.65305931	185.4683997	Decrease
Republic of Estonia	2021	192.9897601	0.844917787	56.80841691	136.1813432	Decrease
Republic of Fiji	2021	264.5891234	0.675051631	70.63306818	193.9560552	Decrease

Republic of Finland	2021	114.166248	0.859831368	56.78791504	57.37833299	Decrease
Republic of Ghana	2021	591.4439688	0.56493039	80.76737094	510.6765979	Increase
Republic of Guatemala	2021	80.7598444	0.539972424	80.7598444	0	Decrease
Republic of Guinea	2021	373.5230176	0.336401293	96.44586555	277.0771521	Increase
Republic of Guinea-Bissau	2021	491.1283428	0.353109621	96.40414196	394.7242009	Decrease
Republic of Guyana	2021	346.8650526	0.650812335	70.31274248	276.5523101	Decrease
Republic of Haiti	2021	383.0592041	0.448278285	93.67238585	289.3868183	Decrease
Republic of Honduras	2021	255.1550341	0.513037248	89.52631454	165.6287196	Increase
Republic of Iceland	2021	79.55474824	0.87636168	56.66185087	22.89289737	Decrease
Republic of India	2021	157.5451319	0.575401649	80.79520906	76.74992288	Decrease
Republic of Indonesia	2021	404.9020268	0.656868336	70.27918655	334.6228402	Increase
Republic of Iraq	2021	668.4096299	0.662626231	70.26688723	598.1427427	Decrease
Republic of Italy	2021	85.99447027	0.805773534	64.68222798	21.31224229	Decrease
Republic of Kazakhstan	2021	509.1092764	0.725144495	70.38910312	438.7201733	Decrease
Republic of Kenya	2021	152.7930704	0.523768077	86.77774614	66.01532423	Increase
Republic of Kiribati	2021	327.8823078	0.527186583	89.01139611	238.8709117	Increase
Republic of Korea	2021	135.1222232	0.886675267	56.87562444	78.24659873	Decrease
Republic of Latvia	2021	492.3677853	0.830663516	57.00912695	435.3586583	Decrease
Republic of Liberia	2021	333.6757456	0.352442452	96.42229472	237.2534509	Increase
Republic of Lithuania	2021	353.8237113	0.856484049	56.95473665	296.8689746	Decrease
Republic of Madagascar	2021	294.0621679	0.400246943	96.40242156	197.6597464	Decrease
Republic of Malawi	2021	297.7785056	0.384553634	96.4059657	201.3725399	Increase
Republic of Maldives	2021	219.8674528	0.650886627	70.28571773	149.5817351	Decrease
Republic of Mali	2021	236.6553441	0.268579941	100.3075496	136.3477944	Decrease
Republic of Malta	2021	79.40668988	0.801585034	63.67633001	15.73035987	Decrease
Republic of Mauritius	2021	244.7682733	0.718260446	70.77333678	173.9949365	Decrease
Republic of Moldova	2021	311.9179109	0.732214875	70.39999529	241.5179156	Decrease
Republic of Mozambique	2021	450.1968017	0.326462614	96.40490751	353.7918942	Increase
Republic of Namibia	2021	303.6499022	0.617564872	75.48704717	228.162855	Decrease
Republic of Nauru	2021	525.736771	0.625177834	75.42160981	450.3151612	Decrease
Republic of Nicaragua	2021	93.70266393	0.523958472	86.74946551	6.953198417	Decrease
Republic of Niue	2021	252.0329522	0.72622205	70.28779913	181.7451531	Decrease
Republic of Palau	2021	340.7827666	0.754046931	70.55676587	270.2260007	Decrease
Republic of Panama	2021	110.696761	0.708864828	70.71982402	39.97693697	Decrease
Republic of Paraguay	2021	197.9763014	0.635718099	71.36036205	126.6159393	Decrease
Republic of Peru	2021	82.12814469	0.662054037	70.37970382	11.74844087	Decrease
Republic of Poland	2021	236.4737778	0.812042809	65.62278612	170.8509916	Decrease
Republic of Rwanda	2021	143.6287291	0.435588706	96.43304639	47.1956827	Decrease
Republic of San Marino	2021	88.90587238	0.888005474	56.80452703	32.10134535	Decrease
Republic of Senegal	2021	343.5765035	0.408054193	96.42807551	247.148428	Decrease
Republic of Serbia	2021	675.9751958	0.792416294	66.45701577	609.5181801	Decrease
Republic of Seychelles	2021	283.2416951	0.730150775	70.51813987	212.7235552	Decrease
Republic of Sierra Leone	2021	380.731256	0.358665881	96.39560055	284.3356554	Decrease
Republic of Singapore	2021	70.02509821	0.856097766	56.70728835	13.31780986	Decrease

Republic of Slovenia	2021	131.5933141	0.842430731	56.83814883	74.75516531	Decrease
Republic of South Africa	2021	263.0052987	0.679626598	71.00482178	192.000477	Increase
Republic of South Sudan	2021	177.008703	0.278371125	100.3088844	76.69981858	Decrease
Republic of Sudan	2021	429.7110424	0.541949735	80.76889942	348.942143	Decrease
Republic of Suriname	2021	233.9434626	0.633665739	72.54219797	161.4012647	Decrease
Republic of Tajikistan	2021	373.4621657	0.541511187	80.91874497	292.5434207	Decrease
Republic of the Congo	2021	308.6561569	0.583075236	80.83870943	227.8174474	Decrease
Republic of the Gambia	2021	432.5961982	0.40971416	96.44204772	336.1541505	Increase
Republic of the Marshall Islands	2021	324.2732157	0.574091128	80.99614276	243.2770729	Decrease
Republic of the Niger	2021	234.2585019	0.168072774	127.5436078	106.7148941	Decrease
Republic of the Philippines	2021	292.5719878	0.651219329	71.0007986	221.5711892	Increase
Republic of the Union of Myanmar	2021	371.5564347	0.53390084	87.66630699	283.8901277	Decrease
Republic of Trinidad and Tobago	2021	219.8324911	0.768763254	70.56642769	149.2660634	Decrease
Republic of Tunisia	2021	323.2213166	0.682432216	71.29256832	251.9287483	Decrease
Republic of Turkey	2021	225.496759	NA	58.1324428	167.3643162	Decrease
Republic of Uganda	2021	147.991569	0.423261181	96.44741836	51.54415065	Decrease
Republic of Uzbekistan	2021	419.9895846	0.662621694	70.8204204	349.1691642	Decrease
Republic of Vanuatu	2021	335.7032536	0.473100706	90.14733993	245.5559137	Decrease
Republic of Yemen	2021	601.3043016	0.450376375	93.6434621	507.6608395	Decrease
Republic of Zambia	2021	227.5691737	0.505948954	90.18631371	137.38286	Increase
Republic of Zimbabwe	2021	305.8387344	0.473819486	89.82220306	216.0165314	Increase
Romania	2021	475.1058035	0.768453864	70.37598968	404.7298138	Decrease
Russian Federation	2021	609.697644	0.808536005	63.52766122	546.1699828	Decrease
Saint Kitts and Nevis	2021	317.9743178	0.754987055	71.16260438	246.8117135	Decrease
Saint Lucia	2021	189.0736423	0.672509735	70.59174952	118.4818928	Decrease
Saint Vincent and the Grenadines	2021	188.6785028	0.637195963	70.5490392	118.1294636	Decrease
Slovak Republic	2021	307.7587805	0.81061053	63.4573552	244.3014253	Decrease
Socialist Republic of Viet Nam	2021	446.8339234	0.627933721	74.76368022	372.0702431	Increase
Solomon Islands	2021	296.1819402	0.429360316	96.48727903	199.6946612	Decrease
State of Eritrea	2021	222.8170591	0.403863943	96.47486124	126.3421978	Decrease
State of Israel	2021	66.26676064	0.809011652	64.23748206	2.02927858	Decrease
State of Kuwait	2021	178.535576	0.846651055	57.19875314	121.3368228	Decrease
State of Libya	2021	354.8545152	0.725771399	70.67748579	284.1770294	Increase
State of Qatar	2021	167.6142175	0.846860584	56.81295529	110.8012622	Decrease
Sultanate of Oman	2021	346.2872677	0.773391602	70.3139399	275.9733278	Decrease
Swiss Confederation	2021	66.98807644	0.933059111	57.08503457	9.903041868	Decrease
Syrian Arab Republic	2021	373.7814421	0.623004075	76.12101236	297.6604297	Decrease
Taiwan (Province of China)	2021	134.3210921	0.874747053	56.82102586	77.50006621	Decrease
Togolese Republic	2021	406.7193252	0.408533695	96.41944539	310.2998798	Increase
Tokelau	2021	246.3447705	0.686425621	70.36378104	175.9809895	Decrease
Turkmenistan	2021	625.2650454	0.682160776	70.41137616	554.8536692	Increase
Tuvalu	2021	314.1046222	0.576620529	80.77135526	233.3332669	Decrease
Ukraine	2021	516.2445009	0.760773913	70.43420216	445.8102987	Decrease
Union of the Comoros	2021	238.5946848	0.475978688	91.48888148	147.1058034	Decrease

United Arab Emirates	2021	363.675055	0.849317734	56.8845402	306.7905148	Decrease
United Kingdom of Great Britain and Northern Ireland	2021	84.50295267	0.859000182	56.83890893	27.66404374	Decrease
United Mexican States	2021	103.7357697	0.664575304	71.48129749	32.25447225	Decrease
United Republic of Tanzania	2021	263.8555733	0.446568273	96.41726721	167.4383061	Increase
United States of America	2021	105.0752375	0.862448354	56.83344276	48.24179478	Decrease
United States Virgin Islands	2021	95.44036467	0.821830853	59.06348519	36.37687948	Decrease

ASDR Age-Standardized Disability-Adjusted Life Year Rate, SDI Socio-Demographic Index.

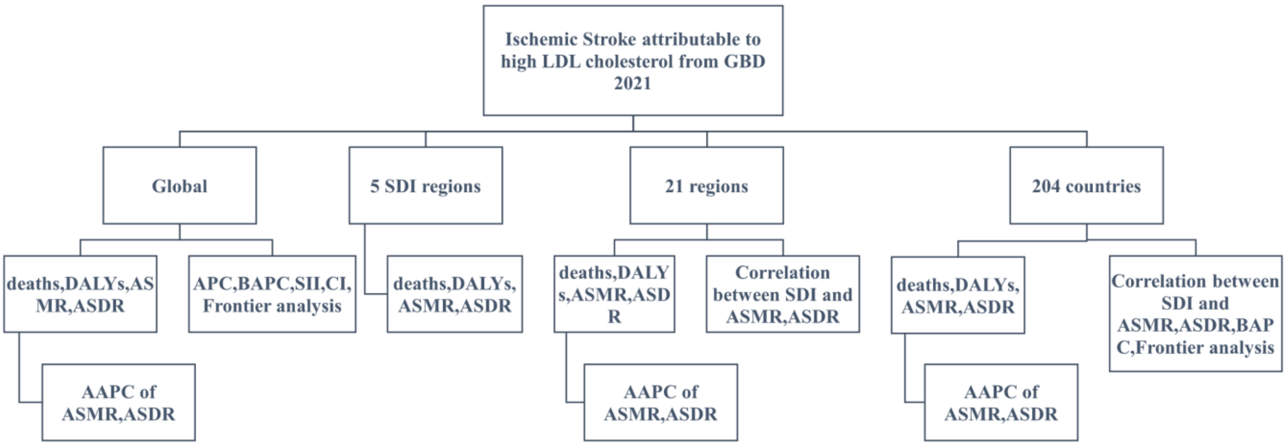

Fig. S1 Flowcharting of the analysis process. GBD Global Burden of Disease Study, D DALYs Disability-Adjusted Life Years, ASMR Age-Standardized Mortality Rate, ASDR Age-Standardized Disability-Adjusted Life Year Rate, SDI Socio-Demographic Index, APC Age-period-cohort, AAPC annual average percent change, BAPC Bayesian Age-Period-Cohort, SII the Slope Index of Inequality, CI the Concentration Index.

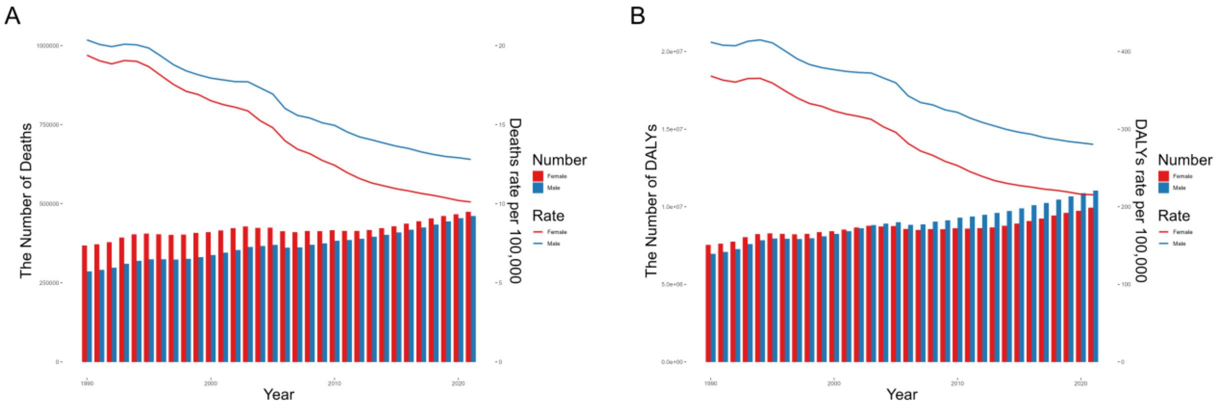

Fig. S2 The global number and age-standardized rates due to Ischemic Stroke attributable to high LDL cholesterol during 1990 − 2021 by sex. **(A)** The global number and ASMR of Ischemic Stroke attributable to high LDL cholesterol during 1990 − 2021 by sex. **(B)** The global number and ASDR of Ischemic Stroke attributable to high LDL cholesterol during 1990 − 2021 by sex. DALYs Disability-Adjusted Life Years, ASMR Age-Standardized

Mortality Rate, ASDR Age-Standardized Disability-Adjusted Life Year Rate.

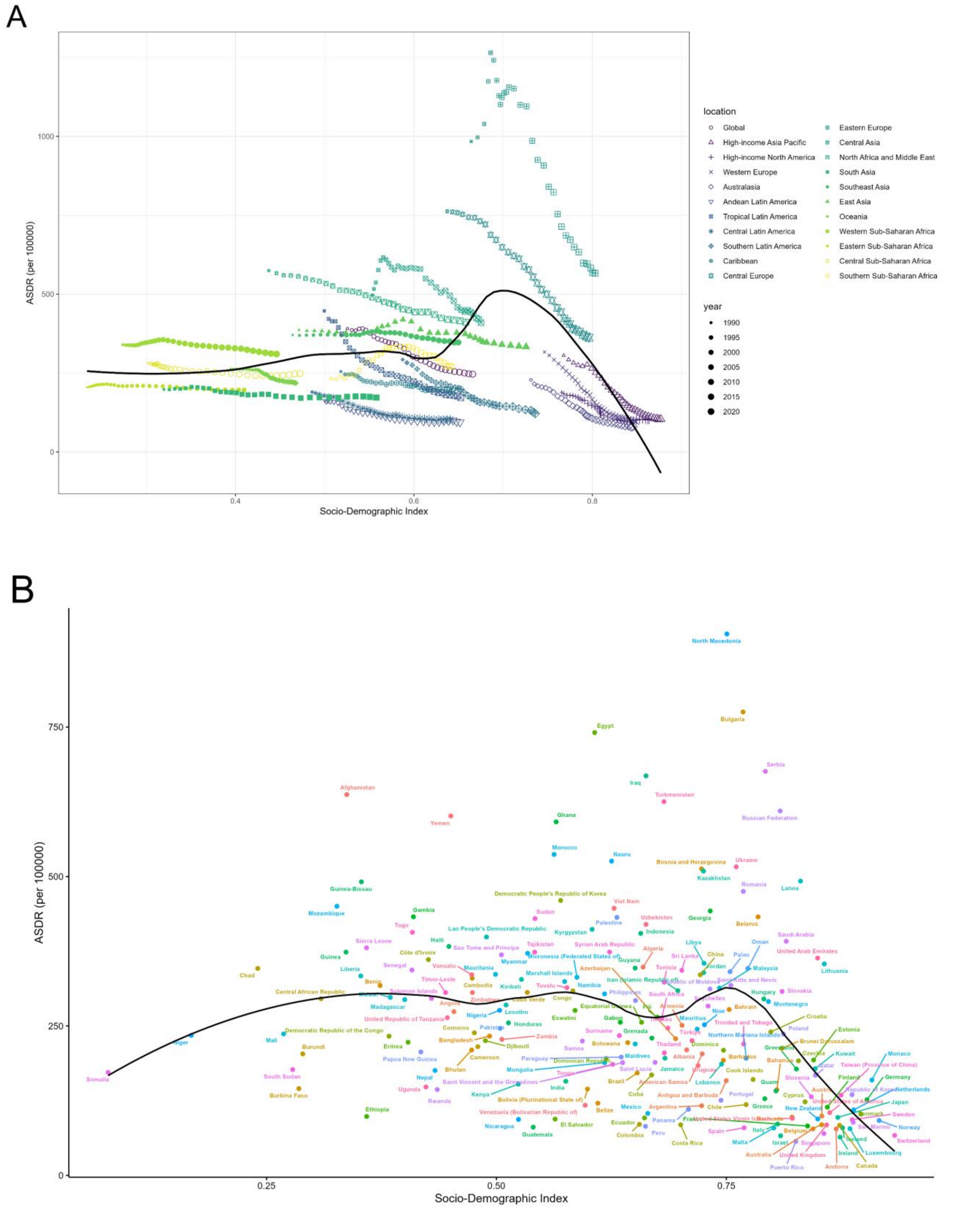

Fig. S3 ASDRs for ischemic stroke attributable to high LDL cholesterol of 21 regions and 204 countries and territories by SDI. **A.** ASDRs for ischemic stroke attributable to high LDL cholesterol of 21 regions from 1990 − 2021 according

to the SDI. **B.** ASDRs for ischemic stroke attributable to high LDL cholesterol of 204 countries and territories in 2021 according to the SDI. ASDR Age-Standardized Disability-Adjusted Life Year Rate, SDI Socio-Demographic Index.

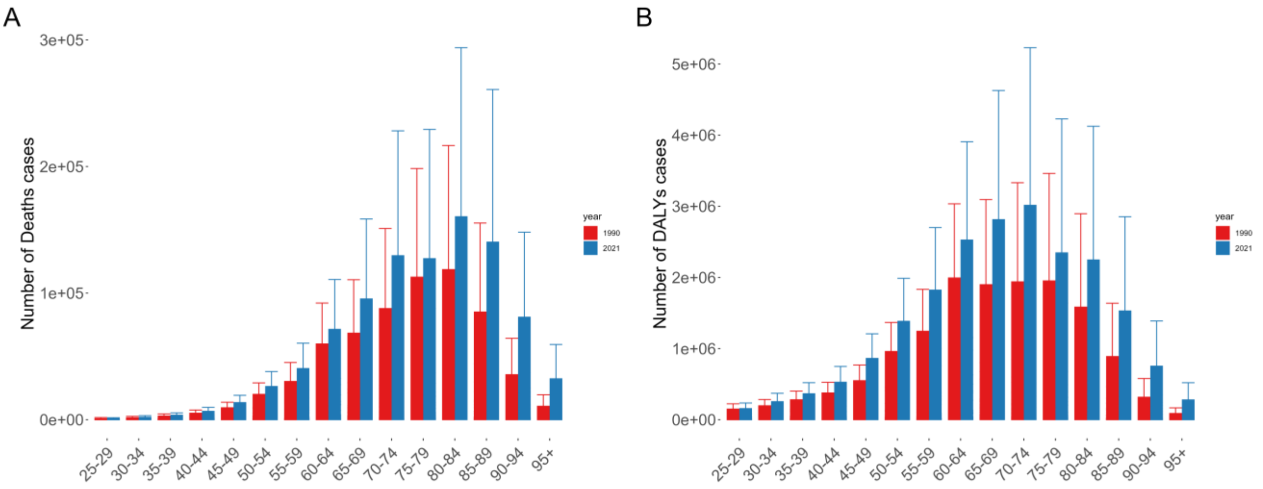

Fig. S4 The global number of deaths and DALYs due to Ischemic Stroke attributable to high LDL cholesterol by age in 2019 and 2021. **(A)** The number of deaths of Ischemic Stroke attributable to high LDL cholesterol by age in 2019 and 2021. **(B)** The number of DALYs of Ischemic Stroke attributable to high LDL cholesterol by age in 2019 and 2021. DALYs Disability-Adjusted Life Years.

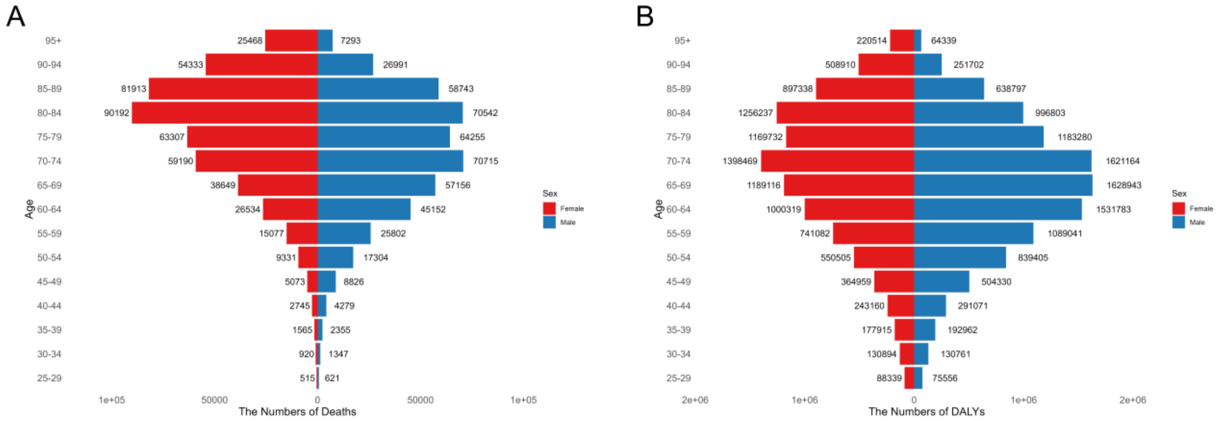

Fig. S5 The global number of deaths and DALYs due to Ischemic Stroke attributable to high LDL cholesterol by age and sex. **(A)** The number of deaths of Ischemic Stroke attributable to high LDL cholesterol in 2021 by sex and age. **(B)** The number of DALYs of Ischemic Stroke attributable to high LDL cholesterol in 2021 by sex and age. DALYs Disability-Adjusted Life Years.

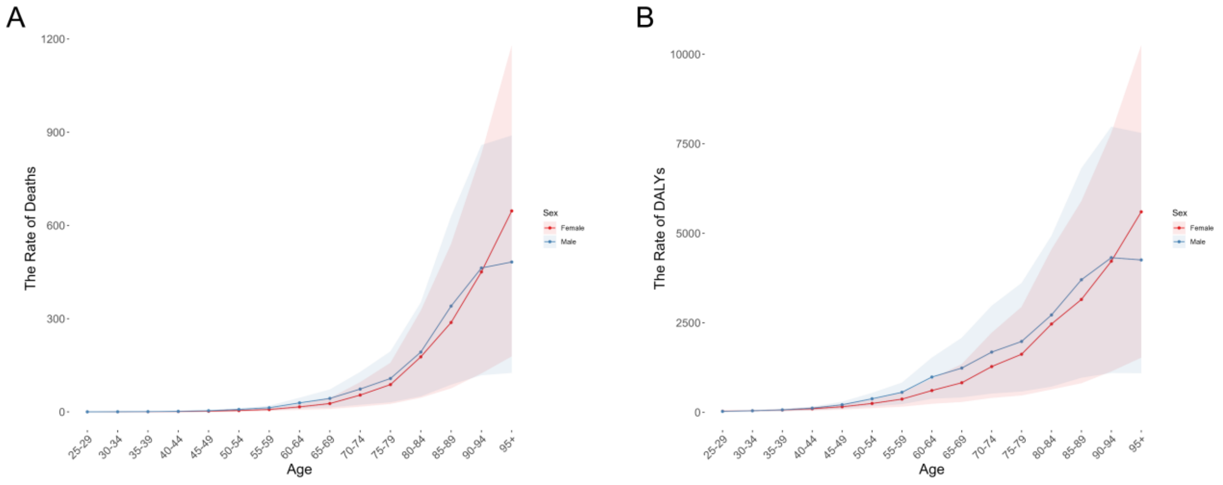

Fig. S6 The global age-standardized rate of deaths and DALYs due to Ischemic Stroke attributable to high LDL cholesterol by age and sex. **(A)** The age-standardized rate of deaths of I Ischemic Stroke attributable to high LDL cholesterol in 2021 by sex and age. **(B)** The age-standardized rate of DALYs of Ischemic Stroke attributable to high LDL cholesterol in 2021 by sex and age. DALYs Disability-Adjusted Life Years.

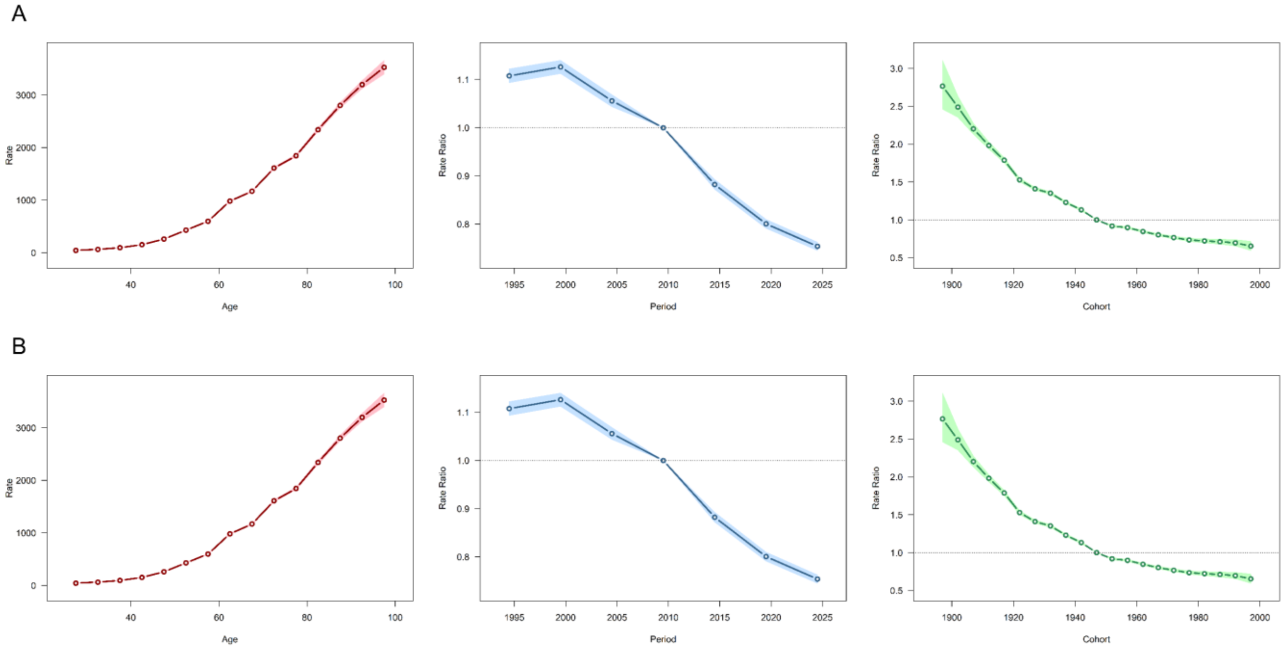

Fig. S7 Analysis of Age, Period, and Birth cohort effects on Ischemic Stroke attributable to high LDL cholesterol globally, focusing on the ASMR and ASDR. **(A)** Analysis of the ASMR on a Global scale**. (B)** Analysis of the ASDR on a Global scale. ASMR Age-Standardized Mortality Rate, ASDR Age-Standardized Disability-Adjusted Life Year Rate.

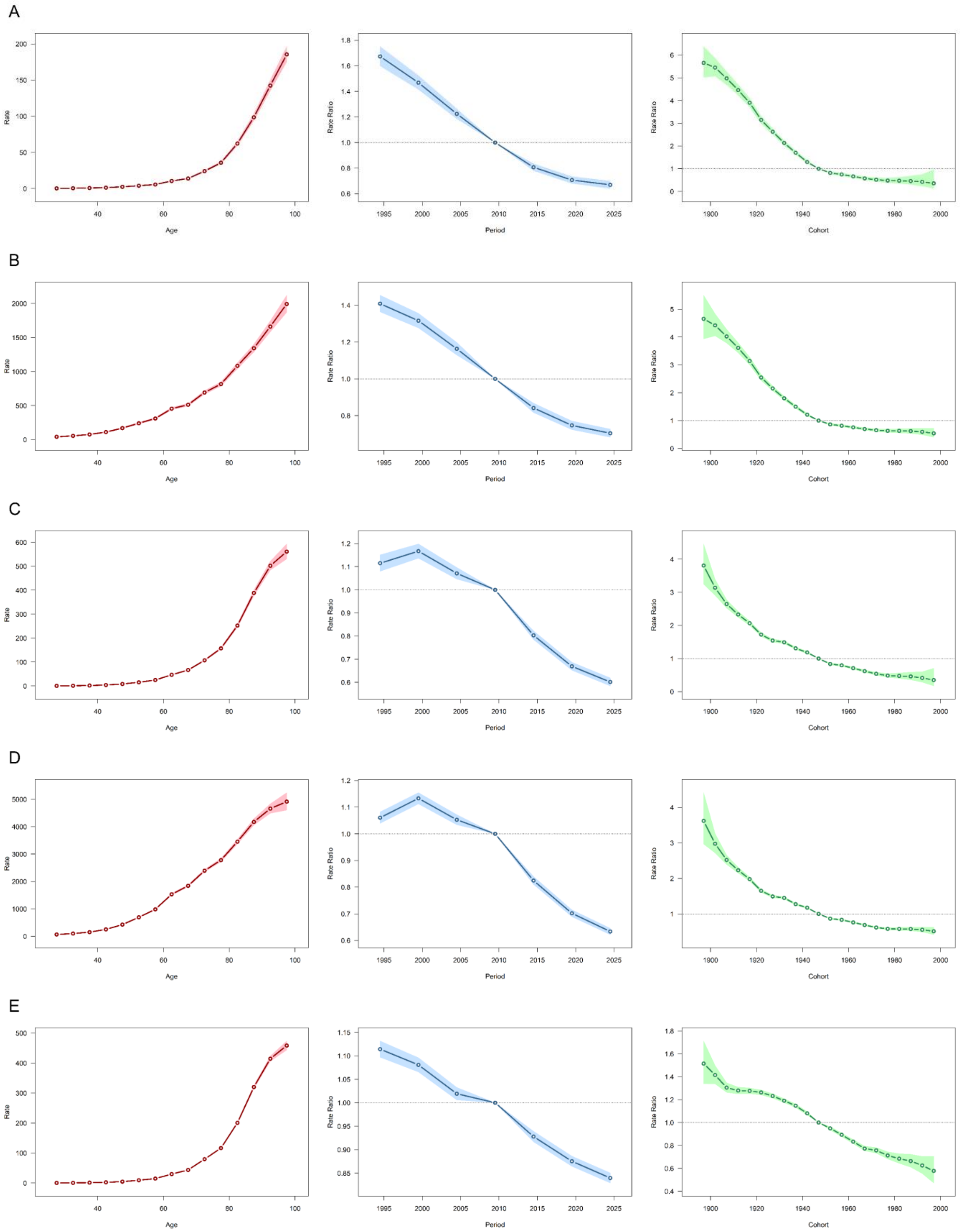
A
B
C
D
E
Rate
Age
Rate Ratio
Period
Rate Ratio
Cohort

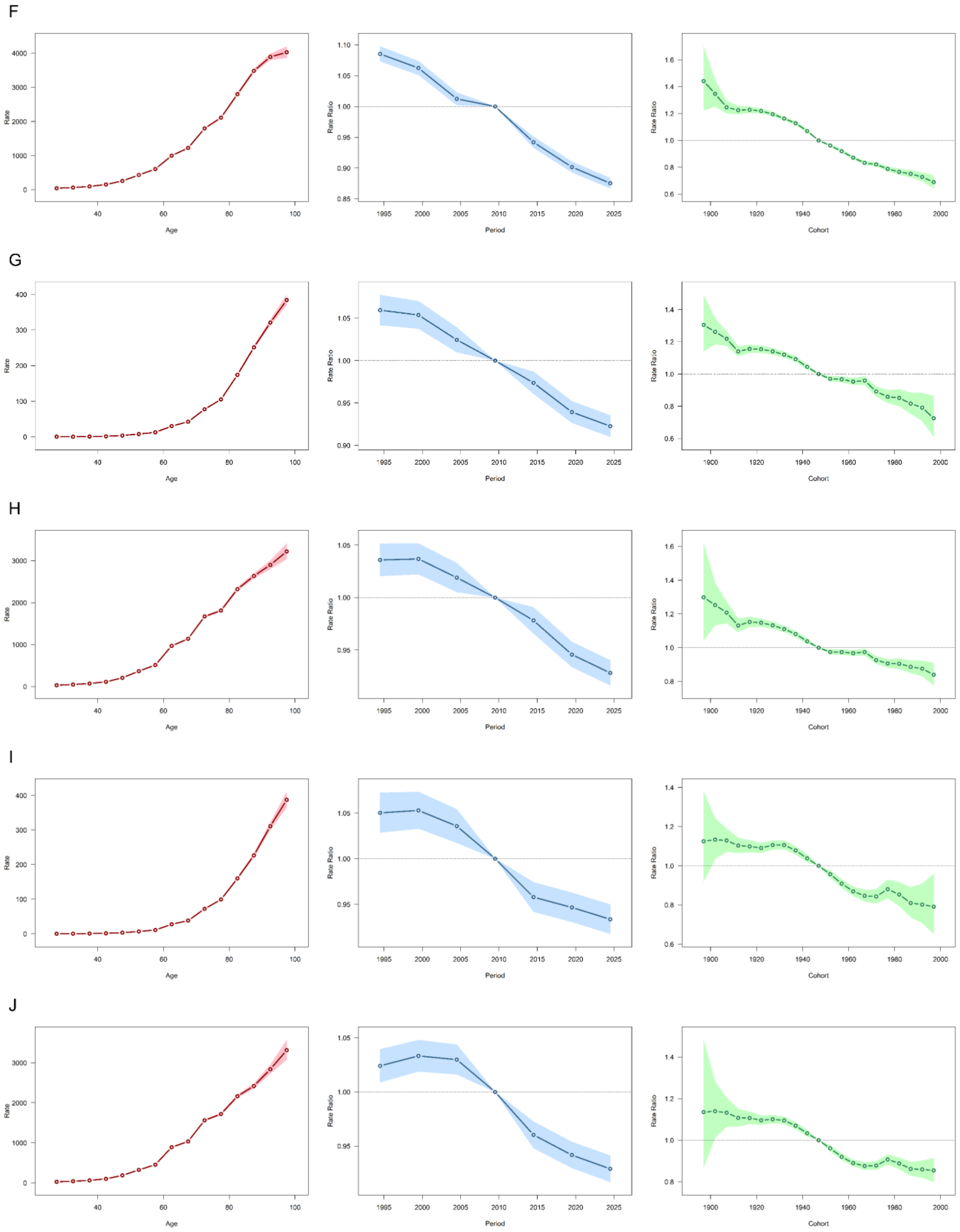

Fig. S8 Analysis of Age, Period, and Birth cohort effects on Ischemic Stroke attributable to high LDL cholesterol across five levels of SDI. **(A)** Analysis of the ASMR in regions with High SDI levels. **(B)** Analysis of the ASDR in regions with High SDI levels, focusing on the ASMR and ASDR. **(C)** Analysis of the ASMR in regions with High-middle SDI levels. **(D)** Analysis of the ASDR in regions with High-middle SDI levels. **(E)** Analysis of the ASMR

in regions with Middle SDI levels. **(F)** Analysis of the ASDR in regions with Middle SDI levels. **(G)** Analysis of the ASMR in regions with Low-middle SDI levels. **(H)** Analysis of the ASDR in regions with Low-middle SDI levels. **(I)** Analysis of the ASMR in regions with Low SDI levels. **(J)** Analysis of the ASDR in regions with Low SDI levels. SDI Socio-Demographic Index, ASMR Age-Standardized Mortality Rate, ASDR Age-Standardized Disability-Adjusted Life Year Rate.

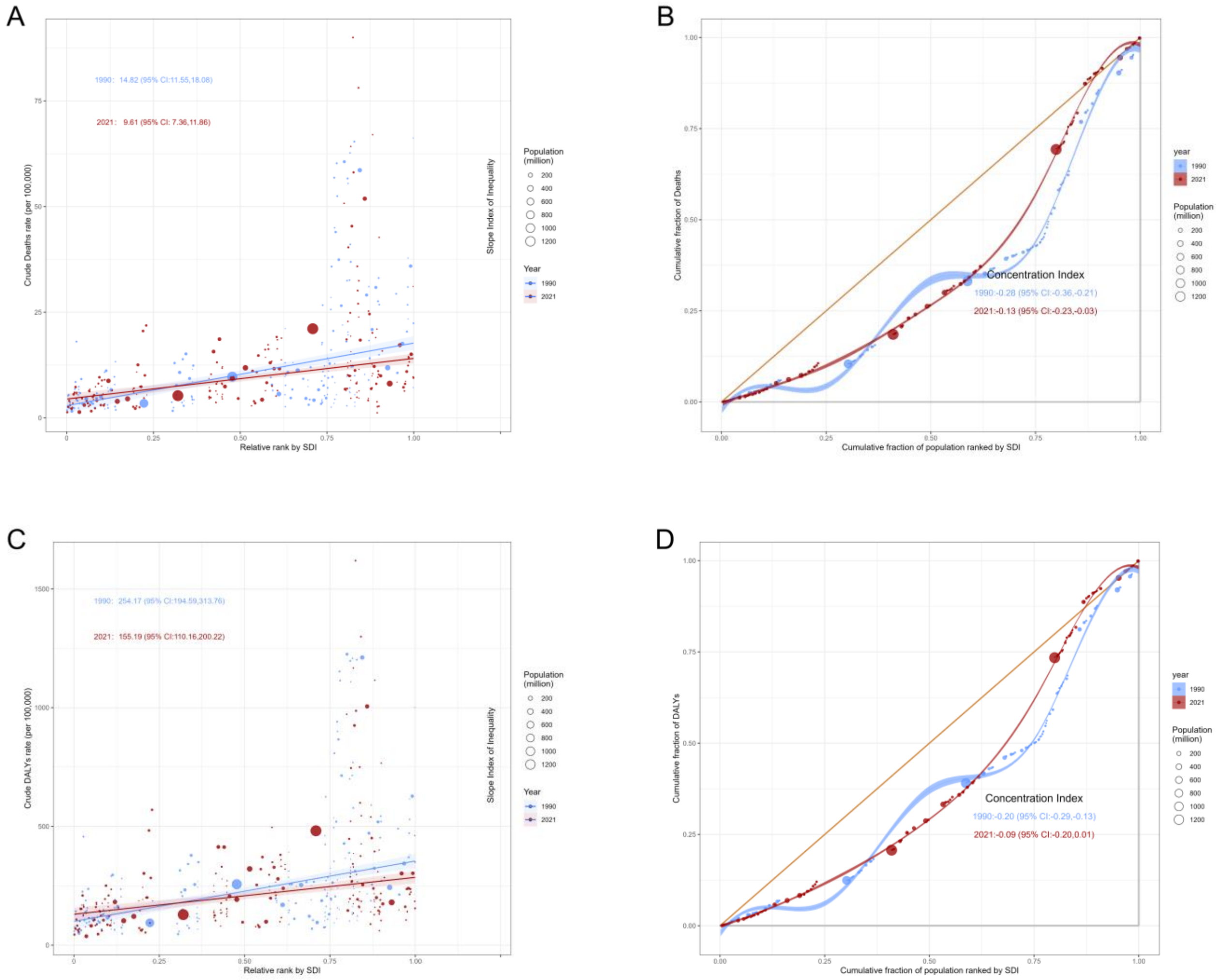

Fig. S9 Slope Index of Inequality curves and Concentration Index curves for deaths and DALYs due to ischemic stroke attributable to high LDL cholesterol, 1990 and 2021. **(A)** Slope Index of Inequality curves for crude deaths rate of ischemic stroke. **(B)** Concentration Index curves for Deaths of ischemic stroke. **(C)** Slope Index of Inequality curves for crude DALYs rate of ischemic stroke. **(D)** Concentration Index curves for DALYs of ischemic stroke. DALYs Disability-Adjusted Life Years

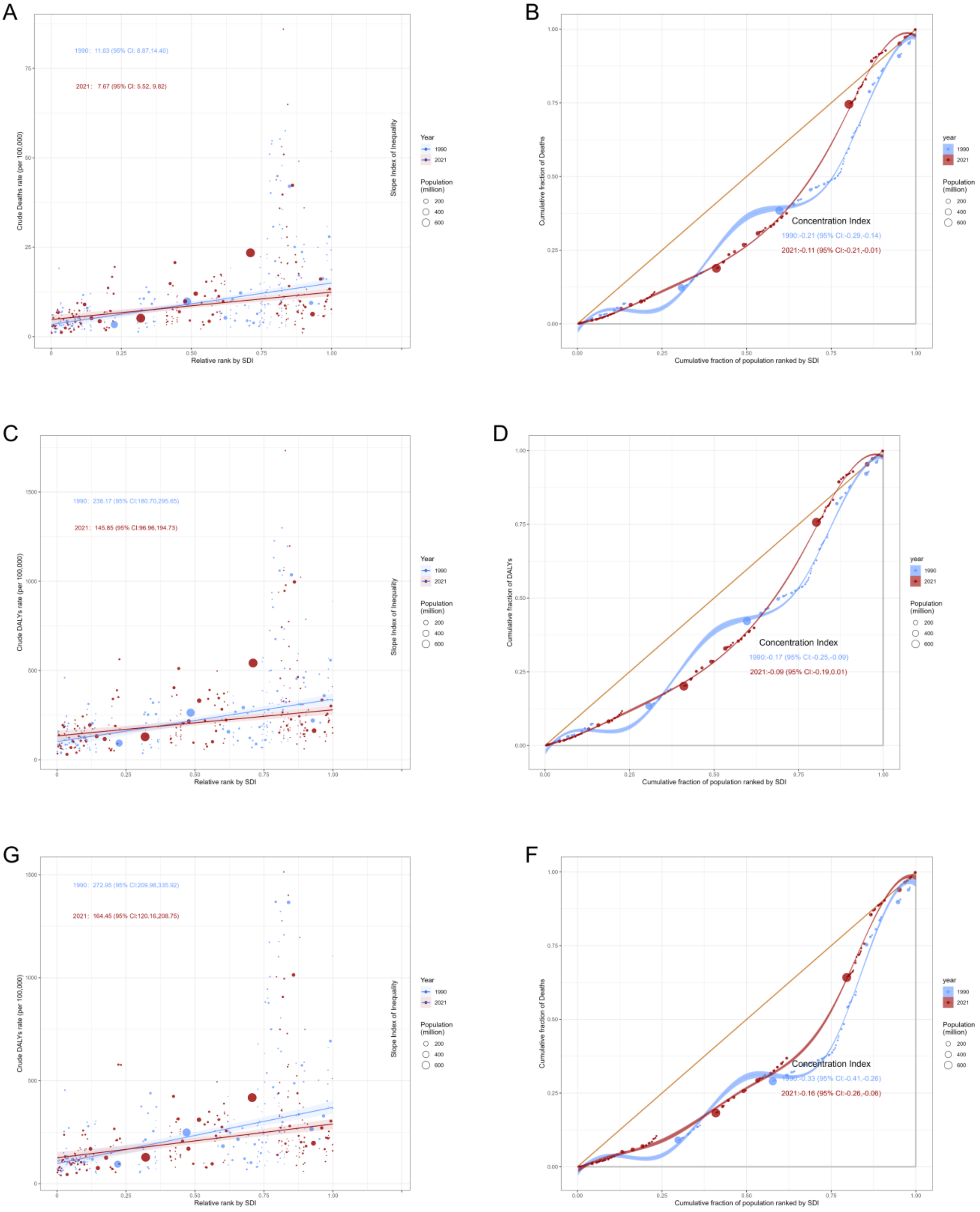
A
1990: 11.63 (95% CI: 8.87,14.40)
2021: 7.67 (95% CI: 5.52, 9.82)
Crude Deaths rate (per 100,000)
Relative rank by SDI
Slope Index of Inequality
Year
1990
2021
Population (million)
200
400
600
B
Cumulative fraction of Deaths
Cumulative fraction of population ranked by SDI
Concentration Index
1990:-0.21 (95% CI:-0.29,-0.14)
2021:-0.11 (95% CI:-0.21,-0.01)
year
1990
2021
Population (million)
200
400
600
C
1990: 238.17 (95% CI:180.70,295.65)
2021: 145.85 (95% CI:96.96,194.73)
Crude DALYs rate (per 100,000)
Relative rank by SDI
Slope Index of Inequality
Year
1990
2021
Population (million)
200
400
600
D
Cumulative fraction of DALYs
Cumulative fraction of population ranked by SDI
Concentration Index
1990:-0.17 (95% CI:-0.25,-0.09)
2021:-0.09 (95% CI:-0.19,0.01)
year
1990
2021
Population (million)
200
400
600
G
1990: 272.95 (95% CI:209.98,335.92)
2021: 164.45 (95% CI:120.16,208.75)
Crude DALYs rate (per 100,000)
Relative rank by SDI
Slope Index of Inequality
Year
1990
2021
Population (million)
200
400
600
F
Cumulative fraction of Deaths
Cumulative fraction of population ranked by SDI
Concentration Index
1990:-0.33 (95% CI:-0.41,-0.26)
2021:-0.16 (95% CI:-0.26,-0.06)
year
1990
2021
Population (million)
200
400
600

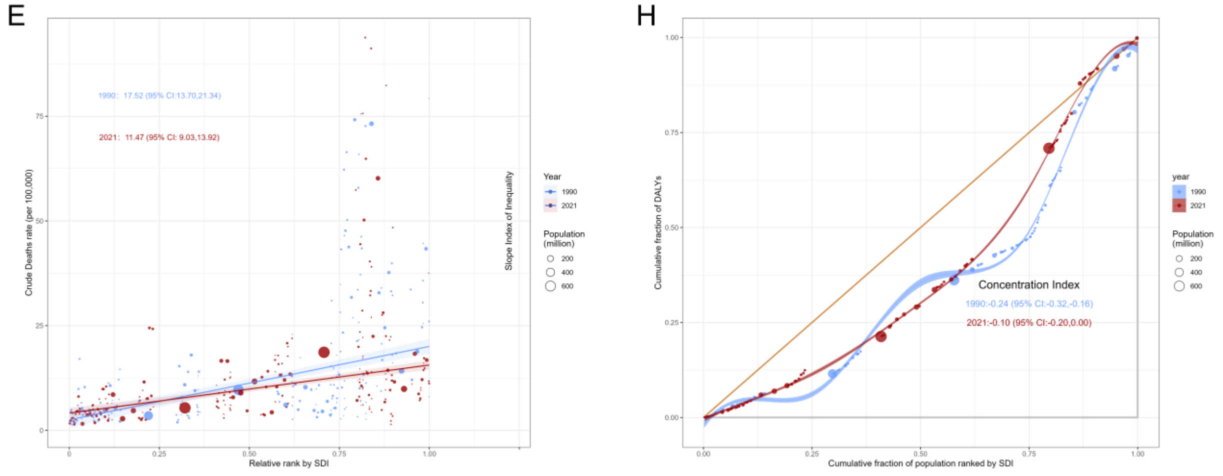

Fig. S10 Slope Index of Inequality curves and Concentration Index curves for deaths and DALYs due to ischemic stroke attributable to high LDL cholesterol, 1990 and 2021. **(A)** Slope Index of Inequality curves for the crude death rate of ischemic stroke in males. **(B)** Concentration Index curves for Deaths of ischemic stroke in males. **(C)** Slope Index of Inequality curves for crude DALYs rate of ischemic stroke in males. **(D)** Concentration Index curves for DALYs of ischemic stroke in males. **(E)** Slope Index of Inequality curves for the crude death rate of ischemic stroke in females. **(F)** Concentration Index curves for Deaths of ischemic stroke in females. **(G)** Slope Index of Inequality curves for crude DALYs rate of ischemic stroke in females. **(H)** Concentration Index curves for DALYs of ischemic stroke in females. DALYs Disability-Adjusted Life Years.

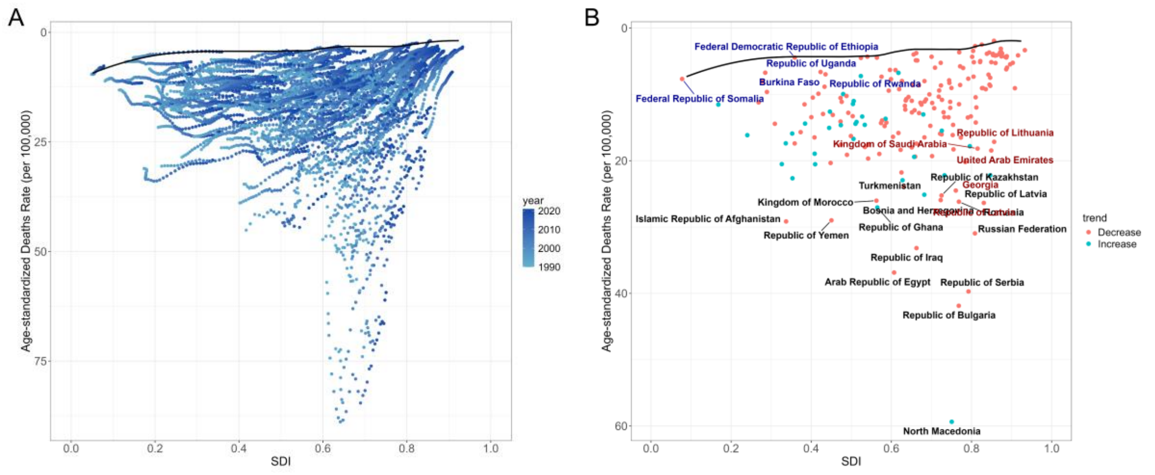

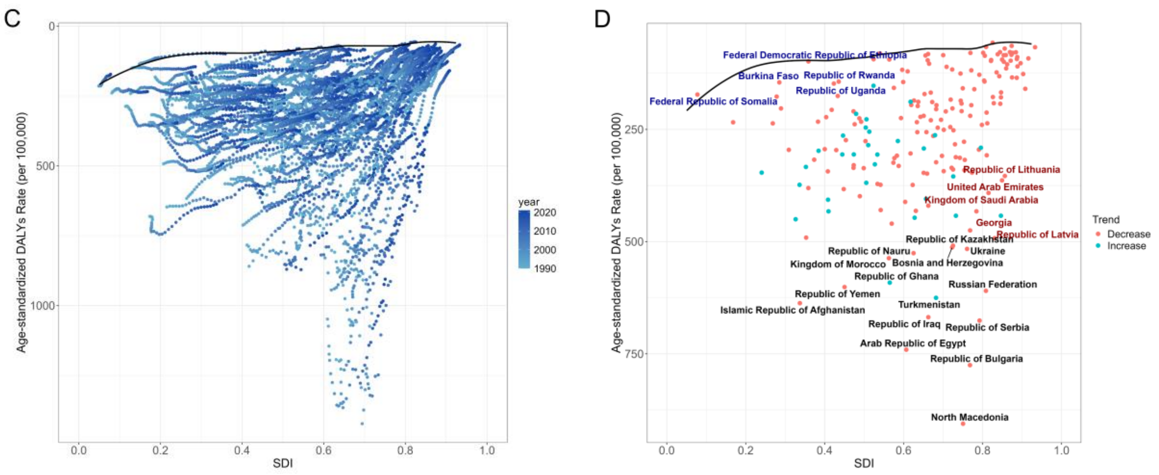

Fig. S11 Frontier analysis based on age-standardized rates of deaths and DALYs for ischemic stroke attributable to high LDL cholesterol and the SDI over the decades (1990–2021), with a specific focus on 2021.

Figures **A** and **C** illustrate the frontier analysis based on ASMR, ASDR, and SDI from 1990 to 2021. The color gradient transitions from light blue (1990) to dark blue (2021), with the black solid line delineating the boundary.

Figures **B** and **D** present the frontier analysis for 2021 based on ASMR, ASDR, and SDI. The black solid line marks the boundary, while the dots represent countries and regions. The 15 countries and regions with the largest effective differences are highlighted in black. Countries with low SDI (<0.466) and lower effective differences are marked in blue, whereas those with high SDI (>0.810) and relatively high effective differences are marked in red. The red dots indicate a decrease in ASMR or ASDR, while blue dots signify an increase in ASMR or ASDR from 1990 to 2021.

DALYs Disability-Adjusted Life Years, ASMR Age-Standardized Mortality Rate, ASDR Age-Standardized Disability-Adjusted Life Year Rate, SDI Socio-Demographic Index.

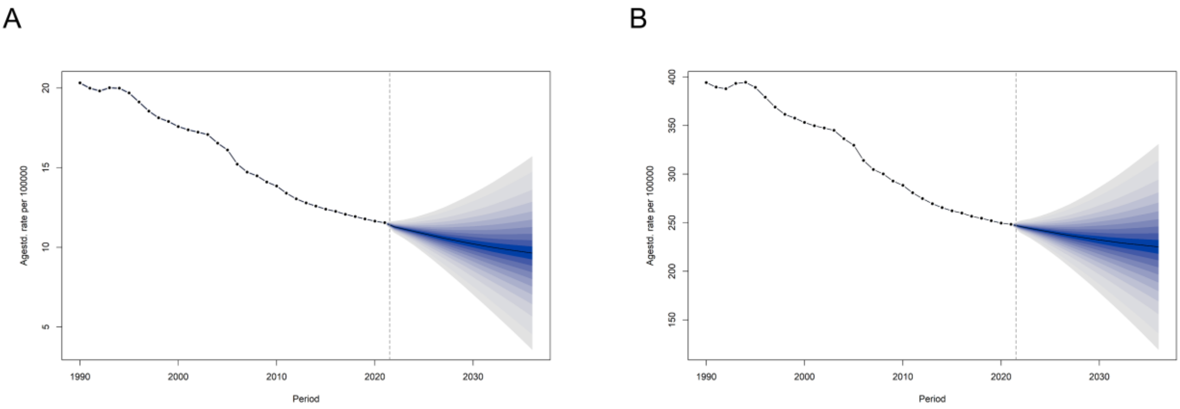

Fig. S12 Prediction of the global disease burden for ischemic stroke attributable to high LDL cholesterol until 2036. **(A)** Prediction of the global disease burden for ischemic stroke attributable to high LDL cholesterol until 2036, based on ASMR. **(B)** Prediction of the global disease burden for ischemic stroke attributable to high LDL cholesterol until 2036, based on ASDR.

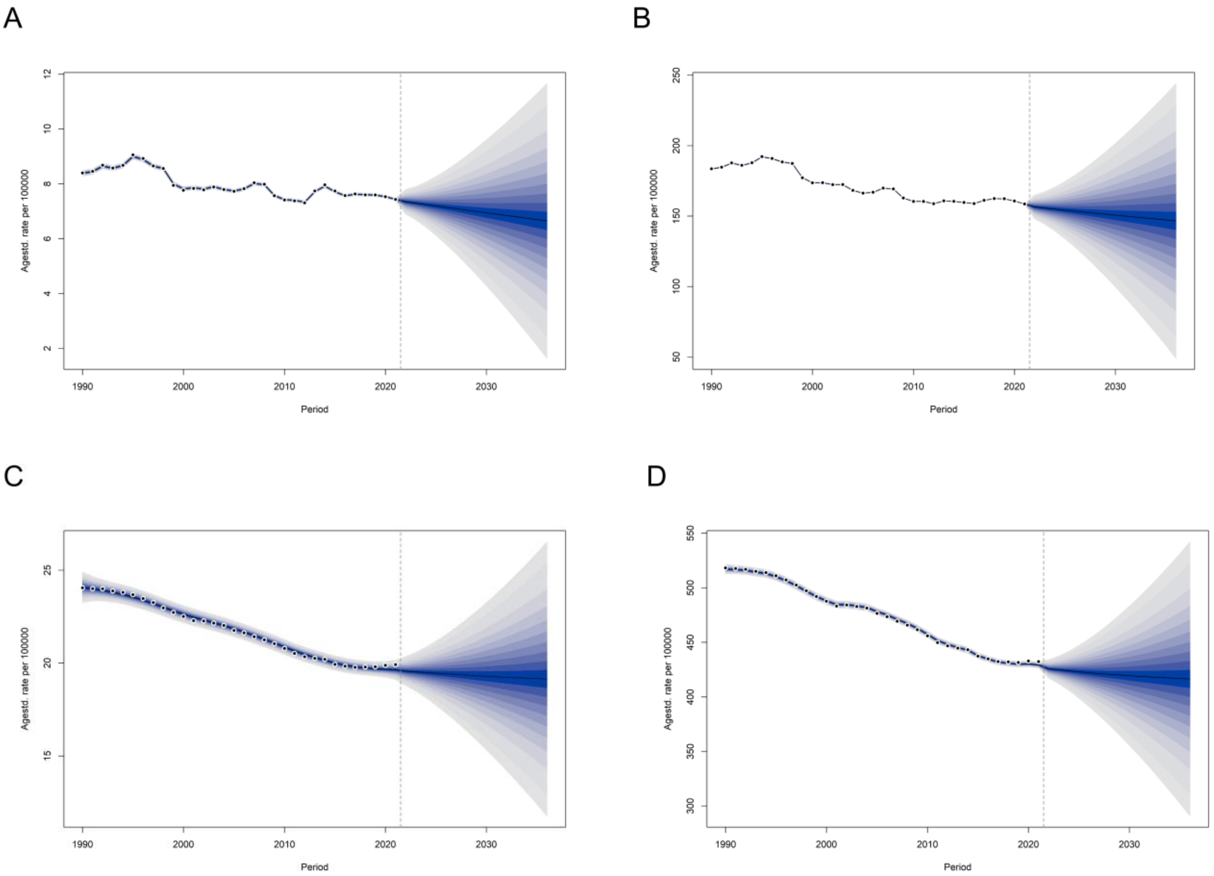

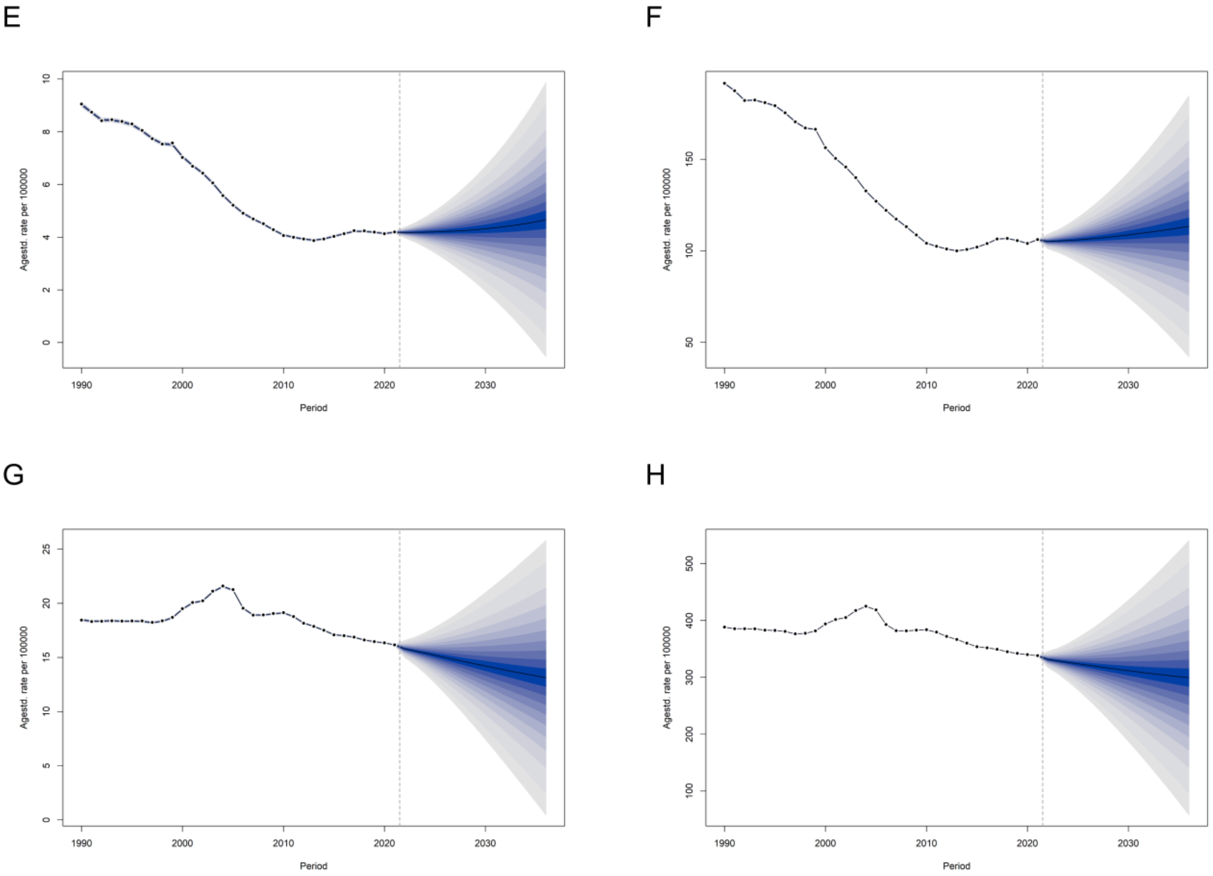

Fig. S13 Prediction of the disease burden for ischemic stroke attributable to high LDL cholesterol in various countries until 2036. **(A)** Prediction of the disease burden in India for ischemic stroke attributable to high LDL cholesterol until 2036, based on ASMR. **(B)** Prediction of the disease burden in India for ischemic stroke attributable to high LDL cholesterol until 2036, based on ASDR. **(C)** Prediction of the disease burden in Sudan for ischemic stroke attributable to high LDL cholesterol until 2036, based on ASMR. **(D)** Prediction of the disease burden in Sudan for ischemic stroke attributable to high LDL cholesterol until 2036, based on ASDR. **(E)** Prediction of the disease burden in America for ischemic stroke attributable to high LDL cholesterol until 2036, based on ASMR. **(F)** Prediction of the disease burden in America for ischemic stroke attributable to high LDL cholesterol until 2036, based on ASDR. **(G)** Prediction of the disease burden in China for ischemic stroke attributable to high LDL cholesterol until 2036, based on ASMR. **(H)** Prediction of the disease burden in China for ischemic stroke attributable to high LDL cholesterol until 2036, based on ASDR. ASMR Age-Standardized Mortality Rate, ASDR Age-Standardized Disability-Adjusted Life Year Rate.

A

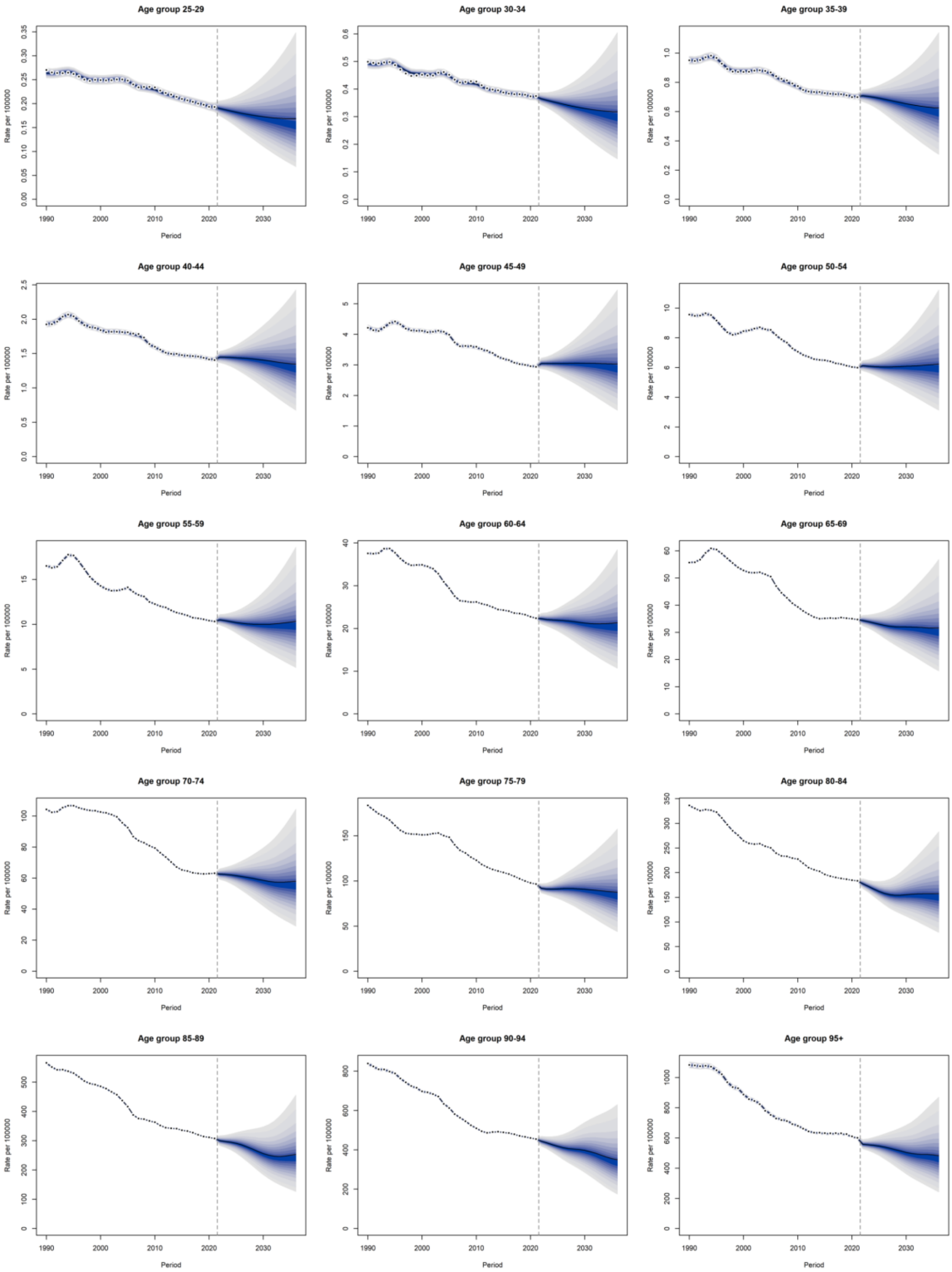

Age group 25-29
Age group 30-34
Age group 35-39
Age group 40-44
Age group 45-49
Age group 50-54
Age group 55-59
Age group 60-64
Age group 65-69
Age group 70-74
Age group 75-79
Age group 80-84
Age group 85-89
Age group 90-94
Age group 95+
Rate per 100000
Period

B

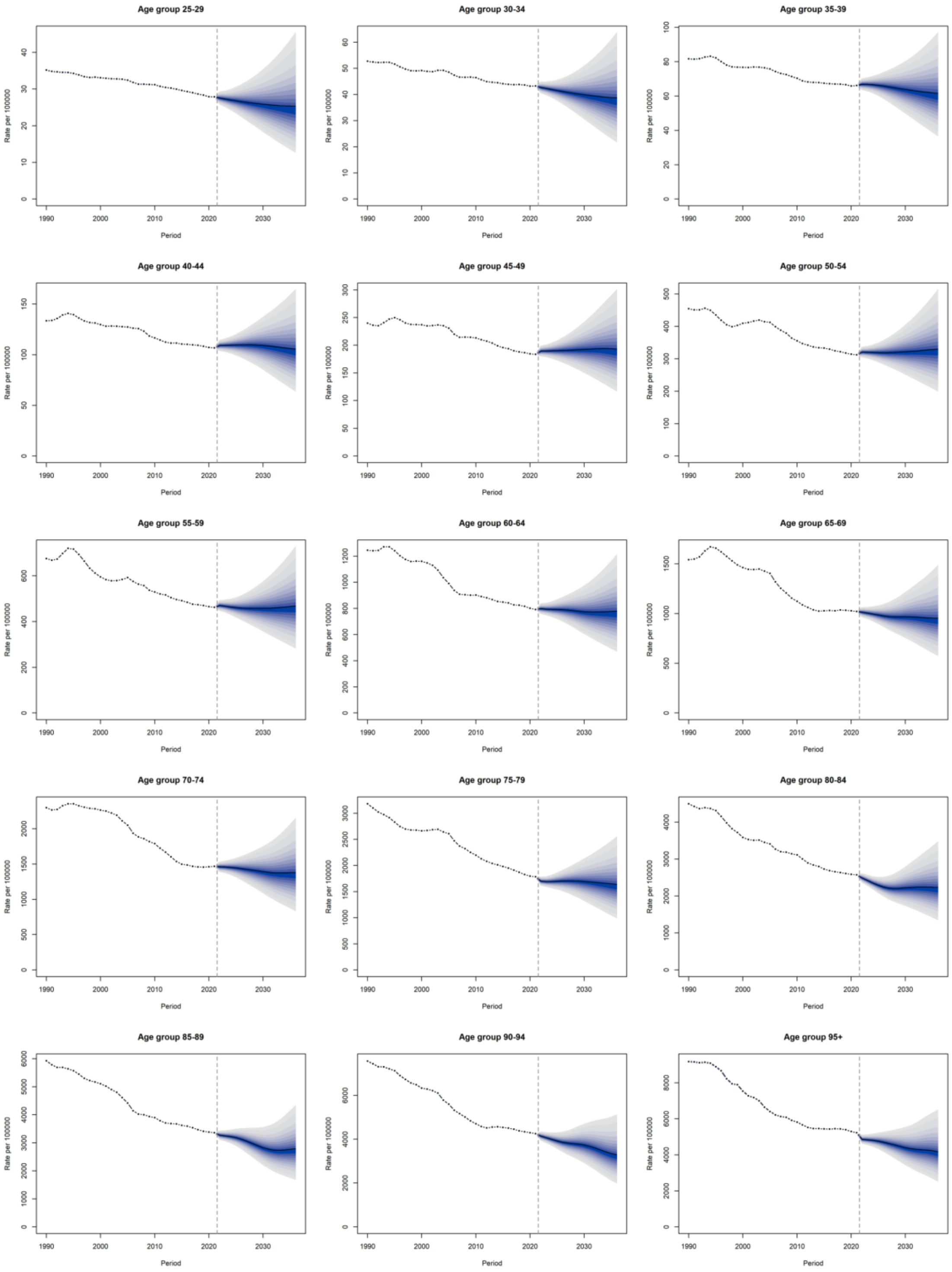
Age group 25-29
Age group 30-34
Age group 35-39
Age group 40-44
Age group 45-49
Age group 50-54
Age group 55-59
Age group 60-64
Age group 65-69
Age group 70-74
Age group 75-79
Age group 80-84
Age group 85-89
Age group 90-94
Age group 95+
Rate per 100000
Period

C

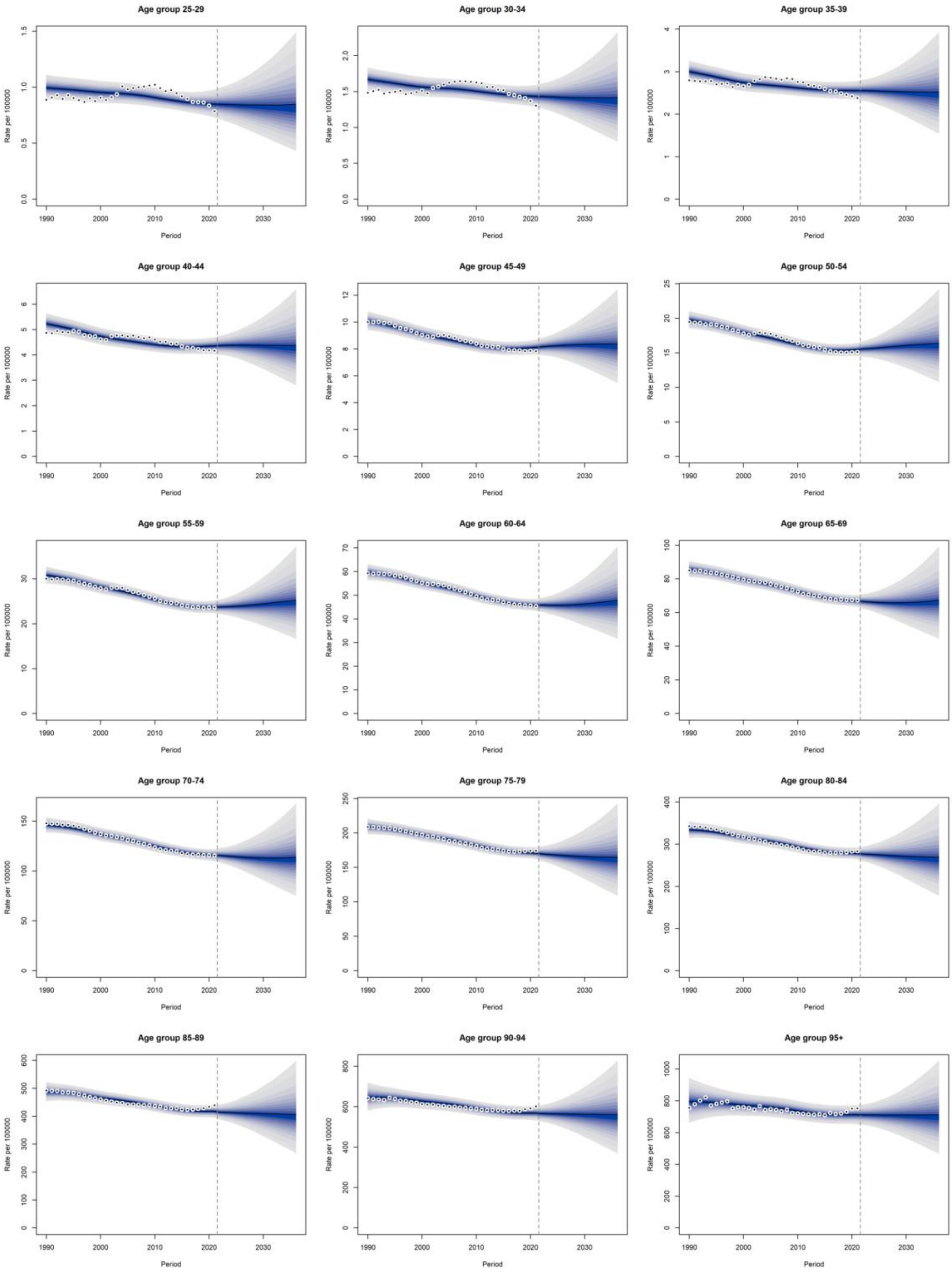
Age group 25-29
Age group 30-34
Age group 35-39
Age group 40-44
Age group 45-49
Age group 50-54
Age group 55-59
Age group 60-64
Age group 65-69
Age group 70-74
Age group 75-79
Age group 80-84
Age group 85-89
Age group 90-94
Age group 95+
Rate per 100000
Period

D

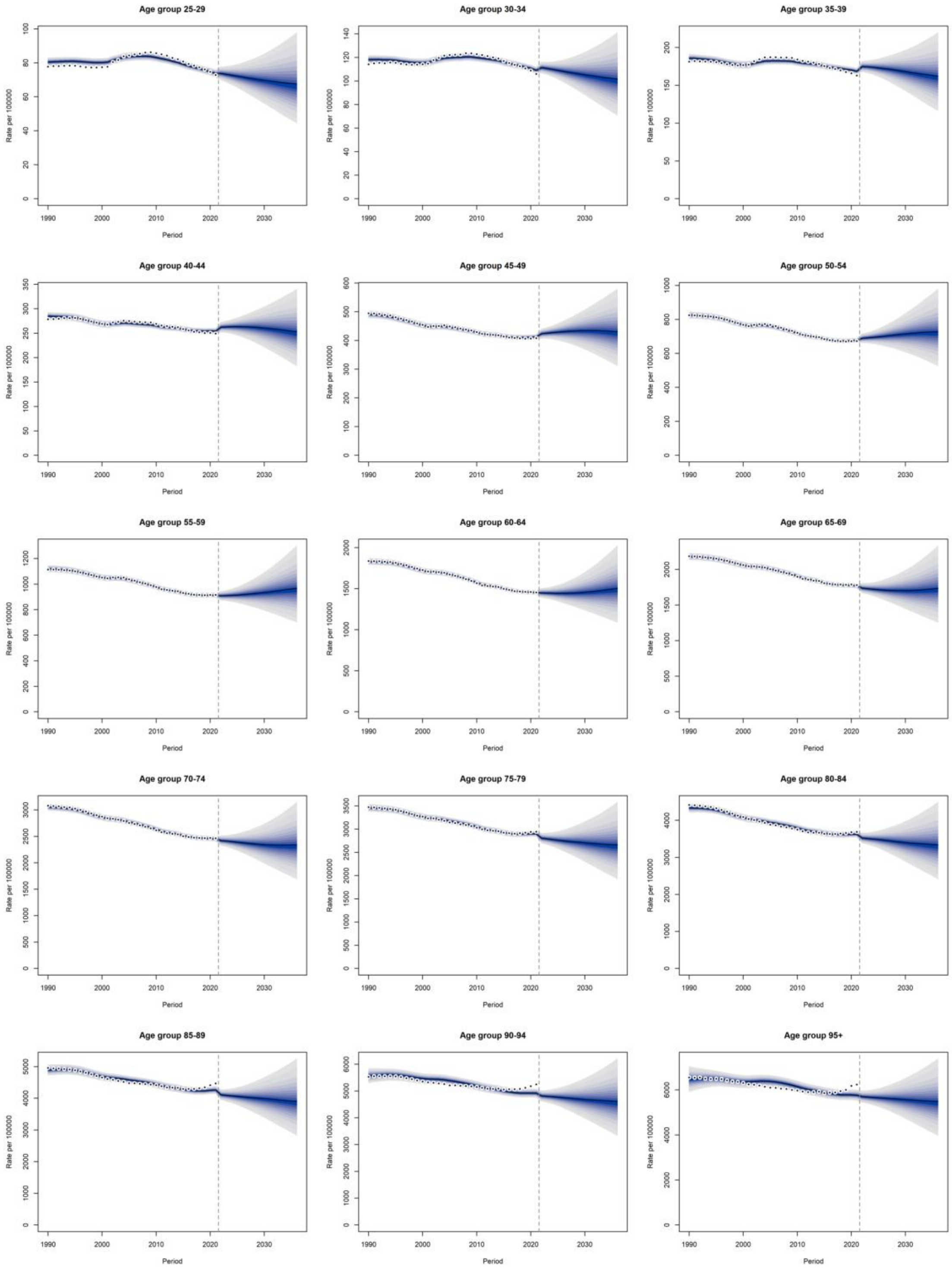

Age group 25-29
Age group 30-34
Age group 35-39
Age group 40-44
Age group 45-49
Age group 50-54
Age group 55-59
Age group 60-64
Age group 65-69
Age group 70-74
Age group 75-79
Age group 80-84
Age group 85-89
Age group 90-94
Age group 95+
Rate per 100000
Period
1990
2000
2010
2020
2030

E

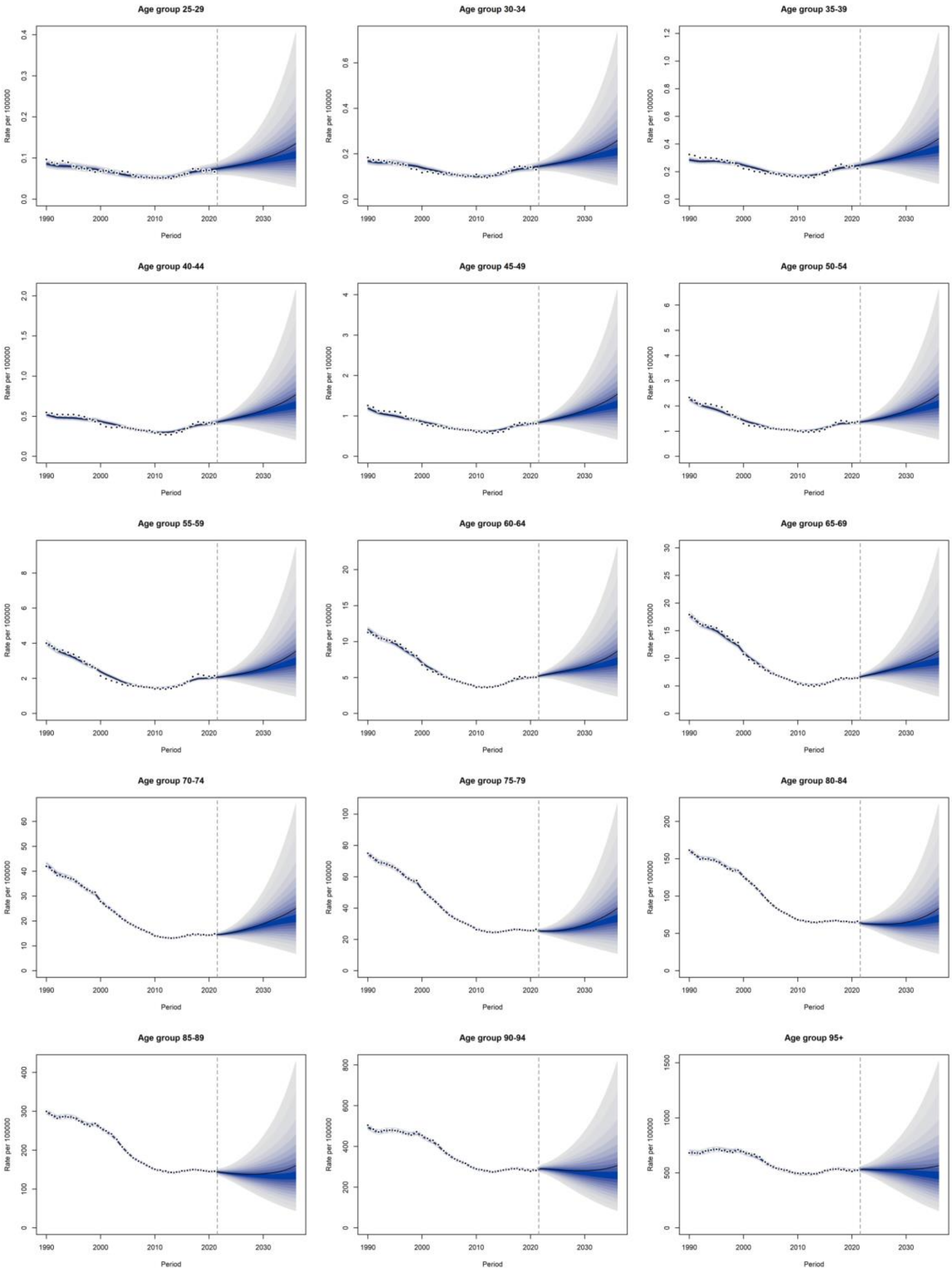
Age group 25-29
Age group 30-34
Age group 35-39
Age group 40-44
Age group 45-49
Age group 50-54
Age group 55-59
Age group 60-64
Age group 65-69
Age group 70-74
Age group 75-79
Age group 80-84
Age group 85-89
Age group 90-94
Age group 95+
Rate per 100000
Period
1990
2000
2010
2020
2030

F

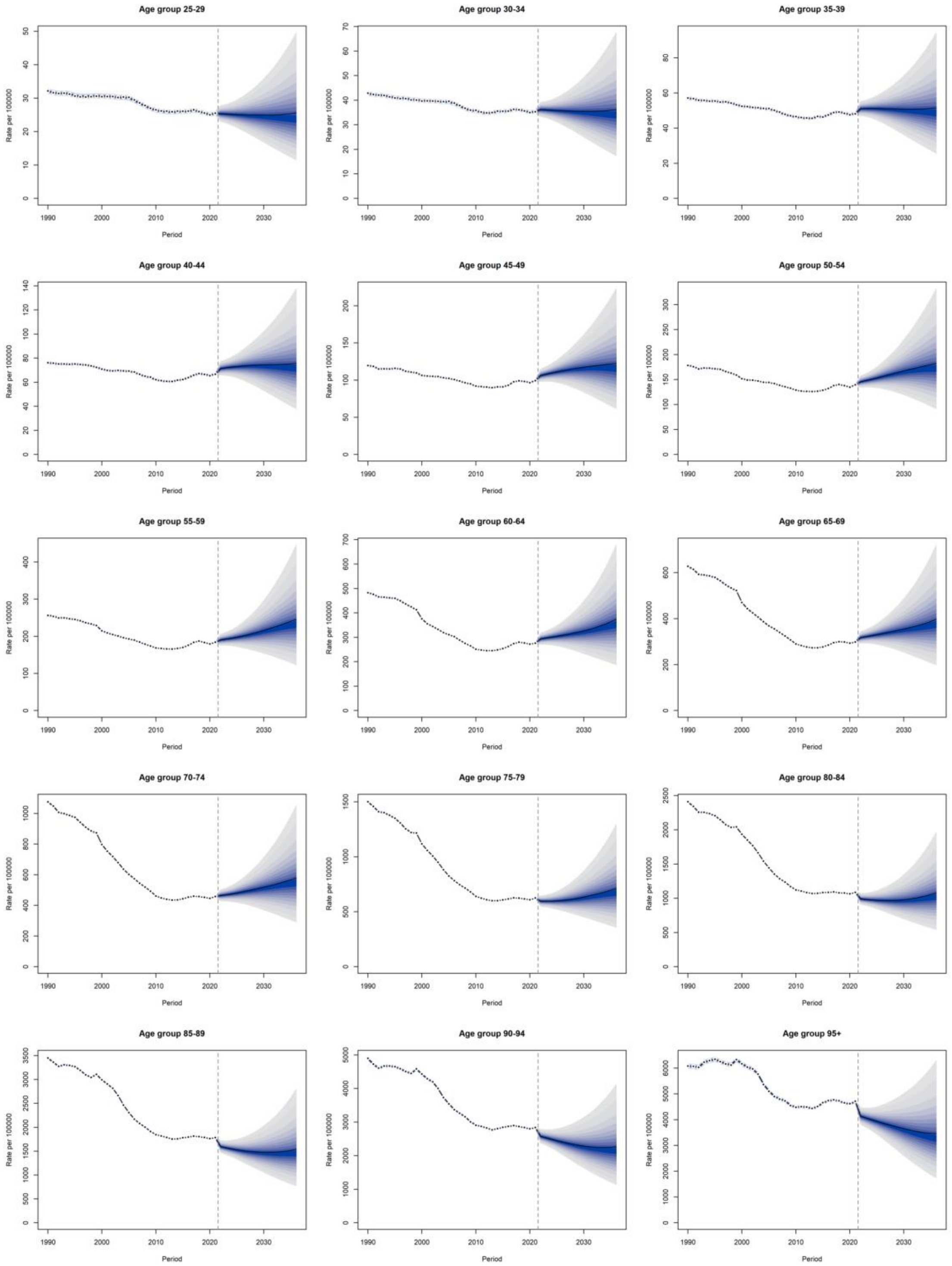

Age group 25-29
Age group 30-34
Age group 35-39
Age group 40-44
Age group 45-49
Age group 50-54
Age group 55-59
Age group 60-64
Age group 65-69
Age group 70-74
Age group 75-79
Age group 80-84
Age group 85-89
Age group 90-94
Age group 95+
Rate per 100000
Period

G

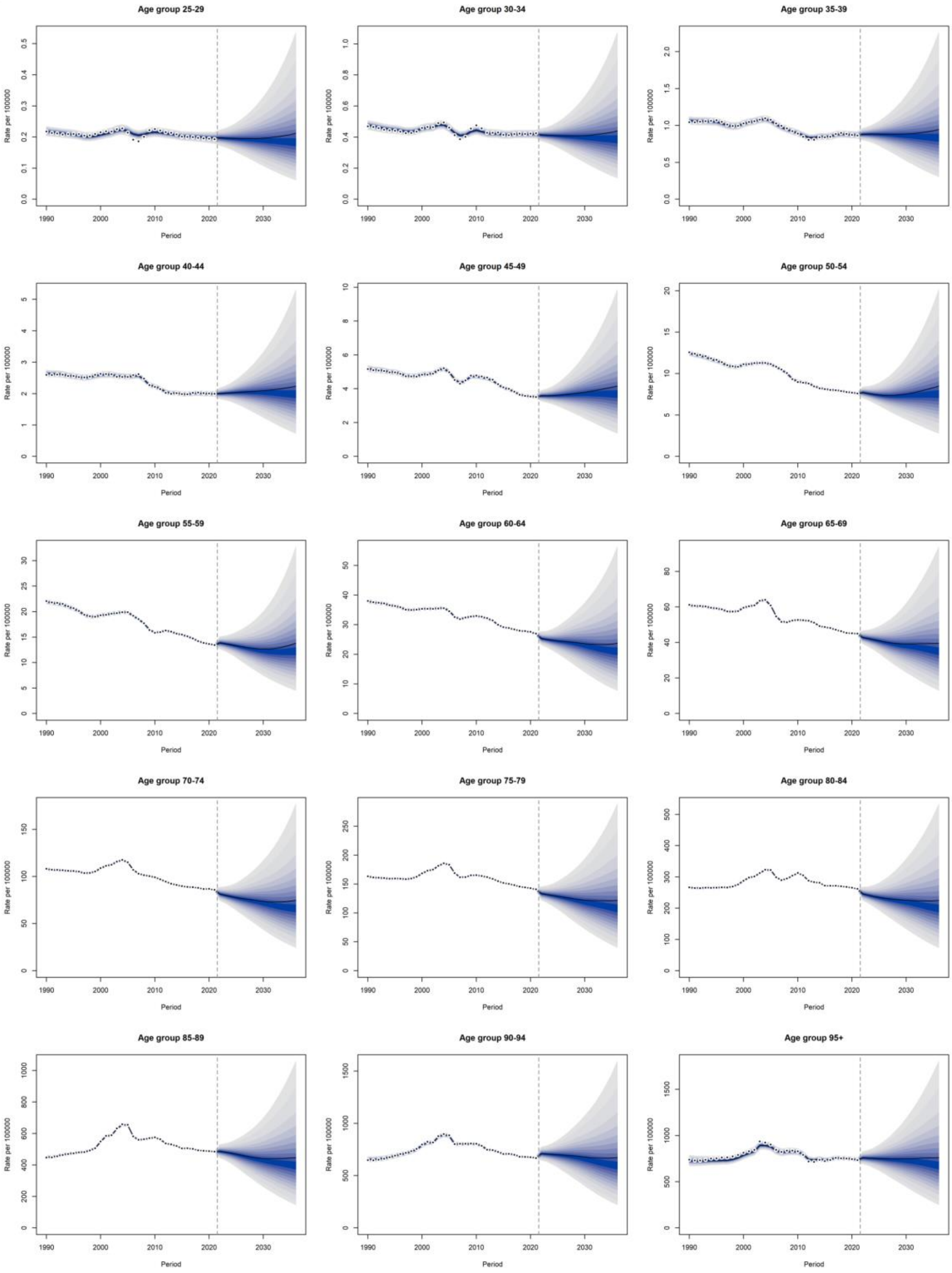
Age group 25-29
Age group 30-34
Age group 35-39
Age group 40-44
Age group 45-49
Age group 50-54
Age group 55-59
Age group 60-64
Age group 65-69
Age group 70-74
Age group 75-79
Age group 80-84
Age group 85-89
Age group 90-94
Age group 95+
Rate per 100000
Period
1990
2000
2010
2020
2030

H

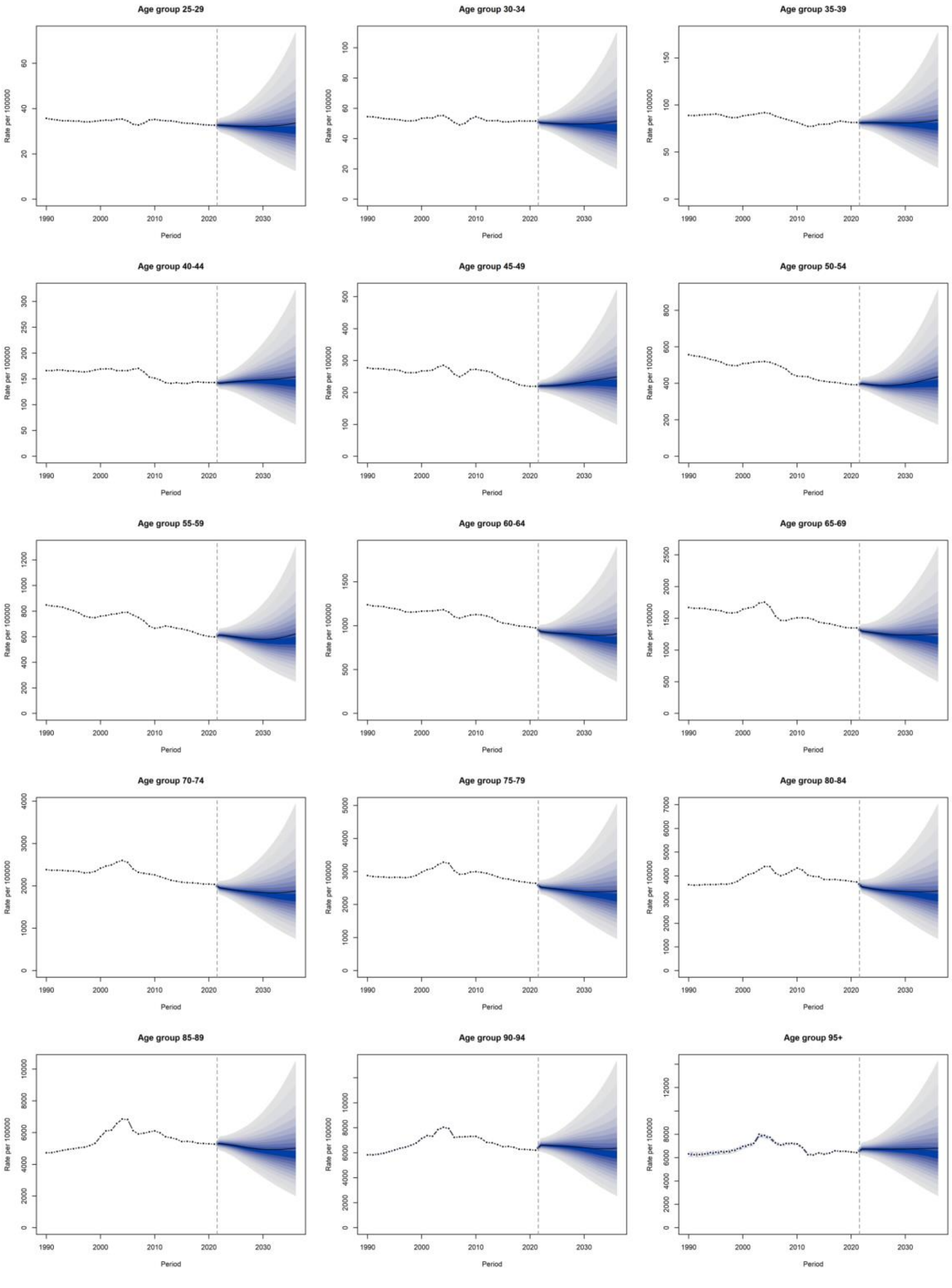
Age group 25-29
Age group 30-34
Age group 35-39
Age group 40-44
Age group 45-49
Age group 50-54
Age group 55-59
Age group 60-64
Age group 65-69
Age group 70-74
Age group 75-79
Age group 80-84
Age group 85-89
Age group 90-94
Age group 95+
Rate per 100000
Period

I

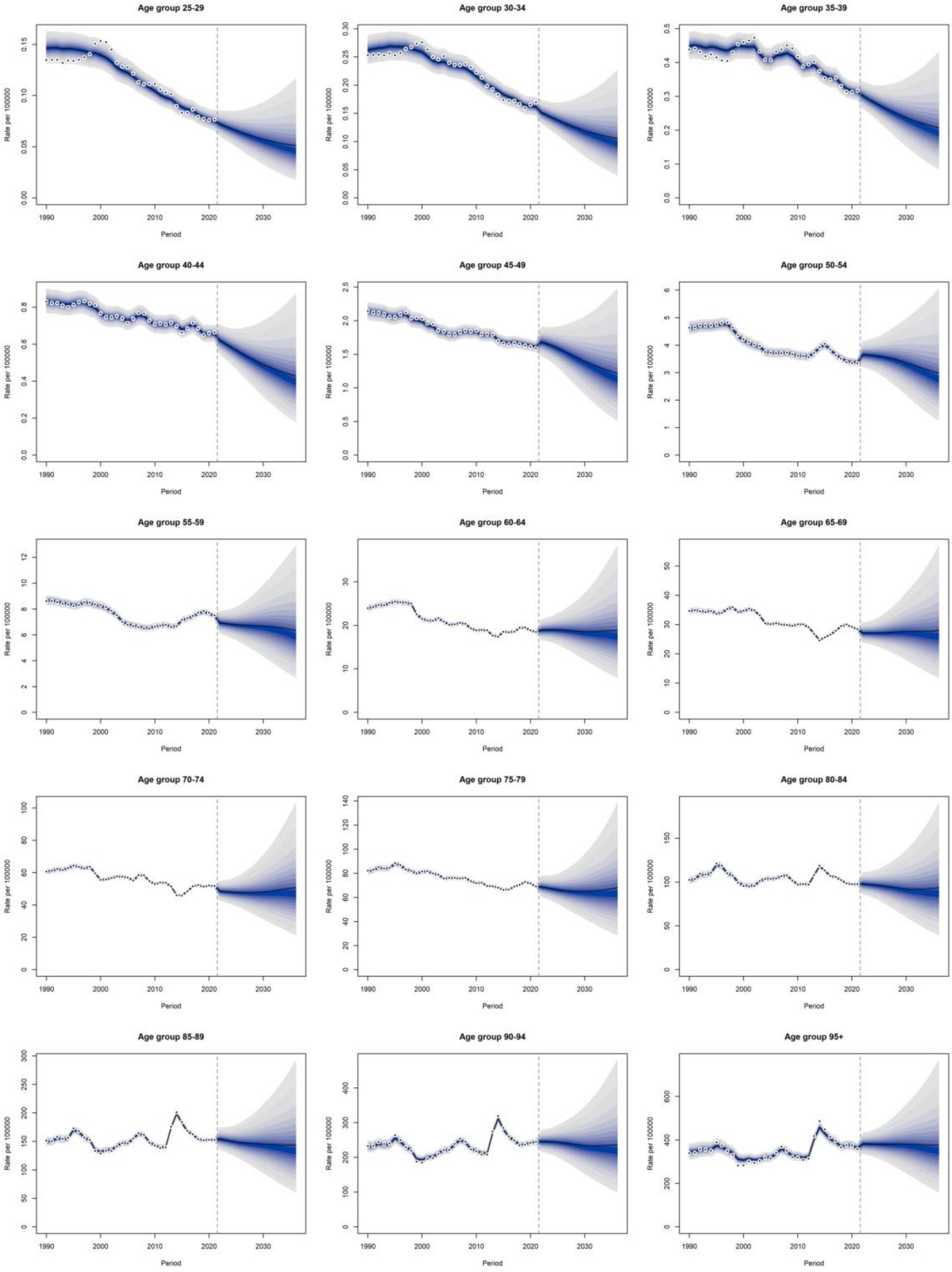

Age group 25-29
Age group 30-34
Age group 35-39
Age group 40-44
Age group 45-49
Age group 50-54
Age group 55-59
Age group 60-64
Age group 65-69
Age group 70-74
Age group 75-79
Age group 80-84
Age group 85-89
Age group 90-94
Age group 95+
Rate per 100000
Period

J

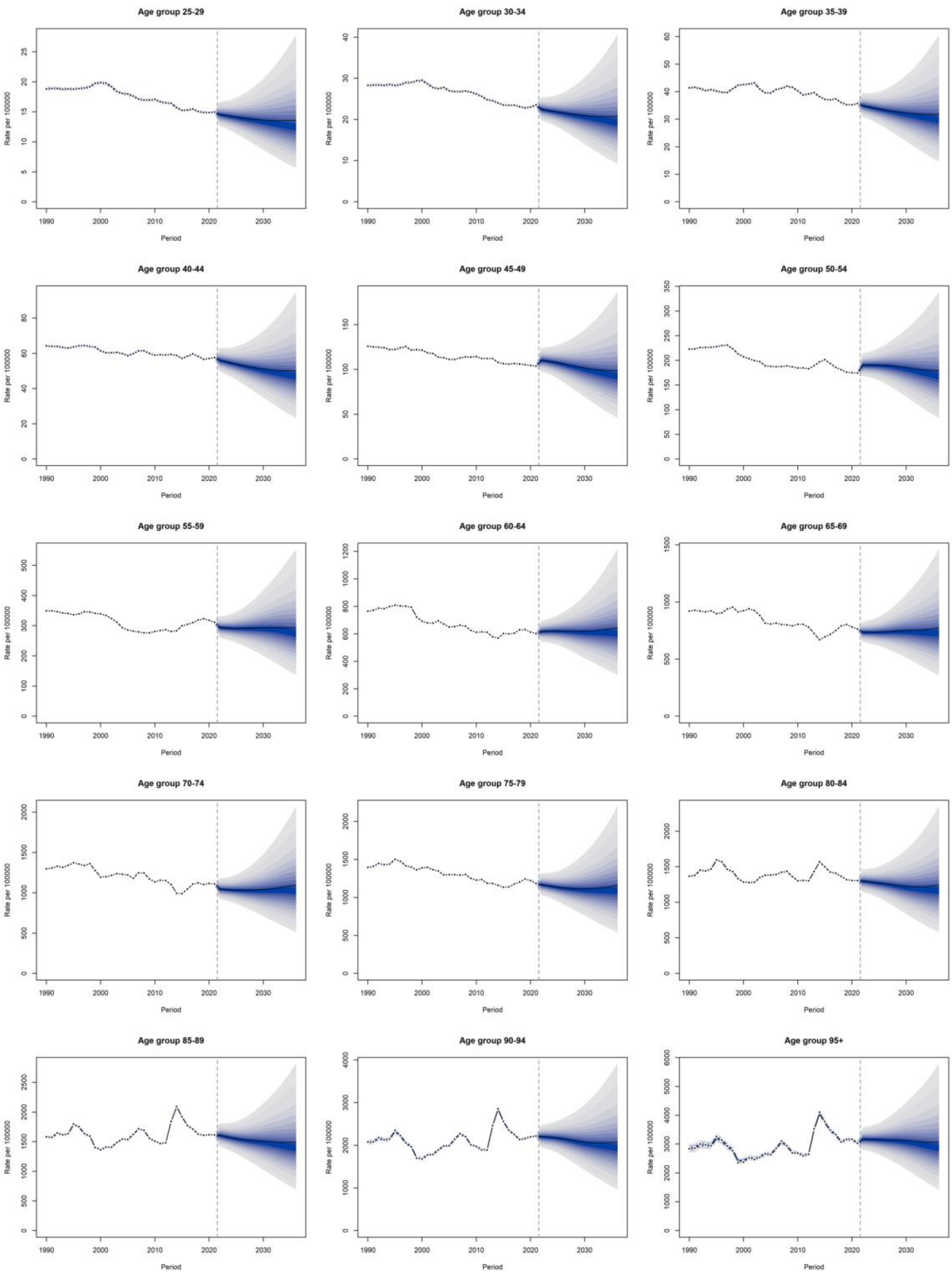

Fig. S14 Prediction of the disease burden for ischemic stroke attributable to high LDL cholesterol across different age groups until 2036. **(A)** Prediction of the global disease burden for ischemic stroke attributable to high LDL cholesterol until 2036, based on ASMR. **(B)** Prediction of the global disease burden for ischemic stroke attributable

to high LDL cholesterol until 2036, based on ASDR. **(C)** Prediction of the disease burden in Sudan for ischemic stroke attributable to high LDL cholesterol until 2036, based on ASMR. **(D)** Prediction of the disease burden in Sudan for ischemic stroke attributable to high LDL cholesterol until 2036, based on ASDR. **(E)** Prediction of the disease burden in America for ischemic stroke attributable to high LDL cholesterol until 2036, based on ASMR. **(F)** Prediction of the disease burden in America for ischemic stroke attributable to high LDL cholesterol until 2036, based on ASDR. **(G)** Prediction of the disease burden in China for ischemic stroke attributable to high LDL cholesterol until 2036, based on ASMR. **(H)** Prediction of the disease burden in China for ischemic stroke attributable to high LDL cholesterol until 2036, based on ASDR. **(I)** Prediction of the disease burden in India for ischemic stroke attributable to high LDL cholesterol until 2036, based on ASMR. **(J)** Prediction of the disease burden in India for ischemic stroke attributable to high LDL cholesterol until 2036, based on ASDR. ASMR Age-Standardized Mortality Rate, ASDR Age-Standardized Disability-Adjusted Life Year Rate.
